# Computational Modeling on Aquaporin-3 as Skin Cancer Target: A Virtual Screening Study

**DOI:** 10.3389/fchem.2020.00250

**Published:** 2020-04-15

**Authors:** Dharmendra Kumar Yadav, Surendra Kumar, Eun-Ha Choi, Sandeep Chaudhary, Mi-Hyun Kim

**Affiliations:** ^1^Gachon Institute of Pharmaceutical Science & Department of Pharmacy, College of Pharmacy, Gachon University, Incheon, South Korea; ^2^Plasma Bioscience Research Center/PDP Research Center, Kwangwoon University, Nowon-Gu, South Korea; ^3^Laboratory of Organic & Medicinal Chemistry, Department of Chemistry, Malaviya National Institute of Technology, Jaipur, India

**Keywords:** AQP3 protein, molecular docking, molecular dynamics, MM-GBSA analysis, pharmacophore-based filter

## Abstract

Aquaporin-3 (AQP3) is one of the aquaglyceroporins, which is expressed in the basolateral layer of the skin membrane. Studies have reported that human skin squamous cell carcinoma overexpresses AQP3 and inhibition of its function may alleviate skin tumorigenesis. In the present study, we have applied a virtual screening method that encompasses filters for physicochemical properties and molecular docking to select potential hit compounds that bind to the Aquaporin-3 protein. Based on molecular docking results, the top 20 hit compounds were analyzed for stability in the binding pocket using unconstrained molecular dynamics simulations and further evaluated for binding free energy. Furthermore, examined the ligand-unbinding pathway of the inhibitor from its bound form to explore possible routes for inhibitor approach to the ligand-binding site. With a good docking score, stability in the binding pocket, and free energy of binding, these hit compounds can be developed as Aquaporin-3 inhibitors in the near future.

## Introduction

The skin cancer represents a foremost and emergent public health problem, accounting for ~40% of all newly diagnosed cancer cases (Jemal et al., 2003). Skin cancer includes squamous cell carcinomas (SCC), basal cell carcinomas (BCC), and malignant melanomas (Einspahr et al., 2002). SCC and BCC are both non-melanoma skin cancers, originating from epidermal keratinocytes, and are associated with chronic sun exposure, while melanoma skin cancer arises from melanocytes and has sporadic sun exposure (Elwood et al., 1985; Katalinic et al., 2003; Lauth et al., 2004). The stratum corneum (SC) is the epidermal layer of the skin, which consists of terminally differentiated keratinocytes and contains an extracellular matrix of lipids. The skin's appearance and physical properties depend on a number of variables, including the composition of lipids/proteins, membrane properties, and water-retaining osmolyte concentration or “natural moisturizing factors” such as ions, free amino acids, and other small solutes (Elias, 2012). The aquaporins (AQPs) are ubiquitous family of small, hydrophobic, and strongly preserved membrane proteins involved in water transport and small solutes such as glycerol, nitrates, and urea (Verkman and Mitra, 2000; Fujiyoshi et al., 2002). To date, 13 human AQP isoforms (AQP0-12) have been identified and differentially expressed in many types of cells and tissues in the body (Hara-Chikuma and Verkman, 2008c).

The AQPs are broadly classified into two groups: orthodox aquaporins (selective for water) and aquaglyceroporins (permeable to small solutes including glycerol) (Takata et al., 2004). Genotype and phenotype studies have established their role in obesity, brain swelling, glaucoma, epilepsy, refractory edema, cancer, neuroinflammation, and pain (Verkman, 2012). In cell migration, the presence of AQPs has associated them in local invasion, tumor angiogenesis, and metastasis (Verkman et al., 2008). Among all the identified AQP isoforms, AQP1 (expressed in endothelial cells) and AQP3 (expressed in the basal layer of keratinocytes in human skin) are of particular interest for the study of cancer model (Hara-Chikuma and Verkman, 2008a; Verkman et al., 2014). The functions of AQPs in the skin have not been thoroughly investigated; however, over the last few decades, AQP3 has gained attention as it is abundantly expressed in the skin (Sugiyama et al., 2001; Hara-Chikuma and Verkman, 2008b). AQP3 (aquaglyceroporins) transport water, glycerol, urea, and hydrogen peroxide, and plays a major role in SC hydration, skin elasticity, cell proliferation, wound healing, cell migration and tumorigenesis (Hara-Chikuma and Verkman, 2008a). Previous studies have reported that deficient mice with Aquaporin-3 (AQP3) may have delayed barrier recovery and dry skin due to the absence of AQP3 facilitated glycerol transport (Hara et al., 2002; Hara and Verkman, 2003). Hara-Chikuma and Verkman (2008b) have studied a multistage skin tumor model in mice and reported that AQP3 is overexpressed in skin cancer, while AQP-null mice show complete resistance to development of skin cancer. Huang et al. (2015) reported that epidermal growth factor and estrogen contribute to the development of cancer and are upstream regulators of AQP3 expression. Since cancer cells have elevated levels of H_2_O_2_, AQP3-mediated H_2_O_2_ transport plays an important role in the development of cancer (Lennicke et al., 2015). AQP3-mediated H_2_O_2_ transport increases phosphorylation of the protein kinase B (Akt) and the extracellular signal-regulated kinase (Erk) 1/2. Likewise, overexpressed AQP3 increases the MMPs (matrix-metalloproteases), which further promote the cancer cell invasiveness (Marlar et al., 2017). Verkman et al. (2008) states that, in epidermal hyperproliferation conditions, such as ichthyosis, wound healing, atopic dermatitis, tumorigenesis, and psoriasis, overexpressed AQP3 is found. Thus, AQP3-facilitated glycerol transport generates ATP and mediates the growth and survival of tumor cells. By targeting AQP3 expression reduces several intracellular signaling pathways, leading to reduced cell proliferation, migration, and invasion (Aikman et al., 2018).

**Figure 1:**
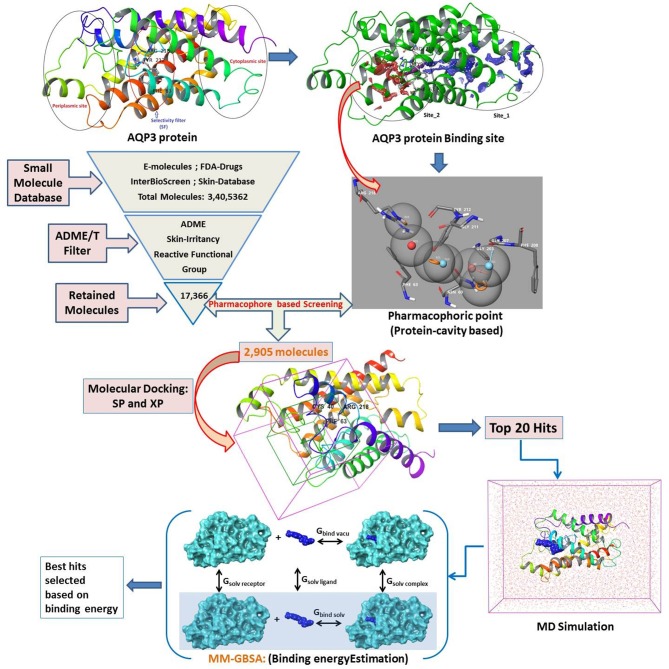
Designed workflow for the virtual screening of AQP3 inhibitors.

There are compelling possibilities in the quest for AQP-based treatment, yet little progress has been made so far. A few reported inhibitors of AQP are appropriate for clinical trials, none of them showed any specificity for AQP3 (Niemietz and Tyerman, 2002; Migliati et al., 2009; Brown and Lu, 2013). AQP1, a close congener of AQP3 in terms of protein sequence, reported to be inhibited by tetraethylammonium salts (Brooks et al., 2000), acetazolamide (Bing et al., 2004), bumetanide (Kourghi et al., 2016), and DMSO (Vanhoek and Vanos, 1990). In addition, Preston et al. (1993) and Niemietz and Tyerman (2002) found mercurial (HgCl_2_), silver (Ag), and gold (Au) containing inorganic compounds functioning as selective AQP inhibitors (De Almeida et al., 2013). Martins et al. (2012) subsequently evaluated metal-based drugs already reported to have specific therapeutic properties for inhibition of AQP1 and AQP3 (such as antirheumatic, anticancer, and antibacterial agents) and reported promising results. Moreover, several authors have synthesized and reported gold-based compounds for AQP3 inhibitors and elucidated the mechanism of inhibition (Martins et al., 2013; De Almeida et al., 2014; Serna et al., 2014). Here for the first time, we explore small molecule inhibitors (hits) of AQP3 using a series of virtual screening tools, followed by molecular dynamics and binding free energy calculations (Figure 1). Virtual screening tools comprising molecular docking and pharmacophore-based methods may reduce false positives in potential hits.

## Materials and Methods

### Chemical Datasets and Virtual Screening Methods

The size, shape, and physicochemical properties of molecules governs the biopharmaceutical criteria (Potts and Guy, 1995; Van De Waterbeemd et al., 1998). Therefore, a series of filters comprising physicochemical (QikProp) (Yadav et al., 2010, 2012, 2013a,b, 2014a,b,c, 2016, 2017a,b, 2018a,b), toxicity (skin irritancy) (Saluja et al., 2014; Verma et al., 2020), and reactive functional group (Huggins et al., 2011; Tsaioun and Kates, 2011) were applied to narrow down the number of available drug molecules. The following filter conditions were set: Molecular Weight: 20–300; LogS (Predicted Solubility): −9.0 to 1.0; LogKp (Predicted Skin Permeability): −8.0 to 1.0; Jm (Predicted Transdermal Transport Rate): <10; Reactive Functional Groups: 0–2; Skin irritancy: low or none (Potts and Guy, 1992; Lian et al., 2008; Mitragotri et al., 2011). A total of 3,379,981 small molecules collected from the e-molecules database (https://www.emolecules.com), IBS database (synthetic and natural compounds) (https://www.ibscreen.com/bases), the dataset from Braga et al. (2017), and US-FDA approved drugs (Table 1) (https://www.fda.gov/Drugs/default.htm) were passed through the series of filters as mentioned above. In addition, pharmacophore-based (protein cavity) (Loving et al., 2009) screening and molecular docking-based (Standard Precision and Extra Precision) (Friesner et al., 2004) screening were performed to find good scoring compounds. From these good scoring compounds, the top 20 hits were selected for desmond molecular dynamics (Bowers et al., 2006) and MM-GBSA based binding free energy prediction (thermal_mmgbsa.py) (Greenidge et al., 2013).

**Table 1:** Small molecules considered under the present study.

**Database/literature**	**Total number of molecules**
E-molecules	3,32,8465
InterBioScreen (IBS)	67,609
Small molecules from http://chembench.mml.unc.edu	87
US-FDA approved drugs	9,101

### Ligand Preparation

The structures were prepared using the LigPrep module in Schrodinger suite (Maestro11.6). Using an OPLS3 force field the LigPrep produces energy minimized 3D structures using. For each structure, the tautomer, correct Lewis structure, and ionization states (pH 7.0 ± 2.0) were generated, optimized, and energy minimized under default settings.

### Protein Preparations, Active Site Prediction, and Receptor Grid Generation

The crystal structure of AQP3 has not been solved and thus obtained from the reported work of Martins and coworkers (Martins et al., 2012). They used homology modeling tools to build an AQP3 structure. The model structure was imported into the maestro workspace and the multistep Protein Preparation Wizard was used to correct the protein structure, that includes addition of H-atoms, bond order correction, and H-bond network optimization, followed by energy minimization using Impref module of Impact with an OPLS3 force field (Sastry et al., 2013).

**Figure 2:**
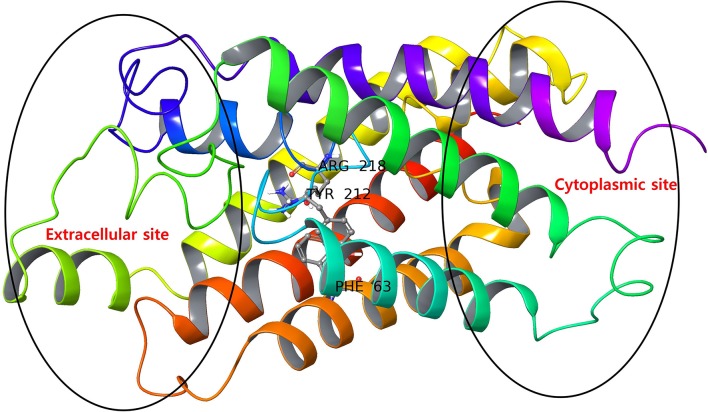
AQP3 protein with extracellular and cytoplasmic sites. The SF region comprising key amino acid residues (Phe63, Tyr212, and Arg218) are displayed as ball and stick representations.

The AQP3 has a tunnel-like structure with extracellular and cytoplasmic pore sites at opposite ends, separated by a selectivity filter (SF) domain consisting of conserved amino acid residues (Phe63, Arg218, and Tyr212), the domain being a distinguishing features defining AQPs subfamilies (Figure 2) (Savage et al., 2010; Martins et al., 2012). Martins et al. reported that AQP3 has an prolonged hydrophobic area in the vicinity of the SF domain of a extracellular pocket, which is absent in AQP1 (another member of aquaglyceroporins), providing the latter with higher hydrophilic character (Park and Saier, 1996).

The prepared structure lacks any bound ligand, so the binding sites were defined using SiteMap tools of Schrodinger (Halgren, 2009) with default settings. The AQP3 structure with identified sites is shown in Figure S1. The SiteMap tool has identified two probable binding sites and considering the domain of conserved amino acid residues Site_2 was selected as the binding site and a receptor grid was generated for molecular docking.

### Pharmacophore-Based (Protein Cavity) Filtering

**Figure 3:**
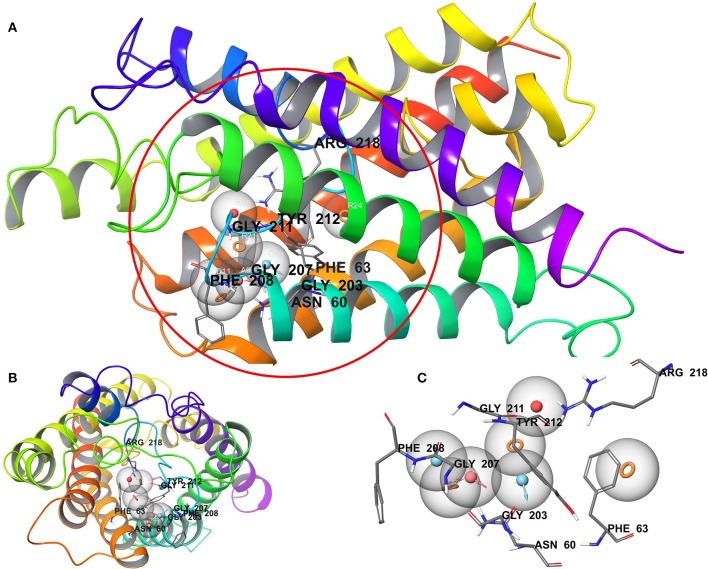
**(A)** Whole protein showing cavity-based pharmacophoric point; **(B)** top view with pharmacophoric point; **(C)** pharmacophoric point with label residues in the binding pocket.

We have employed the pharmacophore filtering methods of Phase module of Schrodinger Suite to further screen the small molecules. Numerous approaches have been available and described in detail elsewhere (Chang et al., 2006; Peach and Nicklaus, 2009; Planesas et al., 2011). The homology model of AQP3, lack of a bound ligand thus, we employed a protein cavity-based pharmacophore point enumeration method. Since Site_2 was identified as binding site, it was further utilized to enumerate the pharmacophoric point for the pharmacophore-based filtering method with following selected settings; maximum number of features: 7; Donors as vectors; remove non-contributing fragments; create receptor-based excluded volume shell; radii size set to Van de Waals radii of receptor atoms and 0.5 as scaling factor. The 5.0 Å was set for excluded volume shell thickness. The pharmacophore filtering methodology is based on docking of fragments to a protein receptor (e-pharmacophore model), followed by a selection of fragment features that maximize the binding interaction. The Common features identified by pharmacophoric points were selected to satisfy criteria for their positions and directions (Figure 3). Finally, the identified pharmacophoric points were selected as query and all the molecules in the database were filtered and ranked according to the PhaseScreenScore. The PhaseScreenScore measures the quality and quantity of features matching to the hypothesis.

### Molecular Docking

The filtered small molecules from the pharmacophore-based filtering method were further analyzed by molecular docking. The molecular docking was performed with glide v7.8 in the SP (Standard Precision) and XP (Xtra Precision) protocol of the Schrodinger Suite with default settings (Friesner et al., 2004). The Standard Precision protocol was first adopted to discriminate the binders and non-binders. A threshold criteria (docking score > −6.0 kcal/mol) was set to select the hits from SP docking and then further used for XP docking.

### Molecular Dynamics

All the MD simulations were carried out using the package Desmond 5.3 (Bowers et al., 2006). The protein-ligand complex system was inserted into the pre-equilibrated POPC lipid bilayer membrane using the Set up Membrane option of system builder module of Desmond. The upper and lower lipid bilayer region of system was filled with water model (TIP3P) (Mark and Nilsson, 2001) as the solvent in orthorhombic box with OPLS_2005 force-field. The shape (orthorhombic) and size was set at 10 Å buffered distance. The final size of the system in all three direction was 52.15 × 52.01 × 10.62 Å. The desired neutral system was built with the addition of 0.15 M NaCl in the system. A built system is shown in Figure S2. The system was energy minimized by Steepest Descent and the Broyden-Fletcher-Goldfarb-Shanno (BFGS) algorithms in a hybrid manner (Saputro and Widyaningsih, 2017). The simulation was performed under NPT ensemble using the Nose-Hoover thermostat (Evans and Holian, 1985) and Martyna-Tobias-Klein barostat methods (Cho et al., 1993; Yadav et al., 2018a,b, 2019, 2020a,b,c; Kumar et al., 2020). Applying a constant temperature of 300° K and 1.01325 atm of pressure, respectively. The short-range coulombic interactions were analyzed with a cut-off value of 9.0 Å. The smooth particle mesh ewald method (Essmann et al., 1995) was used to handle long-range coulombic interactions and RESPA-based constraints allowing 2 fs of time steps. The MD simulation was conducted for 100 ns. The MD simulation analysis was performed using simulation interaction diagram, simulation even analysis and simulation quality analysis tools from desmond program.

### Binding Free Energy Analysis

The interaction energies between the target protein and the selected top poses were computed using the MM-GBSA (generalized-born/surface area) method implemented in Schrodinger (Shivakumar et al., 2010; Li et al., 2011). The average binding free energy (ΔG_bind) based on MM-GBSA was calculated using the thermal_mmgbsa.py script. During the MM-GBSA calculation, the last 10 ns MD simulation trajectory (100 snap shot) was used as input to compute the average binding free energy.

### Ligand-Unbinding Pathway

**Figure 4:**
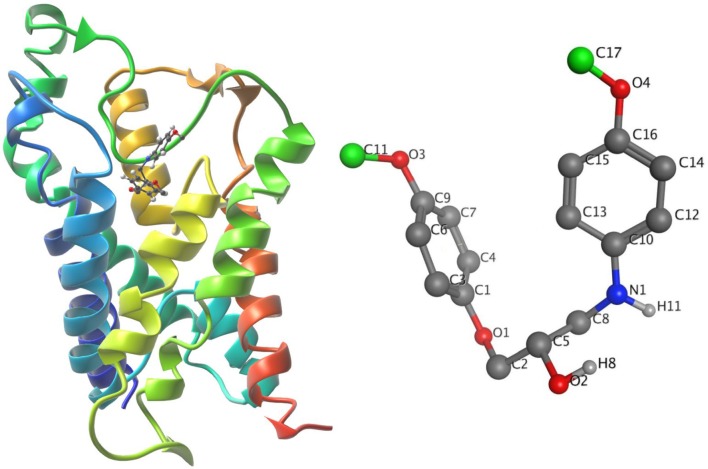
(Left) The system in ribbons with bound ligand in ball and stick form. (Right) The ligand with active atoms in green.

The ligand-unbinding pathway was further explored using the ART-RRT method (Nguyen et al., 2018) implemented in SAMSON. The ligand-unbinding pathways were searched on the last frame from dynamic simulations of the protein-ligand complex. In this study, we used the compound 5633879 [1-(4-methoxyphenoxy)-3-((4-methoxyphenyl)amino)propan-2-ol] to explore its unbinding path from the binding pocket. The gromos53a6 force field (Oostenbrink et al., 2004) assigned to the protein and ligand parameter was obtained from the ATB (Automated Topology Builder) server (Malde et al., 2011). The ligand atom shown in green was assigned to active ARAP (as-rigid-as-possible) atoms and a box with a dimension of 100 × 100 × 120 Å was assigned during the pathway search (Figure 4). We performed 10 runs with default settings for the ligand-unbinding pathway search.

## Results and Discussion

### Chemical Database Curation and Ligand Preparation

The databases chosen in the present study had varying ranges of physicochemical and toxicological properties (Figure S3). Therefore, the selected database molecules were filtered based on a pre-condition filter (mentioned in the Materials and Methods section) to find the molecules that could easily permeate with no toxicological (skin irritancy) properties. This two-stage filter (ADME and Toxicity) greatly reduced the number of molecules, for further processing using the Schrodinger's LigPrep module.

### Pharmacophore-Based (Protein Cavity) Filtering

The pharmacophore-based (protein cavity) hypothesis, shown in Figure 3, depicts seven chemical features comprising three aromatic rings (R), two hydrogen bond donors (D), one hydrogen bond acceptor (A), and one negative ion (N). Among the features generated, hydrogen bond donor and acceptor features are vector properties possessing a vectorial nature, indicating the direction of the sharing of electrons (Dixon et al., 2006). The features in the hypothesis were superimposed on the Site_2 binding site, which revealed that the chemical features were complementary to key amino acid residues, including H-bonding interactions with Asn60, Gly203, Gly207, Gly211, and Arg218, corresponding to A, D, and N features. Likewise, Phe63, Phe208, and Tyr212 corresponded to R features. The reduced dataset molecules (after application of the initial physicochemical and toxicity filter) was further screened against the generated pharmacophore-based (protein cavity) hypothesis as a query and molecules were ranked according to the PhaseScreenScore. The molecules with PhaseScreenScore above 0.8 were selected for the next stage (i.e., molecular docking).

### Molecular Docking

**Figure 5:**
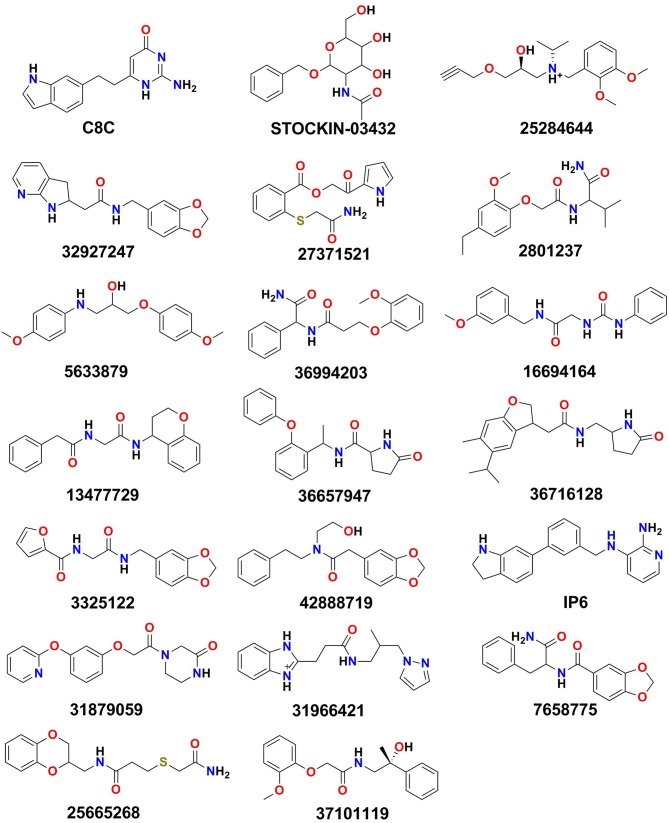
The top 20 hits obtained after XP docking.

The identified binding site (Site_02) encompasses the hydrophilic/hydrophobic areas comprising amino acid residues within 3 Å of the binding pocket, namely, Val43, Val46, Phe56, Ile59, Asn60, Phe63, Phe147, Ala148, Thr149, Tyr150, Gly207, Gly211, Tyr212, and Arg218. Martins et al. (2012) reported that among all the binding site residues, the triad amino acid residues (Phe63, Arg218, Tyr212) near the extracellular gate are involved in the binding of small molecules and can modulate the function of the AQP3 protein. Therefore, Site_02 was chosen as the binding site and used to dock small molecules obtained after pharmacophore-based (protein cavity) filter. From the XP docking result, the dock poses were ranked according to the docking score and the top 20 poses were selected as potential hits that could modulate the function of AQP3 protein (Table S1). The ligand-binding amino acid residues from the top 20 hits are summarized in Table S2 and pharmacophoric point elements found for these 20 hits are shown in Figure S4. Figure 5 shows the structure of all the top 20 hits (please refer Table S1 for each compounds chemical name and docking score values)

The top 20 poses were selected and analyzed from all the docked poses (XP mode), where they had docking scores ranging from −7.550 to −6.747 kcal/mol. The comparison of binding poses shows that despite the diverse scaffold of these poses, all interact with common binding site residues (Figure S5). All the docked poses displayed multiple direct hydrogen bond interactions with important amino acid residues, such as Asn60 and Arg218, while the backbone atoms of residues Gly145, Ala148, Gly207, Gly211, and Phe208 were involved in hydrogen bond interactions. Similarly, most of the compounds displayed π-π stacking interactions with the aromatic rings of the Tyr150 and Phe208 amino acid residues. Studies have stated that, the amino acid residues from the extracellular site and selectivity filter (SF) region (Phe63, Arg218, Tyr212), play an important role in ligand binding (Martins et al., 2012; De Almeida et al., 2017). Thus, the analysis of the top scoring docked poses revealed that, at the opening of extracellular site and near the SF region, the aromatic fragments of the molecules are juxtaposed into a group of aromatic amino acid residues (including Phe63, Tyr150, Phe208, and Tyr212) which reveal their importance in modulating the function of target protein.

### Molecular Dynamics

For molecular dynamics simulation, the top 20 docked poses were submitted to access the stability, explicit solvation, conformational adoption of the protein, and reliability of the docked poses at the atomic level. The efficiency of the molecular dynamics simulation was calculated by measuring the total energy, potential energy, temperature, pressure, and volume of the protein-ligand complexes (Figures S6–S25). The potential energy includes the sum of the bond, angle, torsion, and non-bonded terms represent the system stability. Therefore, the plots of potential energy clearly indicated that the system was well-equilibrated and remained stable throughout the simulations. Figures S25–S48 illustrated, the other measured structural parameters (i.e., RMSDs, Root Mean Square Fluctuations (RMSFs), and interaction histograms). The overall structural fluctuation of protein with bound ligand and without bound ligand was measured against simulation time by calculating the RMSDs of Cα-atoms. In all simulations the protein with bound ligand attains equilibrium within 10 ns of simulation, and then oscillates afterward with RMSDs below 3.5 Å, suggesting that the system has evolved into stable states and has been properly converged. Typically, the trajectories in each pose during the simulation generated a stable protein with an average RMSD value that ranged from 1.75 to 3.50 Å, where the large RMSDs found may occur due to motion of the loop region of the protein. The protein RMSDs without bound ligand were found to be higher than ligand bound protein, and did not converge throughout the simulation suggesting that, the overall protein conformation is conserved by binding of the ligand poses (Figures S26–S29). In addition, it was observed from the RMSDs plot that protein-ligand complexes did not dissociate and remained bound throughout the simulations.

Furthermore, in order to access the movement of residues during the simulation we plotted the RMSFs for Cα-atoms of all residues (see Supplementary File). The RMSFs plot displayed the helix (pink-colored bar region) and ligand contacts region (green-colored vertical bar) during the simulation. The RMSFs measure the average atomic mobility of the Cα-atoms and it was observed from the residue analysis that residues 49–55, 75–85, 94–100, 123–125, 134–156, 178–190, 211–220, 229–248, and 265–269 form part of the loop region and may be flexible during the simulations. The molecular docking analysis shows that top scored poses have interacted with target protein residues from the loop region (i.e., 55, 141, 142, 145-152, 211, 212, and 218). However, the ligand contact analysis of the loop region from the RMSFs plot revealed that, despite the flexible loop region, interactions from poses (STOCKIN-03432, 2801237, 16694164, 5633879, 13477729, 36716128, 42888719, 31879059, 25665268) were stable during the simulation (Figures S30a–S49a).

It is noteworthy to mention here that, all the selected 20 poses are structurally diverse with multiple rotatable bonds. Despite such structural flexibility, the ligand RMSDs, revealed that the compounds were relatively stable (Figures S50–S53). For example, CMPD17 (31966421) (Figure S53) contains a seven rotatable group and its RMSD, despite initial higher fluctuations, was stable ~1.5 Å. Likewise, CMPD09 (16694164) (Figure S51) with a eight rotatable group was stable around ~2.25 Å. Thus, it is assumed that compounds repositioned their binding mode during the initial simulation and then subsequently acquired stability.

From molecular docking analysis, it has been shown that all the docked poses make multiple interactions with the binding residues; thus, we have further investigated its stability over the simulation. Figures S30b,c–S49b,c display the histograms (interaction fraction) and 2D interactions between ligands and binding amino acid residues throughout the simulation obtained from simulation interaction analysis module of desmond program. The interaction fraction in the histogram shows different color bars, each of which represents the contribution of respective interactions with amino acid residues. The green, purple, red, and blue colored bars correspond to H-bonds, Ionic, Hydrophobic, and water bridge interactions, respectively. The 2D interaction diagram shows interacting residues that have been retained over 20% of the simulation time. Additionally, the total number of hydrogen bond established throughout the simulation between protein and ligand was also calculated and shown in Figures S30d–S49d. The total number of hydrogen bond observed ranges from 1 to 6 and these hydrogen bonds were retained throughout the simulation in most of the compounds, further stabilizing the compounds in the protein binding pocket. In addition, during the simulation, the protein secondary structure elements (SSE) such as α-helix and β-strands were observed in order to understand the stability of secondary structures. The (Figures S30e–S49e) displays the SSE composition over the course of the simulation for each trajectory frame and each residue. The visual inspection of SSE result shows that, during the molecular dynamic simulations, the secondary structure elements were stable. In any dynamic simulation, multiple forces act upon protein-ligand complexes resulting in the establishment of different types of molecular interactions. During simulation the dynamic interactions between the protein and the ligands are summarized and further compared with the interaction analysis from the molecular docking results (Table 2). Comparative analysis of poses from dynamics and docking simulation interactions revealed that either previously formed interactions (during docking) were retained or new interactions with amino acid residues were formed during dynamics. Among all the 20 dynamic simulations, compounds C8C, 42888719, and 31879059 failed, while the interactions observed during the docking simulation could be retained by other compounds, although some new interactions with all the poses were seen. In addition, we were also interested in observing the interactions during the simulation with key triad amino acid residues (Phe63, Tyr212, Arg218); and trajectory analysis revealed that all compounds except 25284644, 32927247, 36657947, 36716128, 42888719, IP6, and 31879059 interact with these key amino acid residues. Thus, the results of molecular dynamic simulation results support the docking simulation and suggest these molecules could be important modulators of the function of AQP3.

**Table 2:** Comparison of amino acid residue interactions observed during Molecular Dynamic simulations and Molecular Docking (bold faces represent the common binding amino acid residues).

Compound ID^*^	**Amino acid residues interaction observed during simulation**	**Amino acid residues interaction observed during docking**
C8C	Val43 (H-Bond with Backbone), Phe56 (pi-pi), Gly211 (H-Bond), Tyr212 (H-Bond with Backbone)	Asn60 (H-Bond), Tyr150 (H-Bond with Backbone), Phe208 (H-Bond with Backbone)
STOCKIN-03432	Cys40 (H-Bond), **Phe56 (π-π)**, Ile146 (H-Bond with Backbone), **Ala148 (H-Bond with Backbone)**, Gly207 (Water Bridge H-Bond Network with Backbone), Arg218 (H-Bond)	Gly145 (H-Bond with Backbone), **Ala148 (H-Bond with Backbone)**, Gly211 (H-Bond with Backbone), Phe208 (π-π)
25284644	**Phe208 (π-π)**	Asn60 (H-Bond), **Phe208 (π-π)**, Gly211 (H-Bond with Backbone), Arg218 (H-Bond)
32927247	Gly211 (Water Bridge H-Bond Network with Backbone), **Arg218 (Water Bridge H-Bond Network)**	Asn60 (H-Bond), **Arg218 (H-Bond)**
27371521	Gly145 (Water Bridge H-Bond with Backbone), Tyr150 (H-Bond with Backbone), Tyr212 (π-π and H-Bond with Backbone)	Asn60 (H-Bond), Tyr150, Phe208 (H-Bond with Backbone), Phe208 (π-π)
2801237	Ile59 (Water Bridge H-Bond Network with Backbone), Ile146 (Water Bridge H-Bond Network with Backbone), **Arg218 (Water Bridge H-Bond Network)**	Asn60 (H-Bond), Ala148 (H-Bond with Backbone), Gly211 (H-Bond with Backbone), Phe208 (π-π), **Arg218 (H-Bond)**
5633879	Gly145 (H-Bond with Backbone), **Ala148 (H-Bond with Backbone)**, Tyr212 (H-Bond with Backbone), **Arg218 (H-Bond)**	Asn60 (H-Bond), **Ala148 (H-Bond with Backbone), Arg218 (H-Bond)**,
36994203	**Gly211 (H-Bond with Backbone), Arg218 (H-Bond and** **π** **-cation)**	Asn60 (H-Bond), Gly145 (H-Bond with Backbone), **Gly211 (H-Bond with Backbone), Arg218 (H-Bond)**
16694164	**Gly145 (H-Bond with Backbone)**, Gly207 (Water Bridge H-Bond Network with Backbone), Arg218 (H-Bond)	Asn60 (H-bond), Gly211 (H-Bond with Backbone), **Gly145 (H-Bond with Backbone)**, Ala148 (H-Bond with Backbone)
13477729	Arg218 (Water Bridge Network H-Bond)	Asn60 (H-Bond), Gly145 (H-Bond with Backbone), Gly211 (H-Bond with Backbone)
36657947	Ala148 (Water Bridge H-Bond Network with Backbone), Tyr150 (H-Bond with Backbone), Phe208 (π-π)	Asn60 (H-bond), Gly207 (H-Bond with Backbone)
36716128	Phe63 (**π-π**), Tyr212 (π-π)	Asn60 (H-bond), Gly211, Ala148 (H-Bond with Backbone)
3325122	Val43 (Water Bridge H-Bond Network with Backbone), Val47 (Water Bridge H-Bond Network with Backbone), Leu48 (Water Bridge H-Bond Network with Backbone), **Phe208 (π-π)**	Asn60 (H-Bond), Gly145 (H-Bond with Backbone), Gly211(H-Bond with Backbone), Tyr150, **Phe208 (π-π)**
42888719	Gly145 (Water Bridge H-Bond Network with Backbone), **Phe208 (π-π)**, Tyr212 (H-Bond with Backbone), **Arg218 (H-Bond and Water Bridge H-Bond Network)**	**Phe208 (π-π)**, Gly211 (H-Bond with Backbone), **Arg218 (H-Bond)**
IP6	Asn60 (H-Bond), **Tyr150 (H-Bond with Backbone)**, Pro151 (H-Bond with Backbone), Ser152 (H-Bond with Backbone), **Phe208, (H-Bond with Backbone)**	**Tyr150 (H-Bond with Backbone), Phe208 (H-Bond with Backbone)**, Phe208 (π-π)
31879059	Tyr150 (Water Bridge H-Bond Network with Backbone), Ser210 (Water Bridge H-Bond Network with Backbone), **Arg218 (H-Bond and** **π-cation)**, Trp242 (Water Bridge H-Bond Network with Backbone)	Gly142 (H-Bond with Backbone), **Arg218 (H-Bond)**
31966421	Phe63 (π-π), **Gly145 (H-Bond with Backbone)**, Ala148 (H-Bond with Backbone), **Arg218 (H-Bond)**	Asn60 (H-Bond), **Gly145 (H-Bond with Backbone)**, Phe208 (π-π), **Arg218 (H-Bond)**
7658775	**Gly145 (H-Bond with Backbone), Ala148 (H-Bond with Backbone)**, Tyr150 (H-Bond with Backbone), **Arg218 (H-Bond)**	Gly142 (H-Bond with Backbone), **Gly145 (H-Bond with Backbone), Ala148 (H-Bond with Backbone), Arg218 (H-Bond)**
25665268	Gly145 (Water Bridge H-Bond Network with Backbone), Ala148 (Water Bridge H-Bond Network with Backbone), **Phe208 (H-Bond with Backbone)**, Tyr212 (π-π), Arg218 (π-cation)	Asn60 (H-Bond), Tyr150 (H-Bond with Backbone), **Phe208 (H-Bond with Backbone)**, Phe208 (π-π)
37101119	Ile146 (H-Bond with Backbone), **Gly211 (Water Bridge H-Bond Network with Backbone), Arg218 (H-Bond and Water Bridge H-Bond Network)**	Asn60 (H-Bond), Phe208 (π-π), **Gly211 (H-Bond with Backbone), Arg218 (H-Bond)**

* *The name of the compounds for these given compound ID is mentioned in the Supplementary File*.

### Binding Free Energy Analysis

An MM-GBSA study was performed on the last 10 ns of each dynamic simulation trajectory to estimate the binding association between the AQP3 protein and the selected 20 poses obtained from dynamic simulation. The results of MM-GBSA analysis results are shown in Table 3. Compounds 5633879, IP6, 25284644, 36994203, and 27371521 showed higher binding free energy values (ΔG_Bind) of −74.01, −68.48, −63.87, −62.94, and −61.30 kcal/mol, respectively, among all 20 dynamic poses. Furthermore, a comparison of the molecular docking scores and binding free energies of all top 20 poses revealed that the compound C8C [6-(2-(1H-indol-6-yl)ethyl)-2-aminopyrimidin-4(3H)-one], despite having the highest docking score of −7.55 kcal/mol, shows the lowest binding free energy of −27.28 kcal/mol. Similarly, in the molecular docking some marginally low scored compounds emerged as better inhibitors based on binding free energy. The contributions to the binding free energy by all parameters are shown in Table 3.

**Table 3:** MM-GBSA calculation for selected hit compounds (MM-GBSA was performed on the last 10 ns of the simulation trajectory; mean values are shown with standard error).

Compound ID^*^	**ΔG_Bind**	**ΔG_Coulomb**	**ΔG_Covalent**	**ΔG_Hbond**	**ΔG_Lipo**	**ΔG_vdW**	**ΔG_Packing**	**ΔG_SolGB**
C8C	−27.28 ± 4.11	−8.57 ± 4.56	1.59 ± 0.86	−0.44 ± 0.48	−7.17 ± 0.79	−4.15 ± 0.88	22.98 ± 2.70	−31.51 ± 1.99
STOCKIN-03432	−43.58 ± 8.32	−2.66 ± 8.23	2.29 ± 1.42	−1.27 ± 0.59	−18.90 ± 1.95	−1.20 ± 0.65	17.11 ± 4.65	−38.95 ± 3.44
25284644	−63.87 ± 3.77	−18.97 ± 6.26	1.70 ± 0.78	−0.58 ± 0.22	−22.57 ± 1.08	−1.91 ± 0.42	24.46 ± 6.19	−46.00 ± 1.99
32927247	−43.94 ± 4.30	−13.76 ± 3.28	1.01 ± 0.75	−0.56 ± 0.17	−12.58 ± 1.46	−0.98 ± 0.65	20.27 ± 1.89	−37.35 ± 2.02
27371521	−61.30 ± 3.15	−19.44 ± 3.85	1.67 ± 1.95	−1.80 ± 0.29	−14.91 ± 0.85	−4.24 ± 0.48	21.64 ± 3.27	−44.22 ± 2.04
2801237	−37.65 ± 3.69	−4.73 ± 2.80	0.66 ± 1.57	−0.52 ± 0.19	−10.85 ± 1.44	−0.18 ± 0.22	14.89 ± 2.06	−36.99 ± 2.34
5633879	−74.01 ± 3.21	−25.67 ± 2.49	2.03 ± 0.76	−2.57 ± 0.17	−24.31 ± 1.31	−2.81 ± 0.60	30.14 ± 1.81	−50.82 ± 1.68
36994203	−62.94 ± 3.98	−21.86 ± 4.04	−0.22 ± 0.87	−1.74 ± 0.23	−16.12 ± 1.85	−1.30 ± 0.46	15.45 ± 1.54	−37.16 ± 1.97
16694164	−57.61 ± 4.61	−12.84 ± 4.46	−1.49 ± 2.18	−1.53 ± 0.23	−17.02 ± 2.37	−3.06 ± 0.48	21.50 ± 3.16	−43.17 ± 2.78
13477729	−51.22 ± 4.96	−16.58 ± 3.27	2.32 ± 0.83	−1.11 ± 0.29	−15.49 ± 1.60	−2.23 ± 0.43	25.84 ± 2.72	−43.97 ± 2.09
36657947	−46.58 ± 2.81	−16.22 ± 3.04	1.60 ± 1.09	−0.51 ± 0.06	−15.32 ± 0.90	0.00 ± 0.01	23.40 ± 1.98	−39.53 ± 2.52
36716128	−53.39 ± 2.70	−7.03 ± 2.27	1.09 ± 0.68	−1.03 ± 0.17	−13.10 ± 0.72	−2.71 ± 0.58	17.35 ± 1.71	−47.98 ± 1.76
3325122	−49.38 ± 6.36	−11.88 ± 6.41	2.43 ± 1.87	−1.03 ± 0.46	−17.42 ± 1.31	−1.43 ± 0.77	16.48 ± 4.63	−36.53 ± 2.45
42888719	−39.14 ± 3.34	−12.85 ± 4.11	0.89 ± 0.72	−0.30 ± 0.25	−12.81 ± 0.86	−0.13 ± 0.17	20.03 ± 2.43	−33.97 ± 1.89
IP6	−68.48 ± 6.00	−32.19 ± 12.56	2.26 ± 1.82	−1.21 ± 0.48	−22.35 ± 1.53	−5.07 ± 1.46	33.41 ± 10.84	−43.33 ± 3.68
31879059	−60.16 ± 4.30	−13.68 ± 3.94	1.36 ± 1.66	−0.37 ± 0.21	−23.17 ± 1.33	−3.32 ± 0.63	20.42 ± 3.30	−41.40 ± 2.34
31966421	−56.35 ± 5.39	−20.05 ± 4.84	1.12 ± 1.12	−1.80 ± 0.42	−15.22 ± 0.81	−2.90 ± 0.64	24.30 ± 2.18	−41.80 ± 2.59
7658775	−57.55 ± 3.89	−17.07 ± 4.14	1.91 ± 0.81	−1.47 ± 0.20	−20.30 ± 1.32	−2.31 ± 0.42	30.92 ± 2.43	−49.23 ± 1.88
25665268	−33.80 ± 7.86	−15.01 ± 6.94	2.48 ± 1.58	−1.23 ± 0.48	−9.25 ± 2.06	−0.39 ± 0.58	18.20 ± 4.60	−28.59 ± 5.57
37101119	−47.54 ± 5.20	−10.53 ± 4.71	−0.64 ± 2.32	−0.66 ± 0.68	−14.42 ± 1.60	−2.27 ± 0.57	21.20 ± 2.28	−40.21 ± 2.52

* *The name of the compounds for these given compound ID is mentioned in the Supplementary File*.

Among all the binding free energies measured, the compound 5633879 [1-(4-methoxyphenoxy)-3-((4-methoxyphenyl)amino)propan-2-ol] had the highest ΔG_Bind (−74.01 kcal/mol) with a docking score of −7.152 kcal/mol, forming H-bond with Ala148, Tyr212, and Arg218 residues in a dynamic trajectory that was similar to the interaction observed in the docking pose (Arg60, Ala148, Arg218). In the docked pose, the oxygen atoms of the –OCH_3_ and –OH groups formed H-bonds at a distance of 2.02 and 2.24 Å, respectively, with the –NH- group of Asn60 and the guanidino group of Arg218. Likewise, H-bond network with the backbone carbonyl of Ala148 was observed with the –NH- and –OH groups of the linker region of the compound. Additionally, we observed some additional interactions in dynamic poses, such as H-bonds with the backbone atoms of the Tyr212 residues. The interaction fractions of the compound 5633879 showed it in the vicinity of Phe63, Ty150, Phe208, and Tyr212, imparting hydrophobic interactions during simulation (Figures S36b,c).

Similarly, compound 31966421 [3-(1H-benzo[d]imidazol-2-yl)-N-(2-methyl-3-(1H-pyrazol-1-yl)propyl)propanamide] had an intermediary ΔG_Bind (−56.35 kcal/mol) with a docking score of −6.747 kcal/mol. It forms H-bonds with Asn60, Gly145, and Arg218, and π-π interactions with Phe208 residues during docking, while in dynamic simulation the compound retains H-bonds with Asn60, and Arg218, as well as forming new H-bond and π-π interactions with the Ile146, Gly211, Tyr212, and Phe56 residues, respectively. In the docking pose, the –OH group of the linker and the ring nitrogen of imidazole form H-bonds with Arg218 and Asn60 at a distance of 2.19 and 1.96 Å, respectively. The –NH- group of the benzimidazole group also forms H-bond with the backbone carbonyl of Gly145. In dynamic trajectories, the residues Asn60 and Arg218 retain the H-bond interactions for more than 30% of the simulation time and the imidazole group engages into a π-π interaction with Phe56.

Compound C8C, which has been identified as the top scorer in molecular docking, displays the lowest binding free energy among all the compounds. In the docking pose, the compound has H-bonds with the carbonyls of Asn60, Tyr150, and Phe208 with distances of 2.07, 2.06, and 2.0 Å, respectively; whereas in the dynamic trajectory the compound failed to display any H-bonds for 20% of the simulation while compound showed a π-π interaction with Phe63. Despite all the individual components that contribute to the binding free energy in the MM-GBSA analysis, the insignificant contribution of the ΔG_Lipo resulting from higher ligand strain energy could result in lower binding free energy.

It is notable to mention here the role of water-bridge with compounds and binding amino acid residues. Analyzing all the dynamic poses of compounds with their interactions in binding site residues as shown in Tables 2, 3, showed that, most of the compounds with good binding free energy exhibited water-bridge interaction (Table 3). Although Figures S30d–S49d showed H-Bond interaction formed throughout the dynamic simulations, Figure S54 shows protein-ligand interaction for some selected compounds (last frames) depicting the water-bridge mediated H-Bond interaction. These compounds can restrict the flow of solute/solvents by bridging with the water molecules and further reinforce the binding interactions.

It is worth noting that this mechanism of AQP3 inhibition is very different from what observed by Casini et al. the case of gold-based compounds (De Almeida et al., 2017). In this latter case, binding of the Au(III) complex to the Cys40 in the AQP3 channel induces protein's conformational changes leading to restriction of the pore. Of note, the same authors reported on the importance of importance of non-coordinative adducts in modulating the AQP3 inhibition properties of the investigated Au(III) compounds (Wenzel et al., 2019).

Thus, based on binding free energy values, the order of top 10 compounds is 5633879 > IP6 > 25284644 > 36994203 > 27371521 > 31879059 > 16694164 > 7658775 > 31966421 > 36716128.

### Exploration of the Ligand-Unbinding Pathways

**Figure 6:**
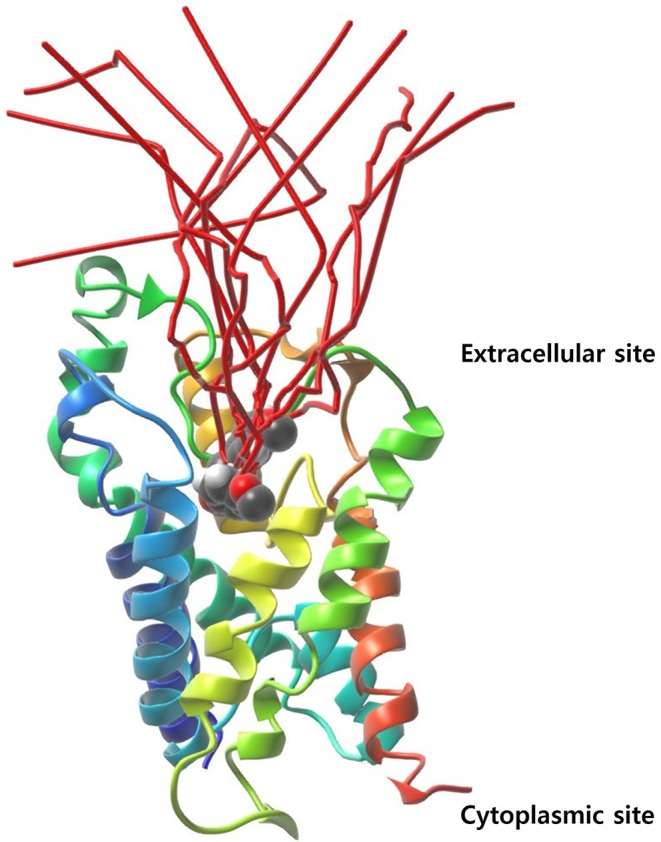
The ligand-unbinding path (in red color). The protein is represented with a ribbon and the ligand in VdW sphere.

The initial protein-ligand complex and the active atoms on compound 5633879 [1-(4-methoxyphenoxy)-3-((4-methoxyphenyl) amino) propan-2-ol] are shown in Figure 4, illustrating no direction-bias sampling-domain. From all 10 runs, we found that the ligand-unbinding process followed a common pathway (Figure 6 and Supplementary Movie 01). The statistics regarding ligand-unbinding path are shown in Table S3. The AQP3 membrane protein has two sites, one being extracellular and one being cytoplasmic. To permeate the cell, solute molecules must follow the extracellular site. Our ligand-unbinding pathway search revealed that despite no direction-bias sampling, ligand unbinding and exit from the protein followed the extracellular site.

## Conclusions

The diverse roles of AQPs have been well-established in physiology and their involvement in prognosis of a variety of disease states, which necessitate the discovery of selective modulators or inhibitors as therapeutic agents. AQP3 is widely distributed in epithelial cells of the kidney, airway, and skin, plays a role in mucosal secretions, water reabsorption, skin hydration, and regulation of cell volume. Nevertheless, an earlier study documented the aberrant expression of AQP3 in melanoma cells, indicating that a new therapeutic treatment would result in its inhibition. In this study, we performed a virtual screening to identify novel hits for inhibitors of the AQP3 target protein. A total of 20 hits with good binding affinity were obtained from a combination of pharmacophore and docking-based screening strategies. The physicochemical properties of the selected compounds comply with skin permeability properties. The hit compounds obtained bind to key amino acid residues (Phe63, Tyr212, and Arg218) to inhibit the activity of the AQP3 protein. The molecular dynamics and MM-GBSA analysis revealed that the compound 5633879 [1-(4-methoxyphenoxy)-3-((4-methoxyphenyl) amino) propan-2-ol] has good free energy of binding. Since the AQP3 is a channel protein embedded into the basolateral layer of the skin membrane, it has two relevant sites: an extracellular site and a cytoplasmic site. In order to module its function the inhibitor must approach the AQP3 protein via the extracellular site, which led us to further explore the ligand-unbinding pathway of the bound protein-ligand complex system. The ligand-unbinding pathway also revealed that the inhibitor approached the binding site through the extracellular site. The hit compounds obtained from the present study promise good docking scores and binding free energy for the AQP3 protein. The mechanism of action of these hits as inhibitors of AQP3 however remains elusive compared to metal based compounds. The permeation mechanism of water and glycerol was recently, elucidated by Wragg et al. (2019) through enhanced sampling method i.e., metadynamics simulation. A similar approach can be applied on the identified hits to understand its mechanism of inhibition at the atomic level and this will further enable us to optimize the hit and biological screening.

## Data Availability Statement

All datasets generated for this study are included in the article/Supplementary Material.

## Author Contributions

DY conceived and designed the project and collected data from the literature and databases. SK and DY performed the experiments, analyzed the data, and wrote the manuscript. E-HC, M-HK, and SC provided the facility. All authors contributed to the interpretation and discussion of the results, read and approved the final version of the manuscript.

### Conflict of Interest

The authors declare that the research was conducted in the absence of any commercial or financial relationships that could be construed as a potential conflict of interest.
