# Supplementary material for: Computational Modeling on Aquaporin-3 as Skin Cancer Target: A Virtual Screening Study
Source: Front Chem. 2020 Apr 15;8:250. doi: 10.3389/fchem.2020.00250 (PMC7175779; doi:10.3389/fchem.2020.00250)
Supplement: Supplementary file 1 [file Data_Sheet_1.PDF]

Computational Modeling on Aquaporin-3 as Skin Cancer Target: A Virtual Screening

Dharmendra Kumar Yadav[a, $*], Surendra Kumar[a, $], Eun-Ha Choi[b], Sandeep Chaudhary[c], Mi-Hyun Kim[a,*]

[a]Gachon Institute of Pharmaceutical Science & Department of Pharmacy, College of Pharmacy, Gachon University, Incheon, 21936, South Korea.
[b]Plasma Bioscience Research Center/PDP Research Center, Kwangwoon University, Nowon-Gu, Seoul, 139-791, South Korea
[c]Laboratory of Organic & Medicinal Chemistry, Department of Chemistry, Malaviya National Institute of Technology, Jawaharlal Nehru Marg, Jaipur
302017 (India)
Email:dharmendra30oct@gmail.com, *kmh0515@gachon.ac.kr*

*corresponding author

Dr.Dharmendra Kumar Yadav, Ph.D
Assistant Professor
Office: +82-32-820-4947
Email: dharmendra30oct@gmail.com

$These authors contributed equally to this work and share first authorship

Figure S1. The AQP3 protein structure with identified Site_1 and Site_2 (Circled region) from SiteMap tool of Schrodinger suite. The triad key amino acid residues (Phe63, Tyr212, Arg218) shown in ball and stick form.

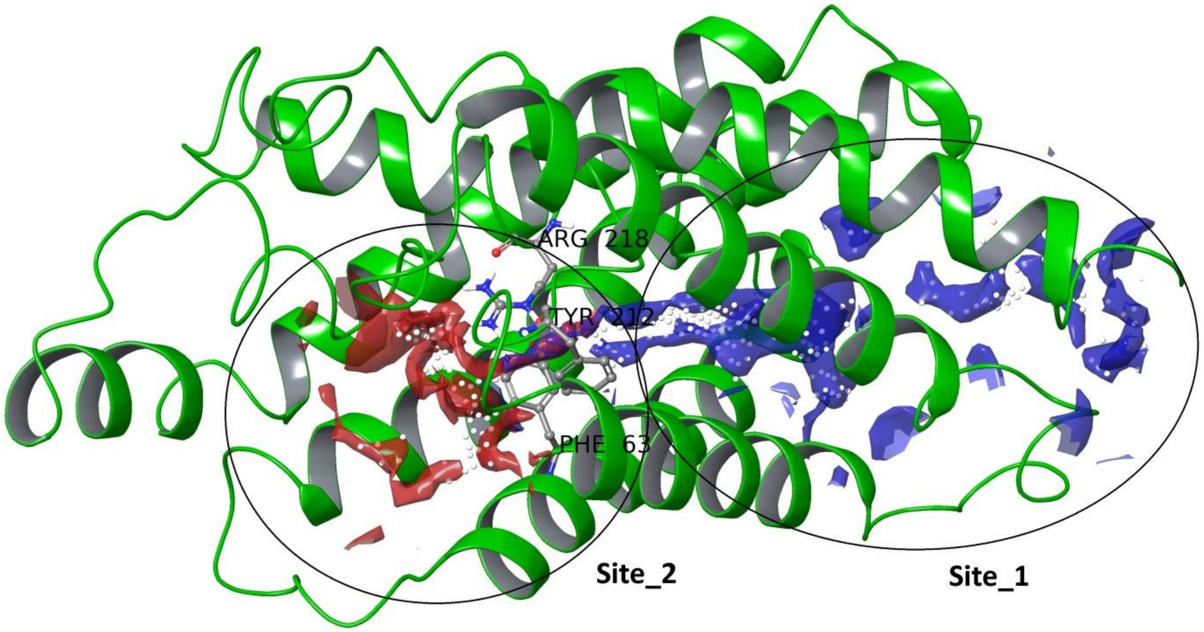

Figure S2. The representative Lipid-protein-ligand system built for molecular dynamic simulation. The lipids proteins and ligands are represented in green colour, cartoon and yellow colour with vdW sphere respectively.

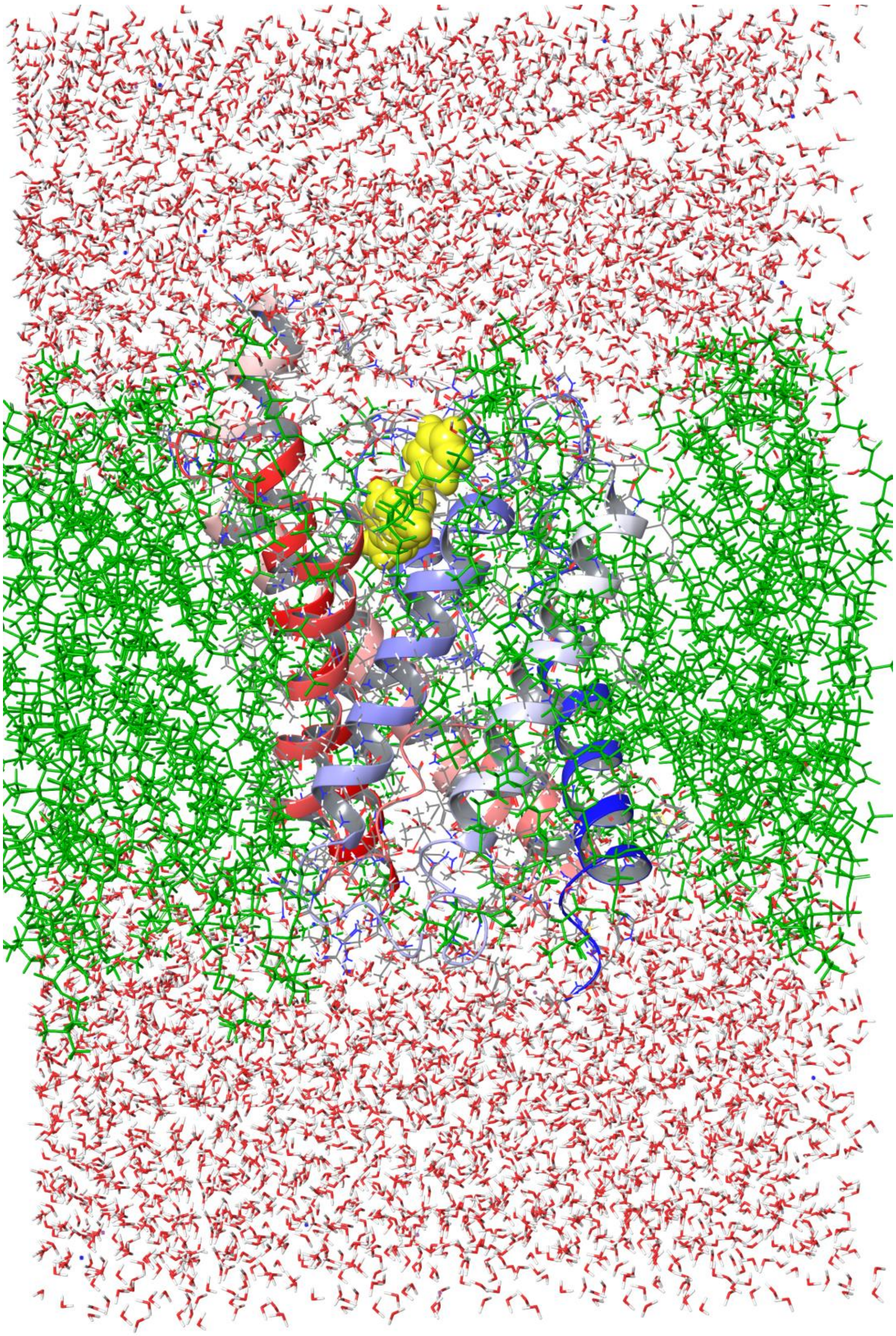

Figure S3. The physicochemical properties based filter includes **(A)** Molecular Weight (Selected molecular weight ranges from 20 to 300); **(B)** Aqueous solubility (LogS) (Selected LogS ranges from -9.0 to 1.0); **(C)** Skin Permeability (LogKp) (Selected LogKp ranges from -8.0 to 1.0; **(D)** Maximal Transdermal Transport Rate (Jm) (Selected molecules with Jm <10; **(E)** Number of Reactive Functional Groups (Selected range incudes 0 to 2).

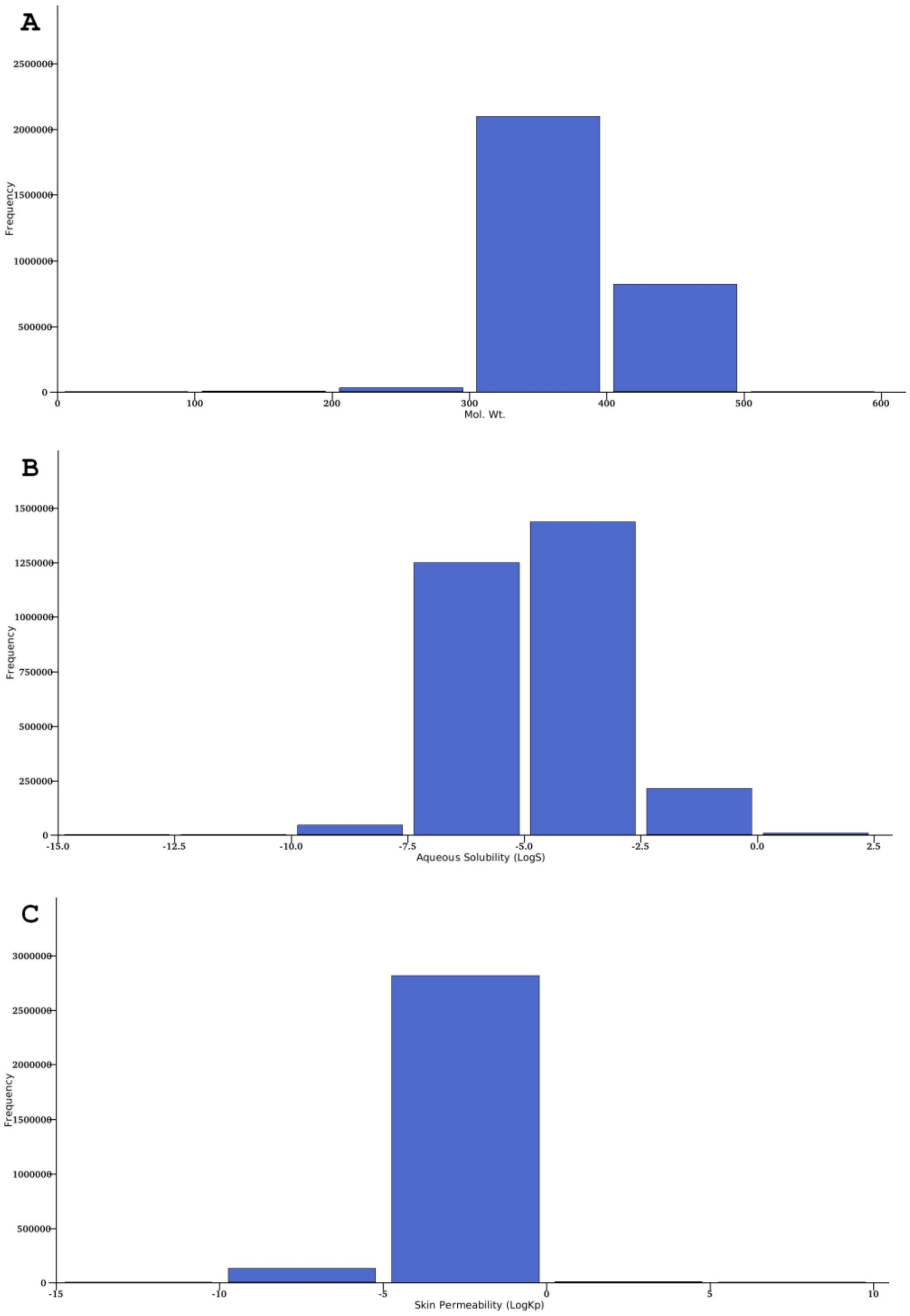

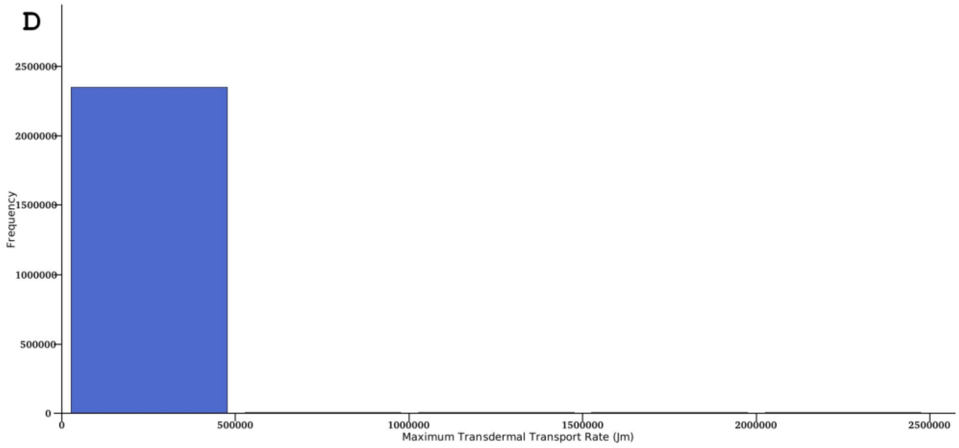
D
Frequency
0
500000
1000000
1500000
2000000
2500000
Maximum Transdermal Transport Rate (Jm)

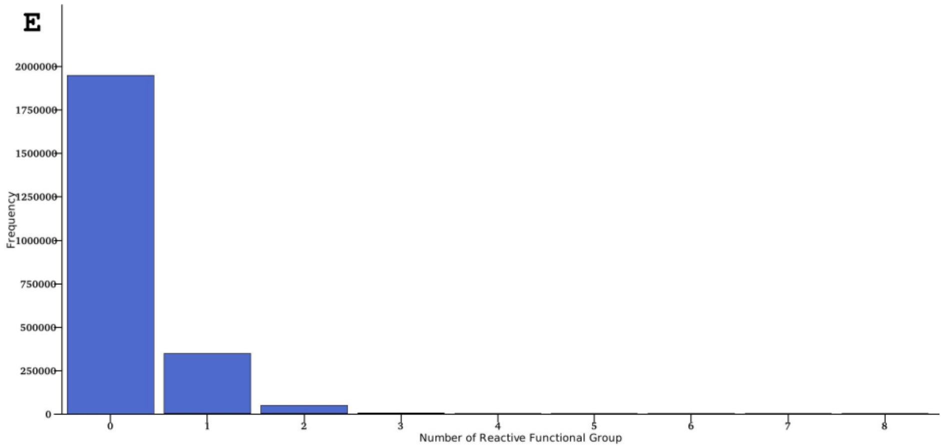
E
Frequency
0
250000
500000
750000
1000000
1250000
1500000
1750000
2000000
0
1
2
3
4
5
6
7
8
Number of Reactive Functional Group

Figure S4. The pharmacophoric elements found in the top 20 hits. The legends for each colour is shown below of these structures.

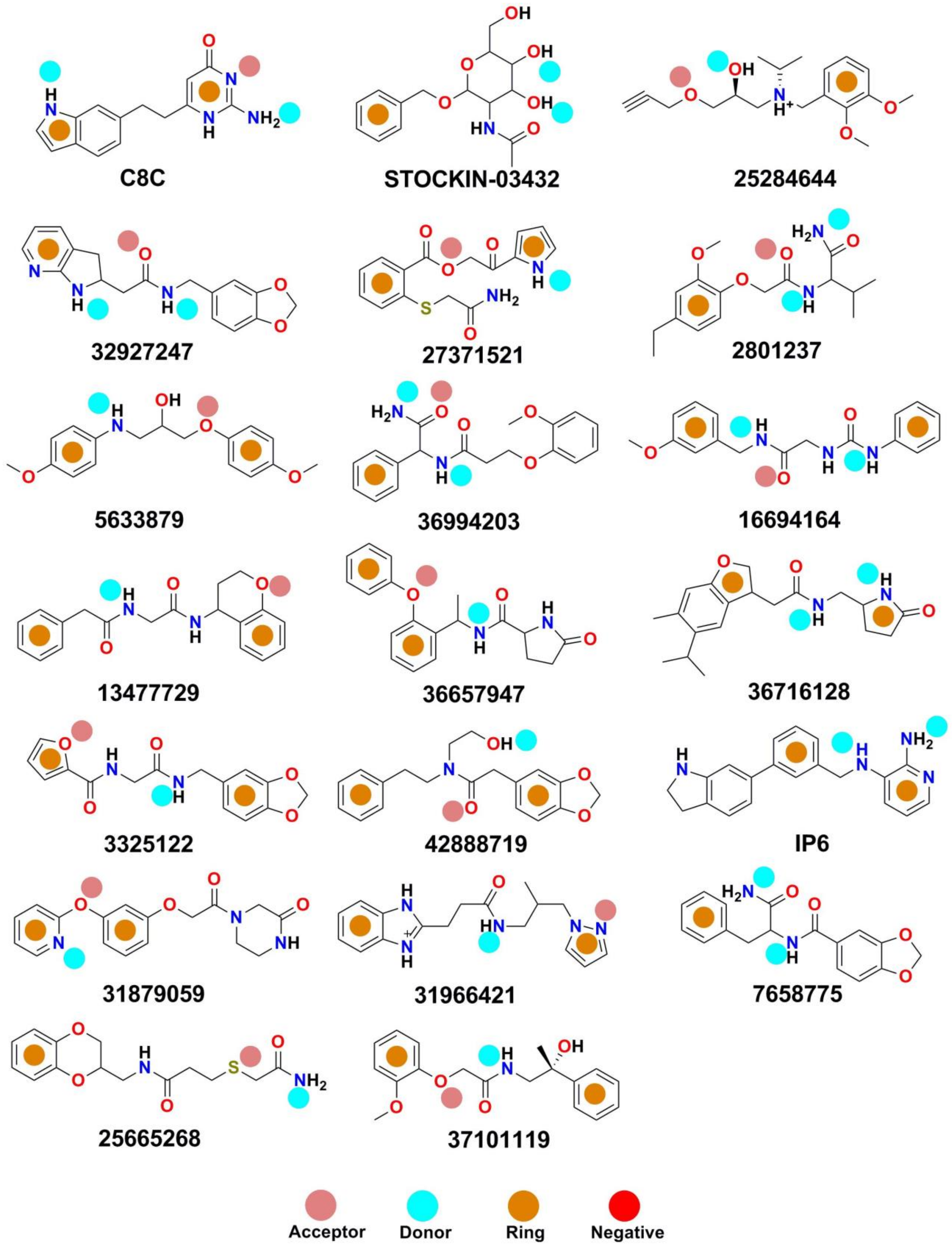

Table S1. The top 20 hit compound selected after docking simulation (XP mode). The docking score ranges from -7.55 to -6.722.

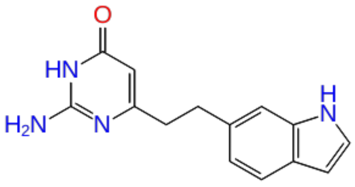	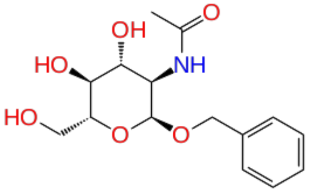	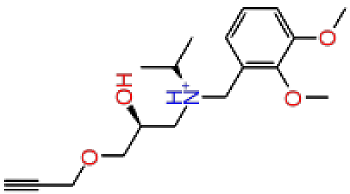	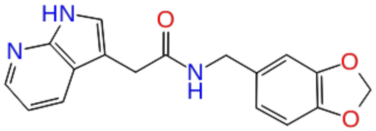	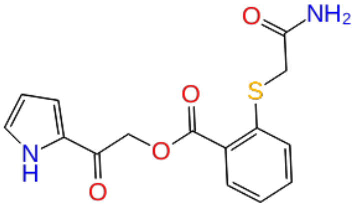
Title: **C8C (6-(2-(1H-indol-6-yl)ethyl)-2-amino pyrimidin-4(3H)-one)**<br>Docking Score: **-7.55**	Title: **STOCKIN-03432 (N-(2-(benzyloxy)-4,5-dihydroxy-6-(hydroxymethyl)tetrahydro -2H-pyran-3-yl)acetamide)**<br>Docking Score: **-7.497**	Title: **25284644 (N-(2,3-dimethoxybenzyl)-2-hydroxy-N-isopropyl-3-(prop-2-yn-1-yloxy) propan-1-aminium)**<br>Docking Score: **-7.353**	Title: **32927247 (N-(benzo[d][1,3] dioxol-5-ylmethyl)-2-(1H-pyrrolo[2,3-b]pyridin-3-yl) acetamide)**<br>Docking Score: **-7.353**	Title: **27371521 (2-oxo-2-(1H-pyrrol-2-yl)ethyl 2-((2-amino-2-oxoethyl)thio) benzoate)**<br>Docking Score: **-7.303**
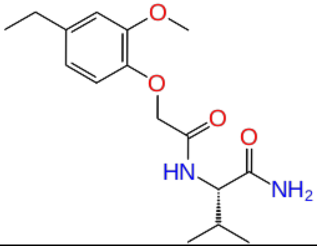	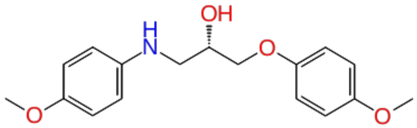	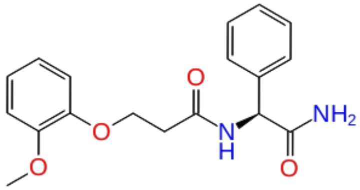	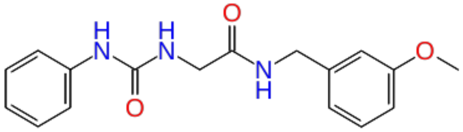	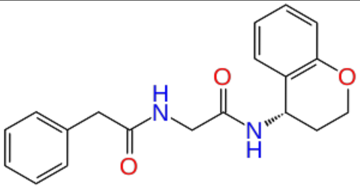
Title: **2801237 (2-(2-(4-ethyl-2-methoxy phenoxy)acetamido)-3-methylbutanamide)**<br>Docking Score: **-7.285**	Title: **5633879 (1-(4-methoxy phenoxy)-3-((4-methoxy phenyl)amino)propan-2-ol)**<br>Docking Score: **-7.152**	Title: **36994203 (N-(2-amino-2-oxo-1-phenyl ethyl)-3-(2-methoxy phenoxy)propanamide)**<br>Docking Score: **-7.10**	Title: **16694164 (N-(3-methoxy benzyl)-2-(3-phenylureido) acetamide)**<br>Docking Score: **-7.018**	Title: **13477729 (N-(chroman-4-yl)-2-(2-phenylacetamido)acetami de)**<br>Docking Score: **-6.995**
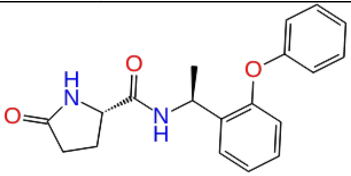	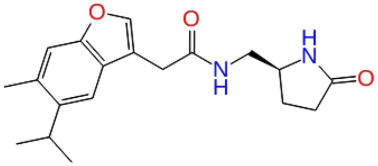	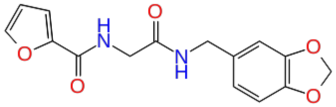	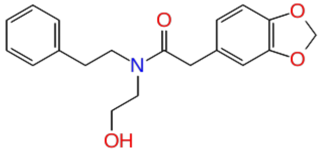	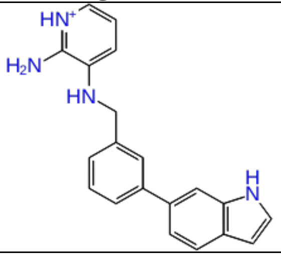

Title: **36657947 (5-oxo-N-(1-(2-phenoxyphenyl) ethyl) pyrrolidine-2-carboxamide)**<br>Docking Score: **-6.949**	Title: **36716128 (2-(5-isopropyl-6-methyl benzofuran-3-yl)-N-((5-oxopyrrolidin-2-yl)methyl) acetamide)**<br>Docking Score: **-6.929**	Title: **3325122 (N-(2-((benzo[d][1,3]dioxol-5-ylmethyl)amino)-2-oxoethyl)furan-2-carboxamide)**<br>Docking Score: **-6.914**	Title: **42888719 (2-(benzo[d][1,3] dioxol-5-yl)-N-(2-hydroxyethyl)-N-phenethyl acetamide)**<br>Docking Score: **-6.801**	Title: **IP6 (3-((3-(1H-indol-6-yl)benzyl)amino)-2-aminopyridin-1-ium)**<br>Docking Score: **-6.778**
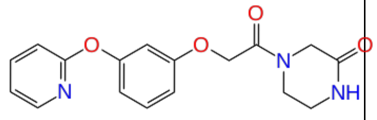	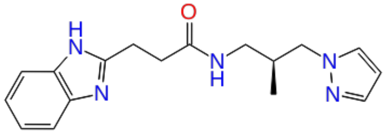	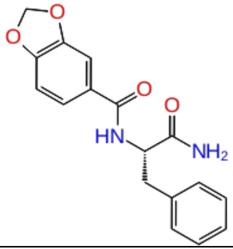	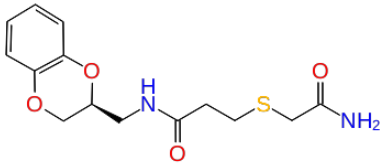	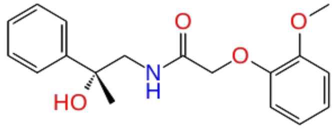
Title: **31879059 (4-(2-(3-(pyridin-2-yloxy) phenoxy)acetyl)piperazi n-2-one)**<br>Docking Score: **-6.76**	Title: **31966421 (3-(1H-benzo [d]imidazol-2-yl)-N-(2-methyl-3-(1H-pyrazol-1-yl)propyl)propanamide)**<br>Docking Score: **-6.747**	Title: **7658775 (N-(1-amino-1-oxo-3-phenylpropan-2-yl)benzo [d][1,3]dioxole-5-carboxamide)**<br>Docking Score: **-6.746**	Title: **25665268 (3-((2-amino-2-oxoethyl)thio)-N-((2,3-dihydro benzo[b][1,4]dioxin-2-yl)methyl)propanamide)**<br>Docking Score: **-6.737**	Title: **37101119 (N-(2-hydroxy-2-phenylpropyl)-2-(2-methoxyphenoxy) acetamide)**<br>Docking Score: **-6.722**

Table S2. Docking Score, and amino acid residues in the binding site within 3Å for top 20 hits.

Compound ID	Docking Score	Amino Acid Residues in the binding site within 3Å	Amino Acid Residues involved in interactions
**C8C**	-7.55	Val43, Ile59, Asn60, Phe63, Tyr150, Pro151, Ser152, Gly207, Phe208, Asn209, Tyr212	Asn60 (H-Bond), Tyr150 (H-Bond with Backbone), Phe208 (H-Bond with Backbone)
**STOCKIN-03432**	-7.497	Val43, Phe56, Ile59, Asn60, Phe63, Gly145, Ile146, Ala148, Thr149, Phe208, Gly211, Tyr212, Arg218	Gly145 (H-Bond with Backbone), Ala148 (H-Bond with Backbone), Gly211 (H-Bond with Backbone), Phe208 (π-π)
**25284644**	-7.353	Cys40, Val43, Phe56, Ile59, Asn60, Phe63, Gly145, Phe147, Ala148, Thr149, Gly203, Thr204, Gly207, Phe208, Gly211, Tyr212, Arg218	Asn60 (H-Bond), Phe208 (π-π), Gly211 (H-Bond with Backbone), Arg218 (H-Bond)
**32927247**	-7.353	Val43, Asn60, Phe63, Asn141, Gly142, Gly145, Ala148, Thr149, Tyr150, Gly207, Gly211, Tyr212, Arg218	Asn60 (H-Bond), Arg218 (H-Bond)
**27371521**	-7.303	Val43, Ile59, Asn60, Phe63, Tyr150, Ser152, Gly207, Phe208, Asn209, Tyr212, Arg218	Asn60 (H-Bond), Tyr150, Phe208 (H-Bond with Backbone), Phe208 (π-π)
**2801237**	-7.285	Val43, Phe56, Ile59, Asn60, Gly145, Ala148, Thr149, Gly203, Thr204, Gly207, Phe208, Gly211, Tyr212, Arg218	Asn60 (H-Bond), Ala148 (H-Bond with Backbone), Gly211 (H-Bond with Backbone), Phe208 (π-π), Arg218 (H-Bond)
**5633879**	-7.152	Val43, Phe56, Asn60, Asn141, Gly142, Gly145, Ala148, Thr149, Tyr150, Gly207, Gly211, Tyr212, Arg218	Asn60 (H-Bond), Ala148 (H-Bond with Backbone), Arg218 (H-Bond),
**36994203**	-7.100	Val43, Phe56, Ile59, Asn60, Phe63, Gly145, Ile146, Phe147, Ala148, Thr149, Tyr150, Gly203, Thr204, Gly207, Phe208, Gly211, Tyr212, Arg218	Asn60 (H-Bond), Gly145 (H-Bond with Backbone), Gly211 (H-Bond with Backbone), Phe208 (π-π), Arg218 (H-Bond)
**16694164**	-7.018	Val43, Phe56, Ile59, Asn141, Gly142, Gly145, Ala148, Thr149, Tyr150, Gly207, Phe208, Gly211, Arg218	Asn60 (H-bond), Gly211 (H-Bond with Backbone), Gly145 (H-Bond with Backbone), Ala148 (H-Bond with Backbone)
**13477729**	-6.995	Val43, Ile59, Asn60, Asn141, Gly142, Gly145, Ala148, Thr149, Tyr150, Pro151, Gly207, Gly211, Tyr212	Asn60 (H-Bond), Gly145 (H-Bond with Backbone), Gly211 (H-Bond with Backbone)
**36657947**	-6.949	Val43, Phe56, Ile59, Asn60, Phe63, Gly145, Ala148, Thr149, Tyr150, Gly207, Phe208, Gly211, Tyr212,	Asn60 (H-bond), Gly207 (H-Bond with Backbone)

		Arg218	
**36716128**	-6.929	Val43, Val46, Gly55, Phe56, Ile59, Asn60, Gly145, Ala148, Thr149, Tyr150, Gly207, Gly211, Tyr212, Arg218	Asn60 (H-bond), Gly211, Ala148 (H-Bond with Backbone)
**3325122**	-6.914	Val43, Phe56, Ile59, Asn60, Gly142, Gly145, Ala148, Thr149, Tyr150, Gly207, Phe208, Gly211, Tyr212	Asn60 (H-Bond), Gly145 (H-Bond with Backbone), Gly211(H-Bond with Backbone), Tyr150, Phe208 (π-π)
**42888719**	-6.801	Val43, Phe56, Ile59, Asn60, Phe63, Gly145, Ala148, Thr149, Tyr150, Gly207, Phe208, Gly211, Tyr212 , Arg218	Phe208 (π-π), Gly211 (H-Bond with Backbone), Arg218 (H-Bond)
**IP6**	-6.778	Val43, Phe56, Ile59, Asn60, Gly145, Thr149, Tyr150, Gly207, Phe208, Gly211, Tyr212	Tyr150 (H-Bond with Backbone), Phe208 (H-Bond with Backbone), Phe208 (π-π)
**31879059**	-6.760	Val43, Ile59, Asn60, Asn141, Gly142, Gly145, Phe147, Ala148, Tyr150, Gly207, Gly211, Arg218	Gly142 (H-Bond with Backbone), Arg218 (H-Bond)
**31966421**	-6.747	Val43, Phe56, Ile59, Asn60, Asn141, Gly142, Gly145, Ala148, Thr149, Tyr150, Gly207, Phe208, Gly211, Tyr212, Arg218	Asn60 (H-Bond), Gly145 (H-Bond with Backbone), Phe208 (π-π), Arg218 (H-Bond)
**7658775**	-6.746	Val43, Phe56, Ile59, Asn60, Phe63, Asn141, Gly145, Tyr150, Gly207, Phe208, Gly211, Tyr212, Arg218	Gly142 (H-Bond with Backbone), Gly145 (H-Bond with Backbone), Ala148 (H-Bond with Backbone), Arg218 (H-Bond)
**25665268**	-6.737	Val43, Phe56, Ile59, Asn60, Phe63, Tyr150, Gly207, Phe208, Asn209, Tyr212, Arg218	Asn60 (H-Bond), Tyr150 (H-Bond with Backbone), Phe208 (H-Bond with Backbone), Phe208 (π-π)
**37101119**	-6.722	Val43, Phe56, Ile59, Asn60, Phe63, Gly145, Ile146, Ala148, Thr149, Tyr150, Gly203, Thr204, Gly207, Phe208, Tyr212, Gly211, Arg218	Asn60 (H-Bond), Phe208 (π-π), Gly211 (H-Bond with Backbone), Arg218 (H-Bond)

Table S3. Ligand unbinding path result for compound (5633879).

Path id	No. of States	MinE (kcal/mol)	MaxE (kcal/mol)	Saddle (kcal/mol)	Barrier (kcal/mol)	Time(s)	First (kcal/mol)	Last (kcal/mol)	Remarks
0	71	-3408.05	-212.734	3195.32	0	72.904	-212.734	-3408.05	Run_1
1	82	-3385.79	-212.734	3173.06	0	72.164	-212.734	-3382.9	Run_2
2	107	-3483.71	-212.734	3270.98	0	97.86	-212.734	-3483.71	Run_3
3	72	-3385.29	-212.734	3172.56	0	78.688	-212.734	-3385.29	Run_4
4	81	-3442.69	-212.734	3229.96	0	69.46	-212.734	-3442.69	Run_5
5	76	-3389.39	-212.734	3176.66	0	117.076	-212.734	-3389.39	Run_6
6	86	-3425.77	-212.734	3213.04	0	108.872	-212.734	-3425.77	Run_7
7	71	-3186.55	-212.734	2973.81	0	68.092	-212.734	-3186.55	Run_8
8	66	-3397.96	-212.734	3185.23	0	67.8	-212.734	-3397.96	Run_9
9	68	-3342.22	-212.734	3129.49	0	68.828	-212.734	-3342.22	Run_10

No. of states: number conformations in the path; **MinE (kcal/mol):** Minimum energy of the path conformation; **MaxE (kcal/mol):** Maximum energy of the path conformation; **Saddle (kcal/mol):** Difference between MaxE and MinE; **Barrier (kcal/mol):** Difference between MaxE and First; **Time (s):** Time elapsed for searching this path; **First (kcal/mol):** Energy of the first conformation in the path; **Last (kcal/mol):** Energy of the last conformation in the path.

Figure S5. Binding site residues interactions of docked poses with AQP-3 protein. (Amino acid residues displayed interactions within 3Å are shown).

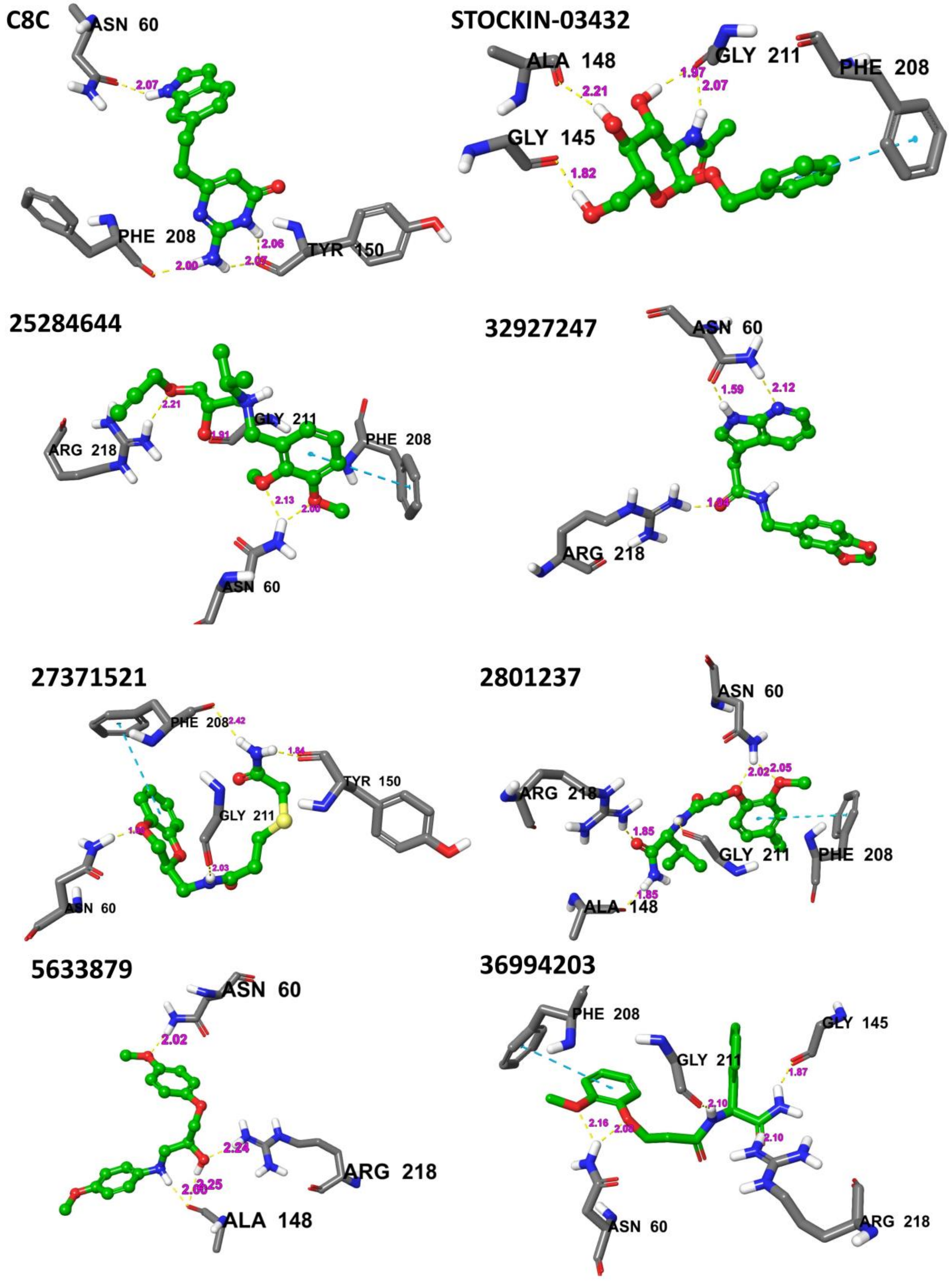

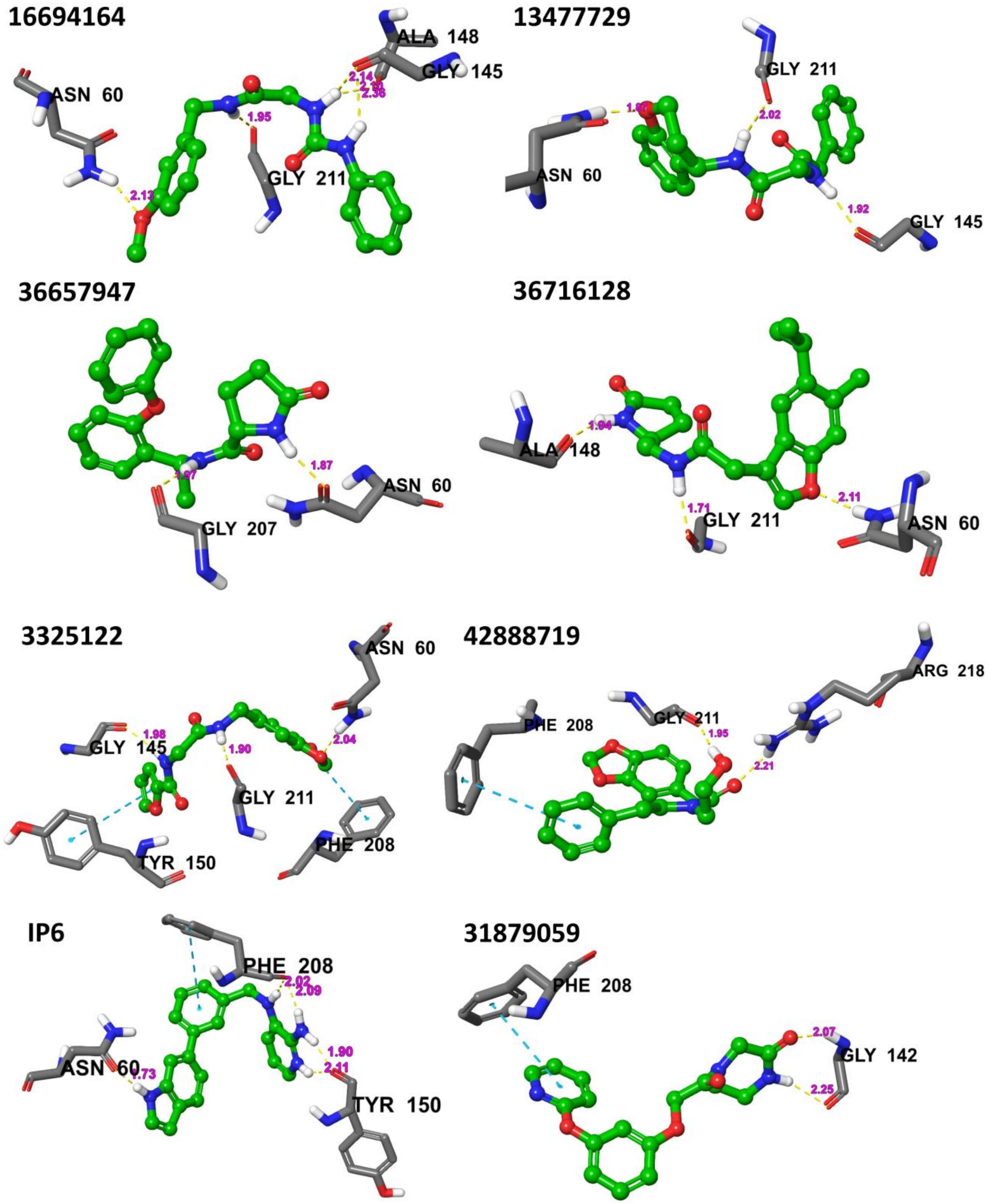

16694164
ASN 60
GLY 211
ALA 148
GLY 145
13477729
GLY 211
ASN 60
GLY 145
36657947
GLY 207
ASN 60
36716128
ALA 148
GLY 211
ASN 60
3325122
ASN 60
GLY 145
GLY 211
TYR 150
PHE 208
42888719
PHE 208
GLY 211
ARG 218
IP6
PHE 208
ASN 60
TYR 150
31879059
PHE 208
GLY 142

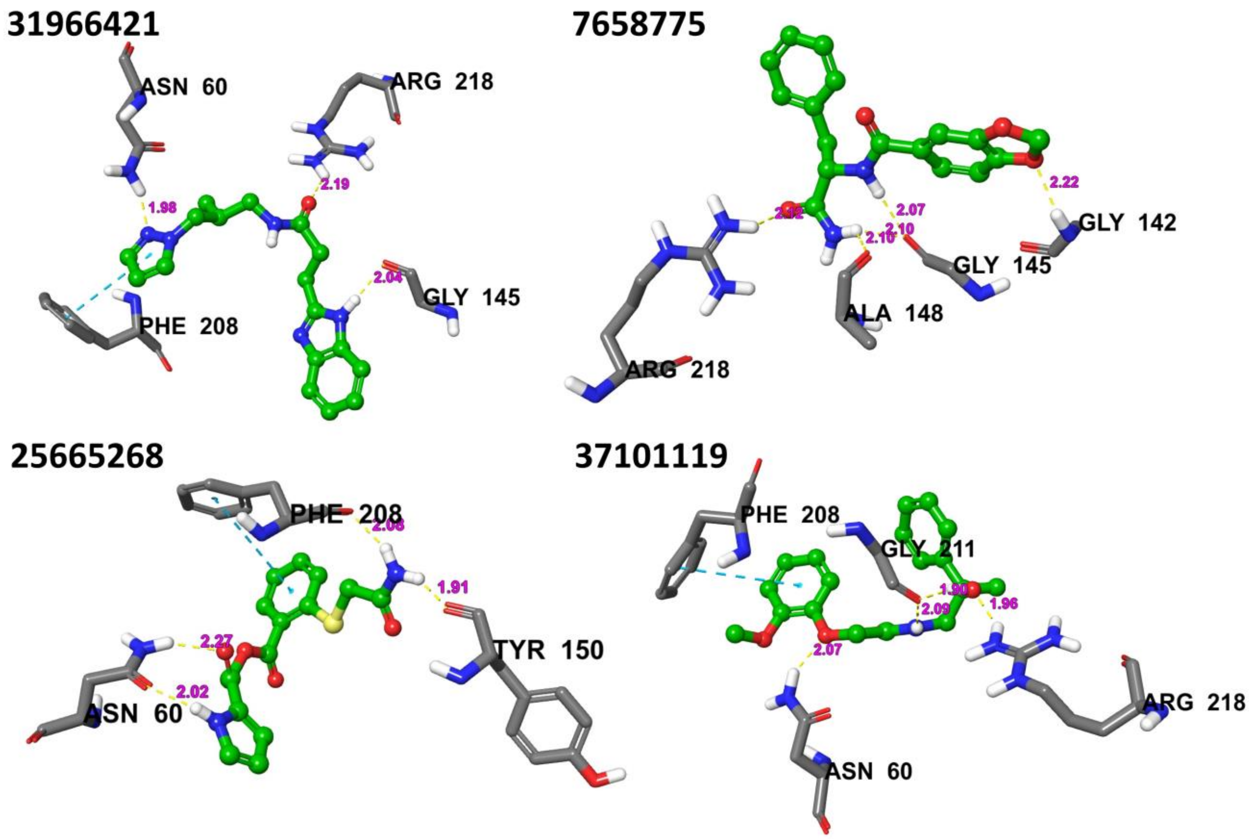

31966421
ASN 60
ARG 218
1.98
2.19
2.04
GLY 145
PHE 208
7658775
2.07
2.10
2.22
GLY 142
GLY 145
ALA 148
ARG 218
25665268
PHE 208
1.91
2.27
2.02
TYR 150
ASN 60
37101119
PHE 208
GLY 211
2.09
1.96
2.07
ARG 218
ASN 60

Figure S6. Total energy (E) (kcal/mol); Potential energy (E_P) (kcal/mol); Pressure (P) (in bar), Temperature (T) (in K), and Volume (V) (in Å ^{3}) during the 100ns of molecular simulation for C8C in complex with AQP-3.

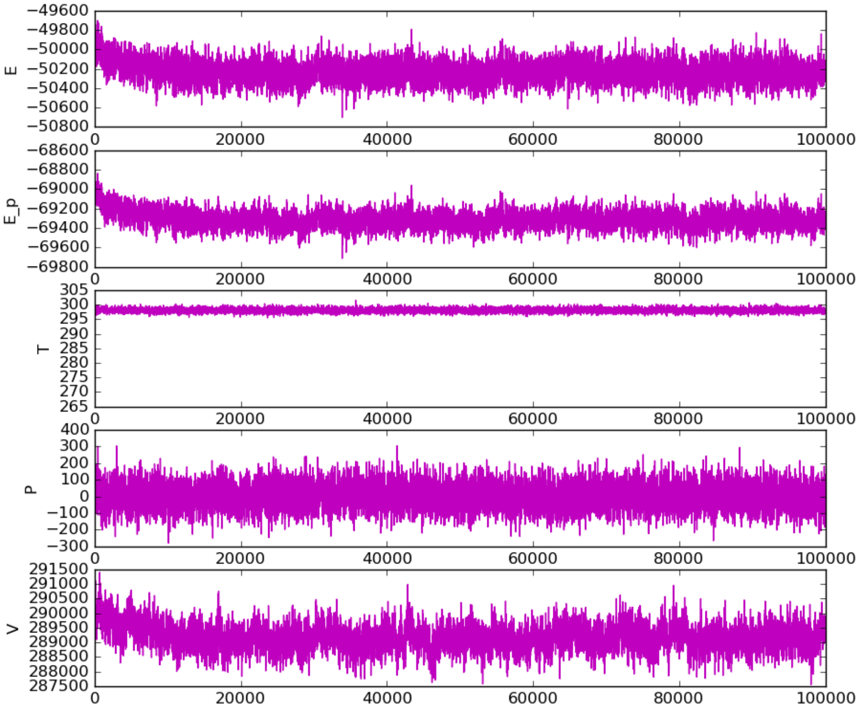

Figure S7. Total energy (E) (kcal/mol); Potential energy (E_P) (kcal/mol); Pressure (P) (in bar), Temperature (T) (in K), and Volume (V) (in Å ^{3}) during the 100ns of molecular simulation for STOCKIN-03432 in complex with AQP-3.

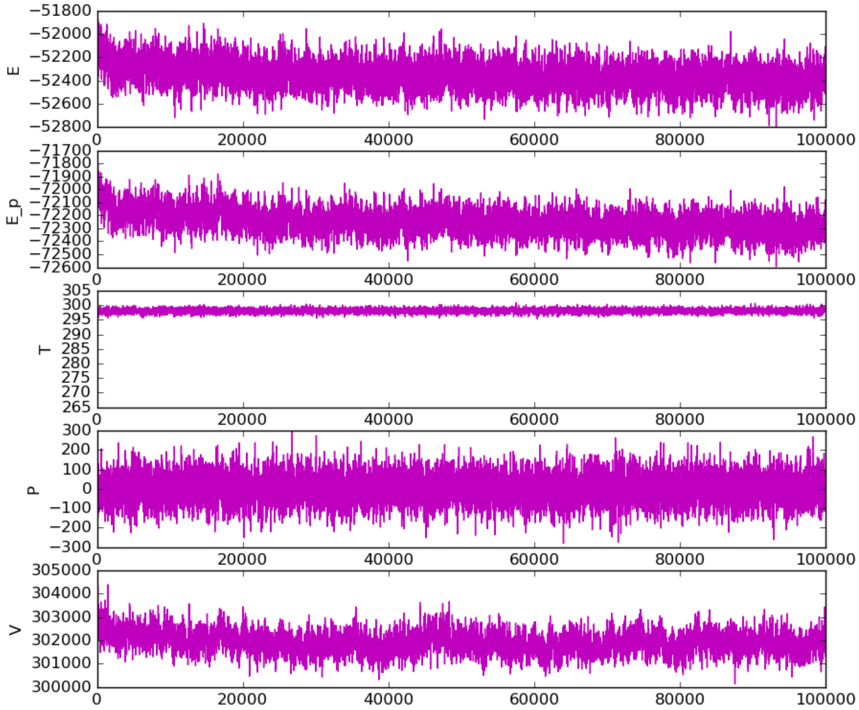

Figure S8. Total energy (E) (kcal/mol); Potential energy (E_P) (kcal/mol); Pressure (P) (in bar), Temperature (T) (in K), and Volume (V) (in Å ^{3}) during the 100ns of molecular simulation for 25284644 in complex with AQP-3.

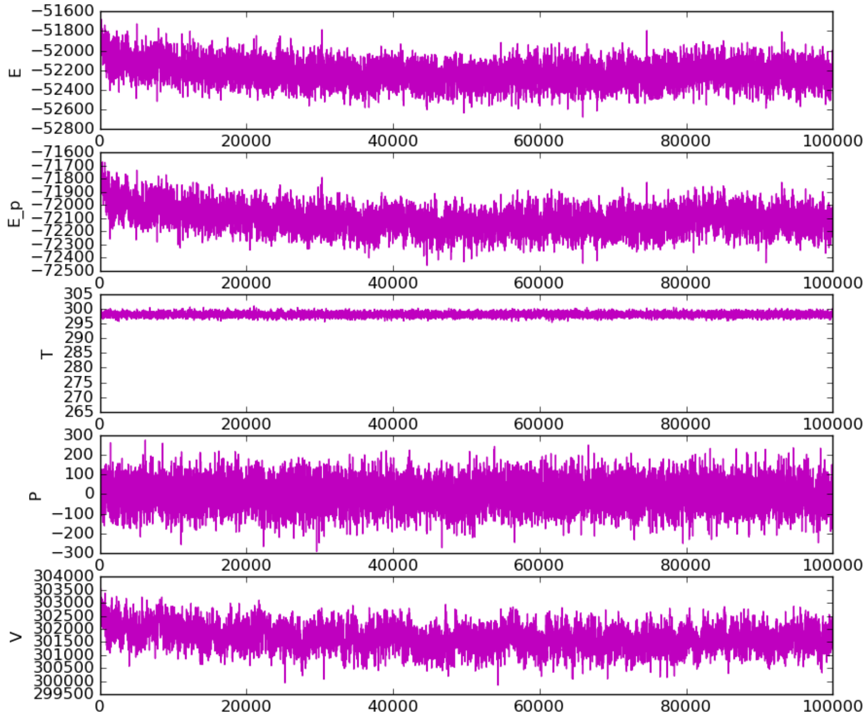

Figure S9. Total energy (E) (kcal/mol); Potential energy (E_P) (kcal/mol); Pressure (P) (in bar), Temperature (T) (in K), and Volume (V) (in Å ^{3}) during the 100ns of molecular simulation for 32927247 in complex with AQP-3.

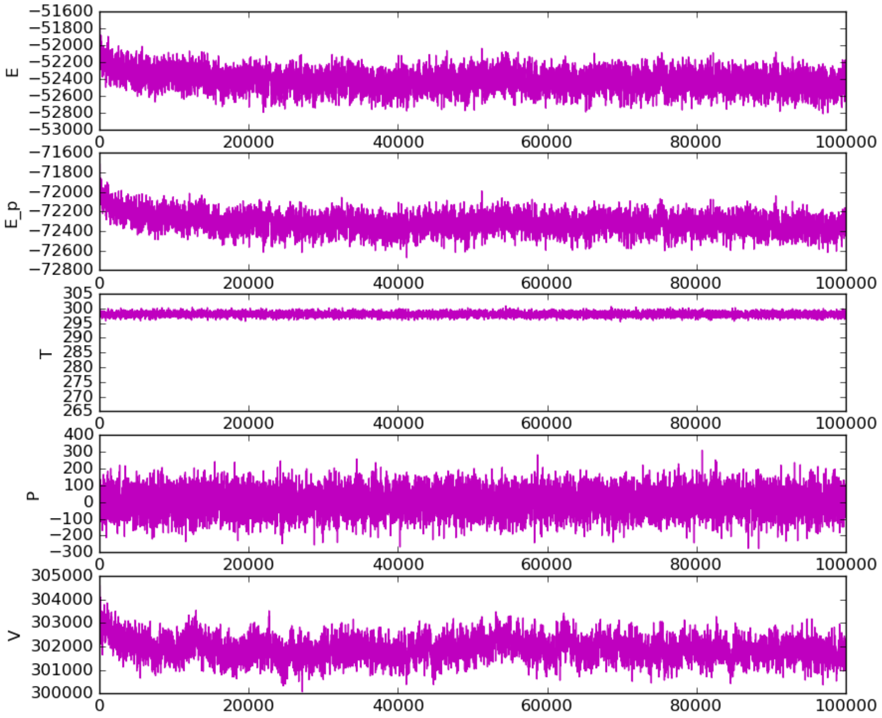

Figure S10. Total energy (E) (kcal/mol); Potential energy (E_P) (kcal/mol); Pressure (P) (in bar), Temperature (T) (in K), and Volume (V) (in Å ^{3}) during the 100ns of molecular simulation for 27371521 in complex with AQP-3.

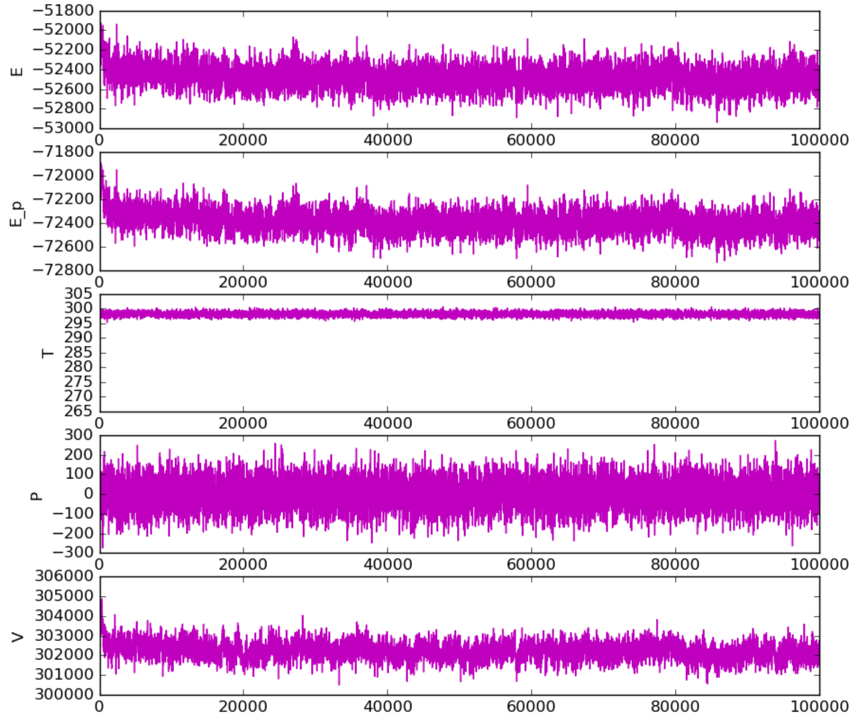

Figure S11. Total energy (E) (kcal/mol); Potential energy (E_P) (kcal/mol); Pressure (P) (in bar), Temperature (T) (in K), and Volume (V) (in Å ^{3}) during the 100ns of molecular simulation for 2801237 in complex with AQP-3.

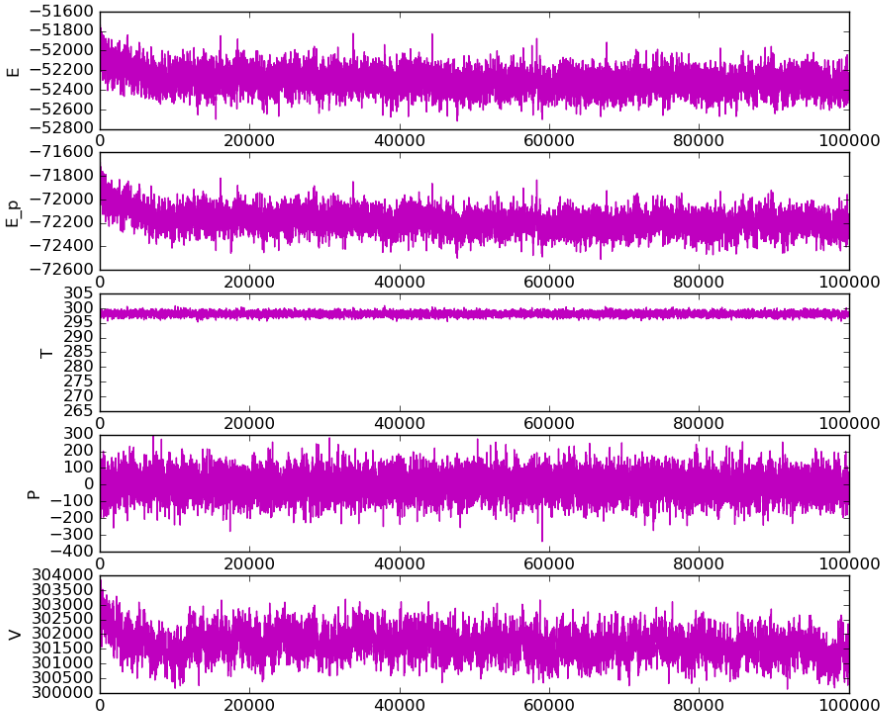

Figure S12. Total energy (E) (kcal/mol); Potential energy (E_P) (kcal/mol); Pressure (P) (in bar), Temperature (T) (in K), and Volume (V) (in Å ^{3}) during the 100ns of molecular simulation for 5633879 in complex with AQP-3.

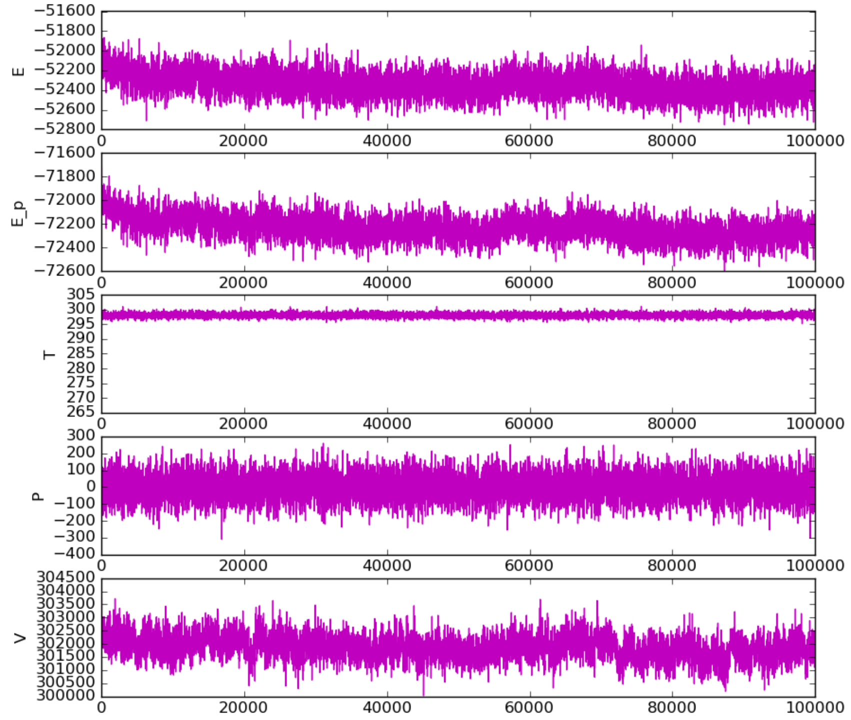

Figure S13. Total energy (E) (kcal/mol); Potential energy (E_P) (kcal/mol); Pressure (P) (in bar), Temperature (T) (in K), and Volume (V) (in Å ^{3}) during the 100ns of molecular simulation for 36994203 in complex with AQP-3.

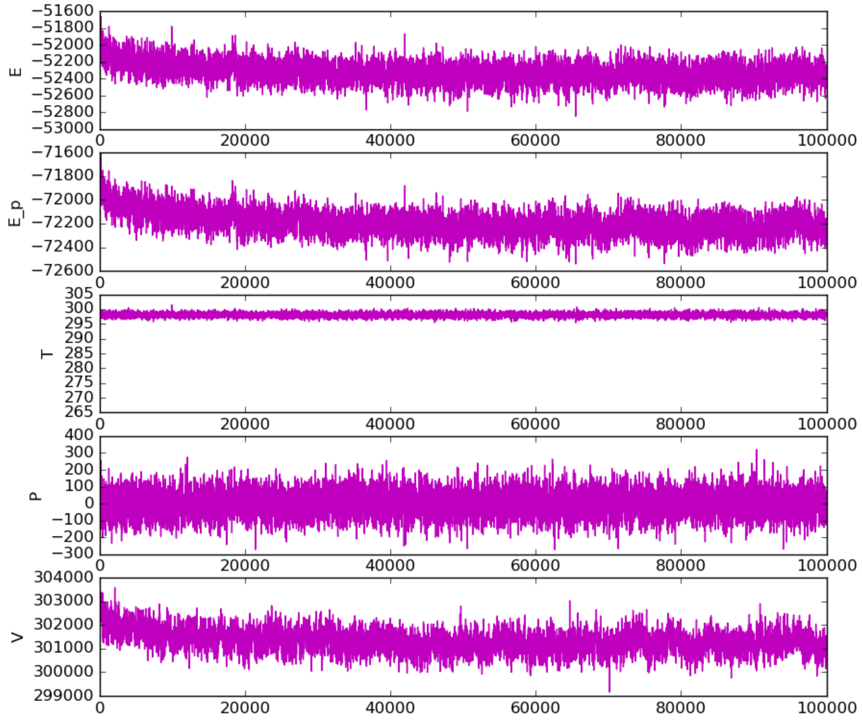

Figure S14. Total energy (E) (kcal/mol); Potential energy (E_P) (kcal/mol); Pressure (P) (in bar), Temperature (T) (in K), and Volume (V) (in Å ^{3}) during the 100ns of molecular simulation for 16694164 in complex with AQP-3.

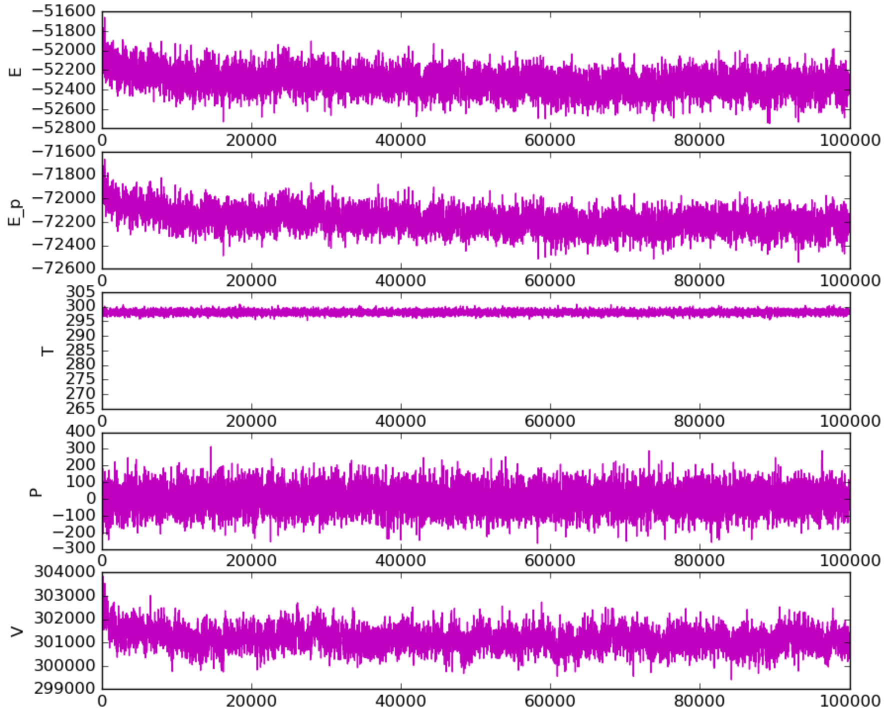

Figure S15. Total energy (E) (kcal/mol); Potential energy (E_P) (kcal/mol); Pressure (P) (in bar), Temperature (T) (in K), and Volume (V) (in Å ^{3}) during the 100ns of molecular simulation for 13477729 in complex with AQP-3.

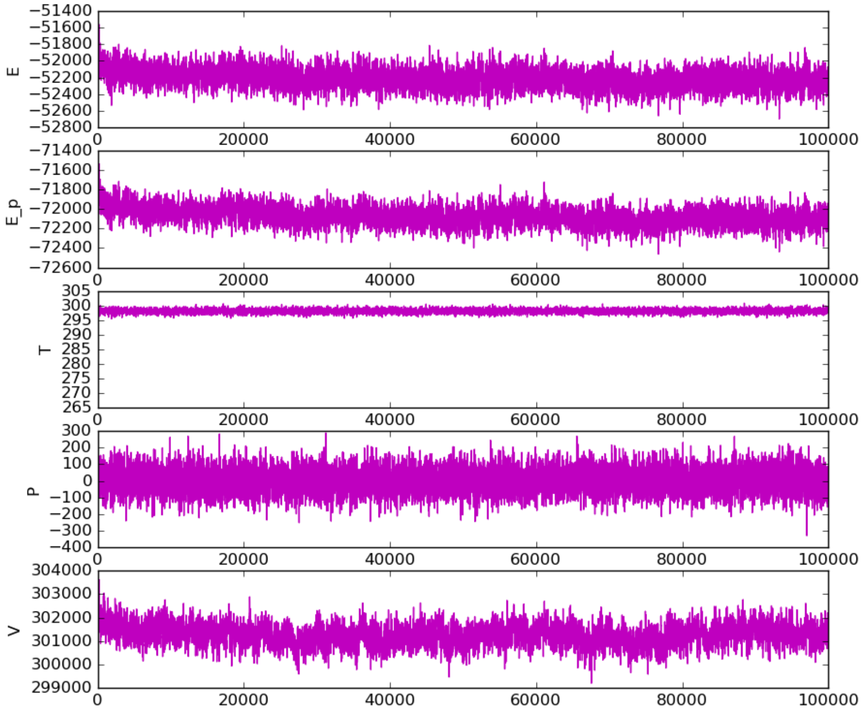

Figure S16. Total energy (E) (kcal/mol); Potential energy (E_P) (kcal/mol); Pressure (P) (in bar), Temperature (T) (in K), and Volume (V) (in Å ^{3}) during the 100ns of molecular simulation for 36657947 in complex with AQP-3.

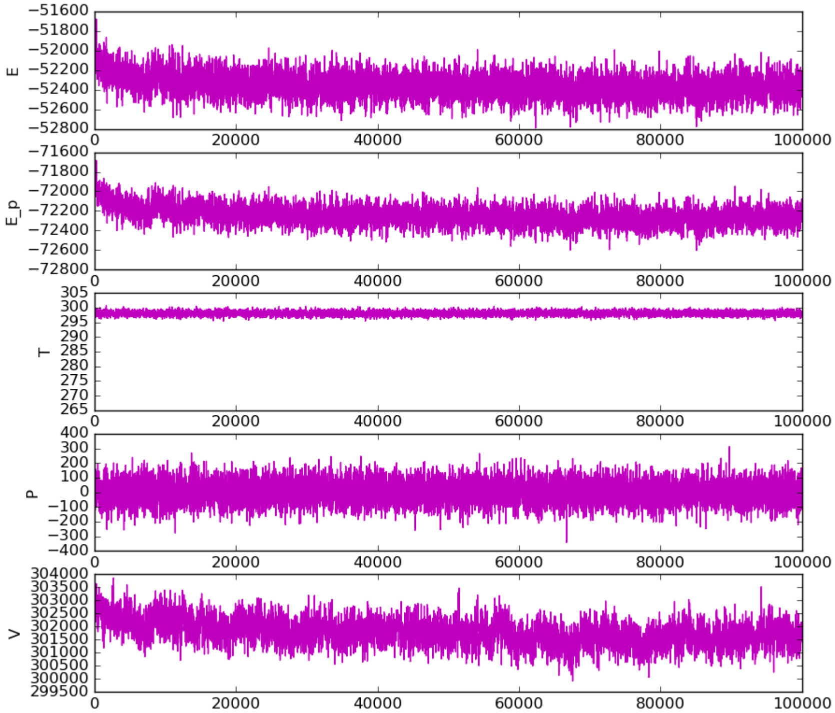

Figure S17. Total energy (E) (kcal/mol); Potential energy (E_P) (kcal/mol); Pressure (P) (in bar), Temperature (T) (in K), and Volume (V) (in Å ^{3}) during the 100ns of molecular simulation for 36716128 in complex with AQP-3.

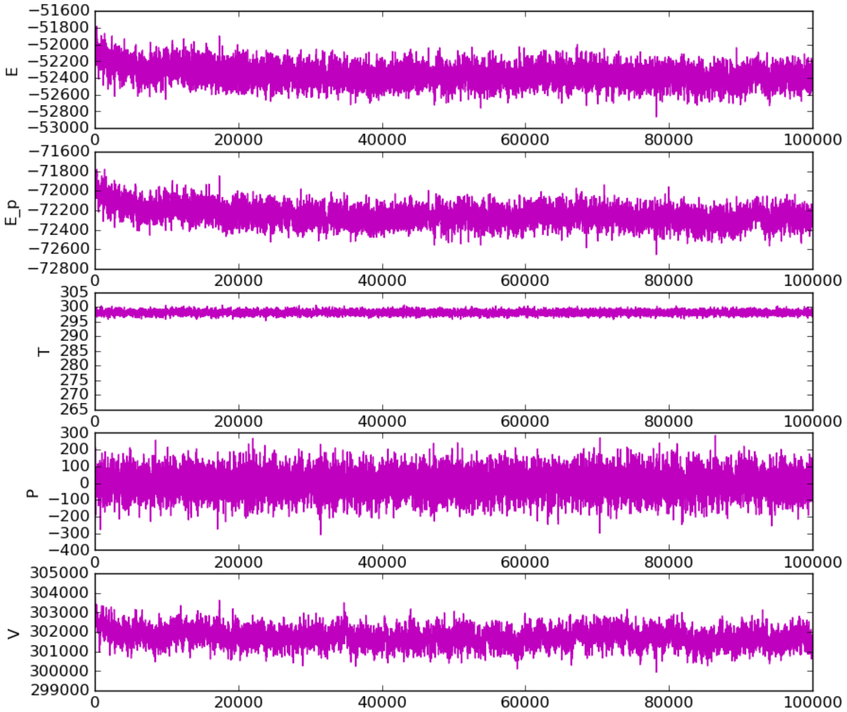

Figure S18. Total energy (E) (kcal/mol); Potential energy (E_P) (kcal/mol); Pressure (P) (in bar), Temperature (T) (in K), and Volume (V) (in Å ^{3}) during the 100ns of molecular simulation for 3325122 in complex with AQP-3.

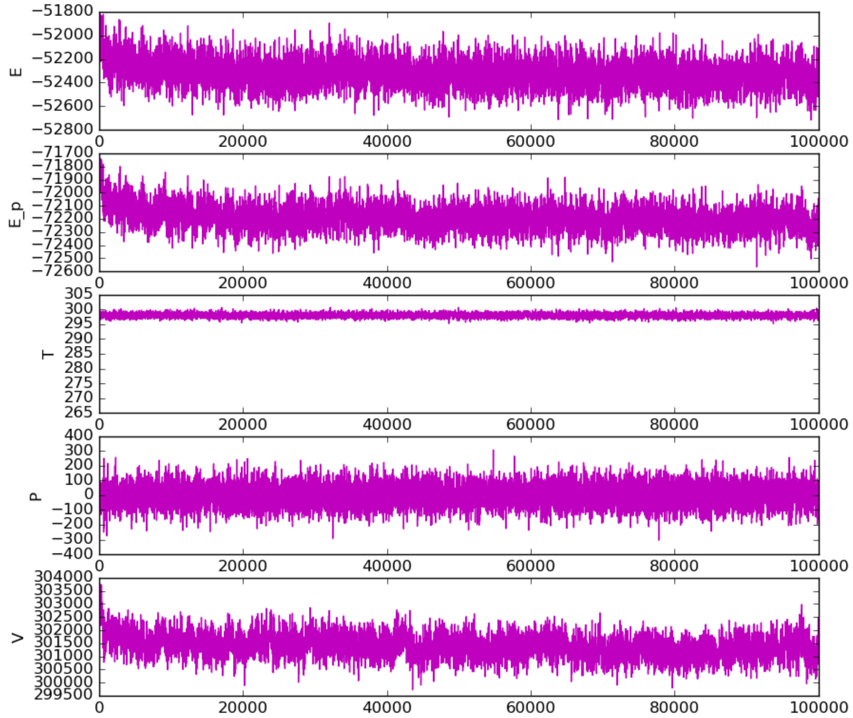

Figure S19. Total energy (E) (kcal/mol); Potential energy (E_P) (kcal/mol); Pressure (P) (in bar), Temperature (T) (in K), and Volume (V) (in Å ^{3}) during the 100ns of molecular simulation for 42888719 in complex with AQP-3.

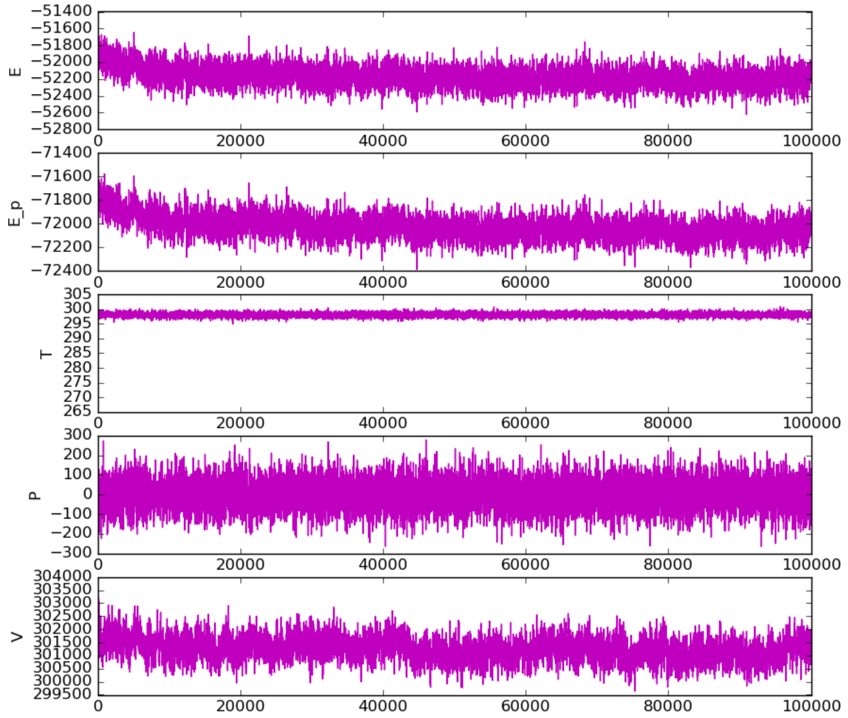

Figure S20. Total energy (E) (kcal/mol); Potential energy (E_P) (kcal/mol); Pressure (P) (in bar), Temperature (T) (in K), and Volume (V) (in Å ^{3}) during the 100ns of molecular simulation for IP6 in complex with AQP-3.

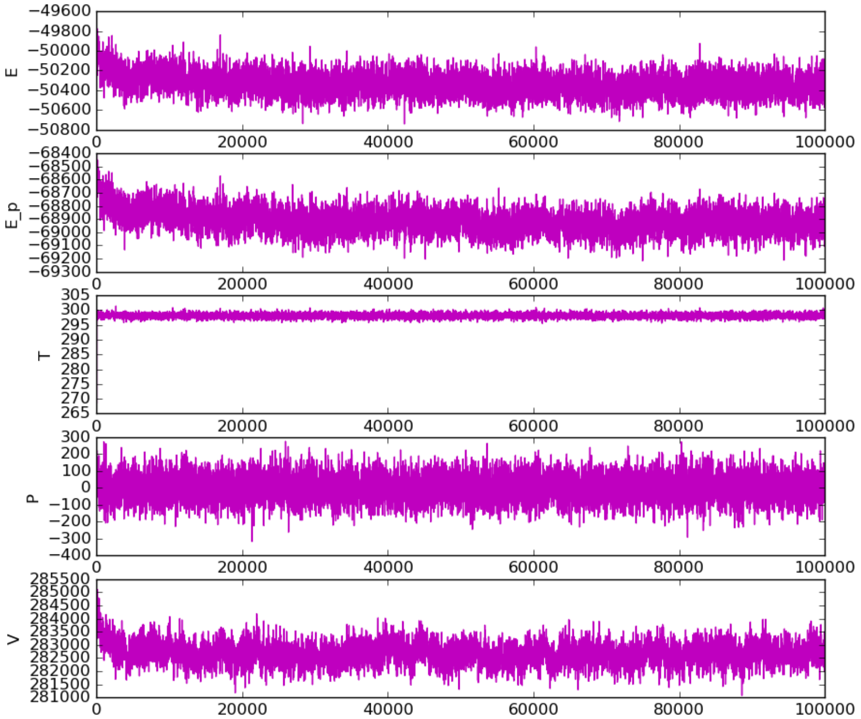

Figure S21. Total energy (E) (kcal/mol); Potential energy (E_P) (kcal/mol); Pressure (P) (in bar), Temperature (T) (in K), and Volume (V) (in Å ^{3}) during the 100ns of molecular simulation for 31879059 in complex with AQP-3.

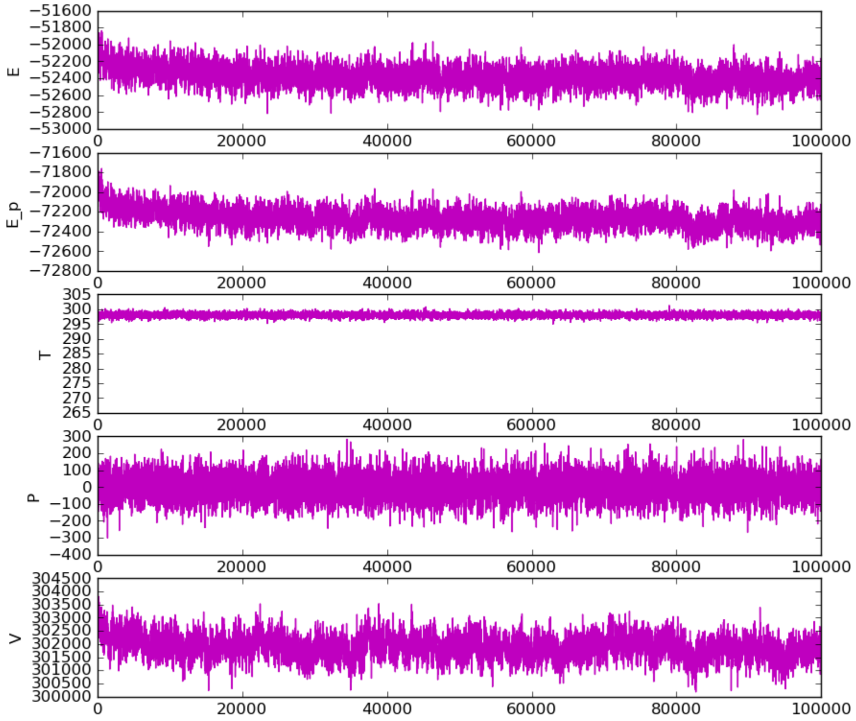

Figure S22. Total energy (E) (kcal/mol); Potential energy (E_P) (kcal/mol); Pressure (P) (in bar), Temperature (T) (in K), and Volume (V) (in Å ^{3}) during the 100ns of molecular simulation for 31966421 in complex with AQP-3.

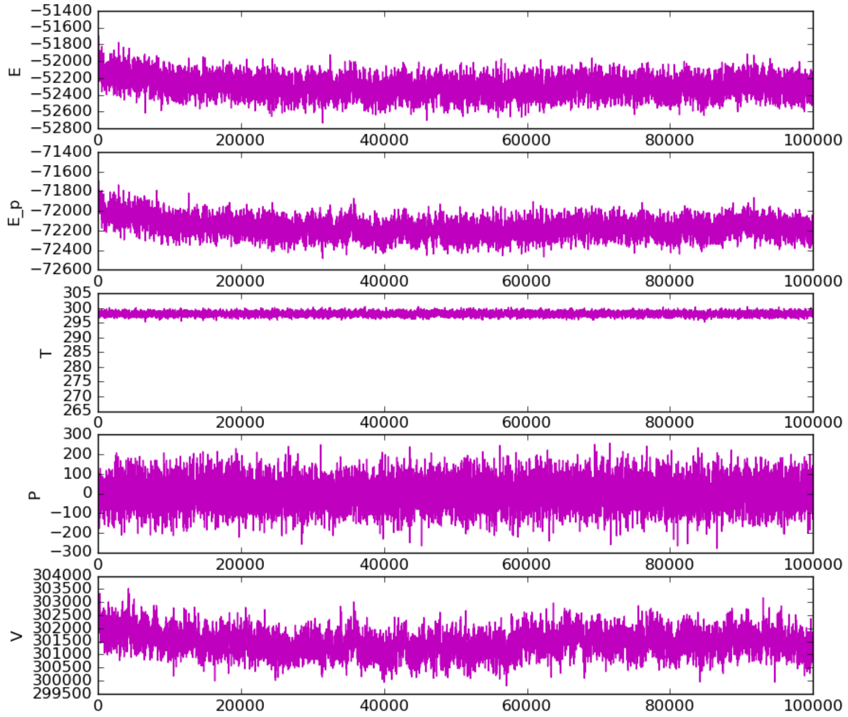

Figure S23. Total energy (E) (kcal/mol); Potential energy (E_P) (kcal/mol); Pressure (P) (in bar), Temperature (T) (in K), and Volume (V) (in Å ^{3}) during the 100ns of molecular simulation for 7658775 in complex with AQP-3.

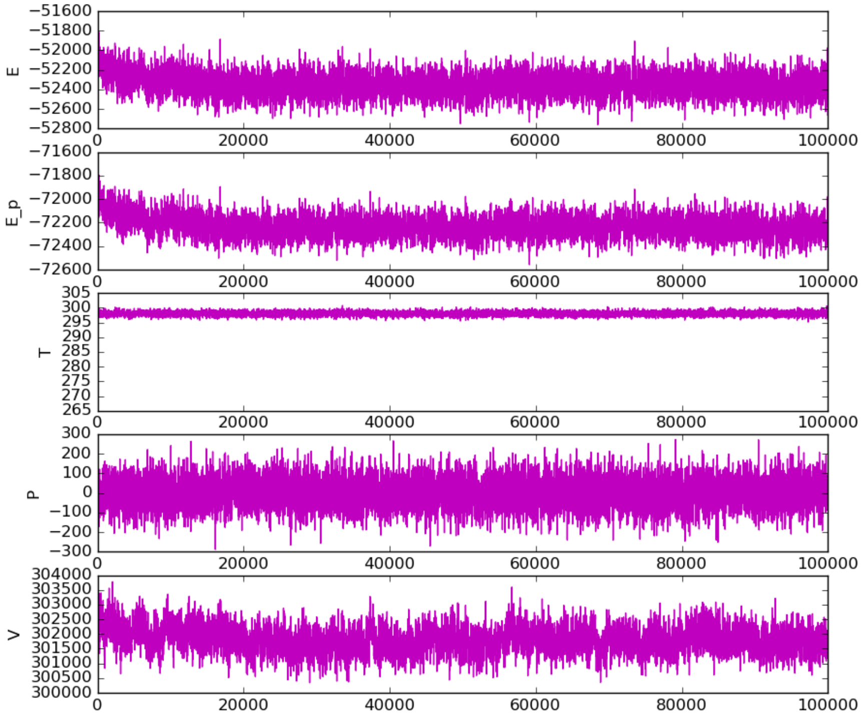

Figure S24. Total energy (E) (kcal/mol); Potential energy (E_P) (kcal/mol); Pressure (P) (in bar), Temperature (T) (in K), and Volume (V) (in Å ^{3}) during the 100ns of molecular simulation for 25665268 in complex with AQP-3.

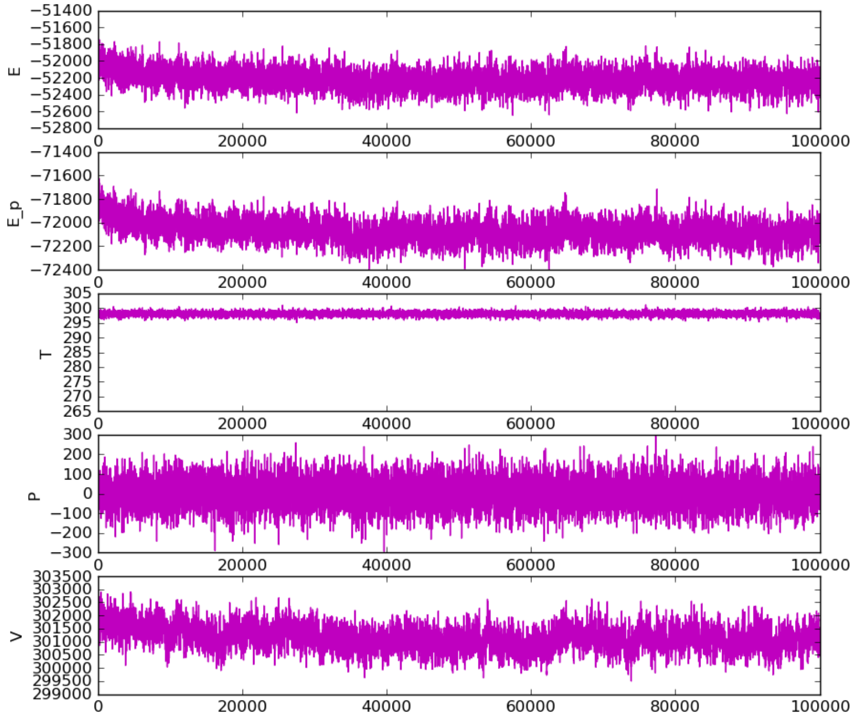

Figure S25. Total energy (E) (kcal/mol); Potential energy (E_P) (kcal/mol); Pressure (P) (in bar), Temperature (T) (in K), and Volume (V) (in Å ^{3}) during the 100ns of molecular simulation for 37101119 in complex with AQP-3.

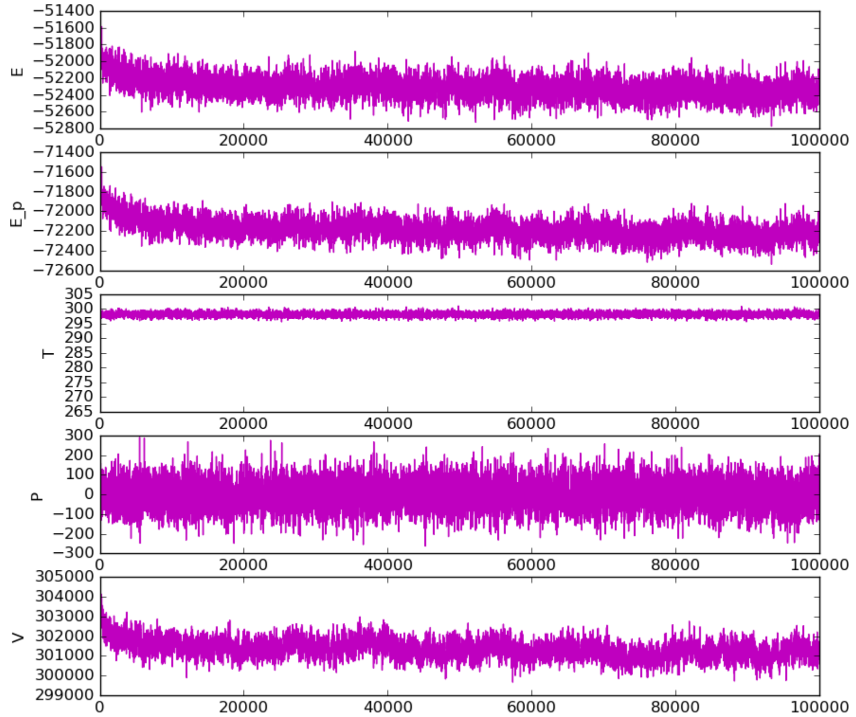

Figure S26. Time dependence of root mean squared deviation (RMSD) of Cα-atoms of AQP-3 in complex with CMPD01, CMPD02, CMP03, CMPD04, CMPD05.

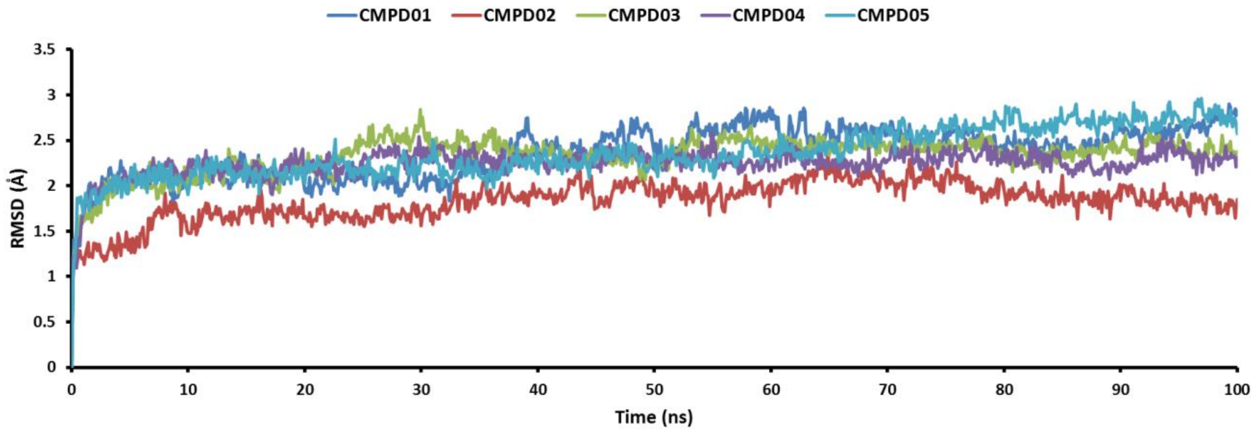

Figure S27. Time dependence of root mean squared deviation (RMSD) of Cα-atoms of AQP-3 in complex with CMPD06, CMPD07, CMP08, CMPD09, CMPD10.

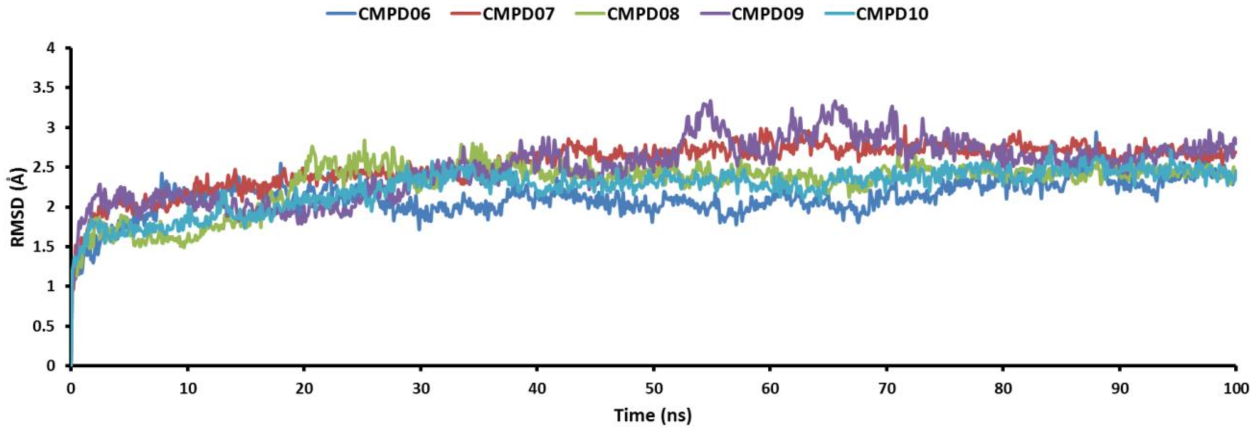

Figure S28. Time dependence of root mean squared deviation (RMSD) of Cα-atoms of AQP-3 in complex with CMPD11, CMPD12, CMP13, CMPD14, CMPD15.

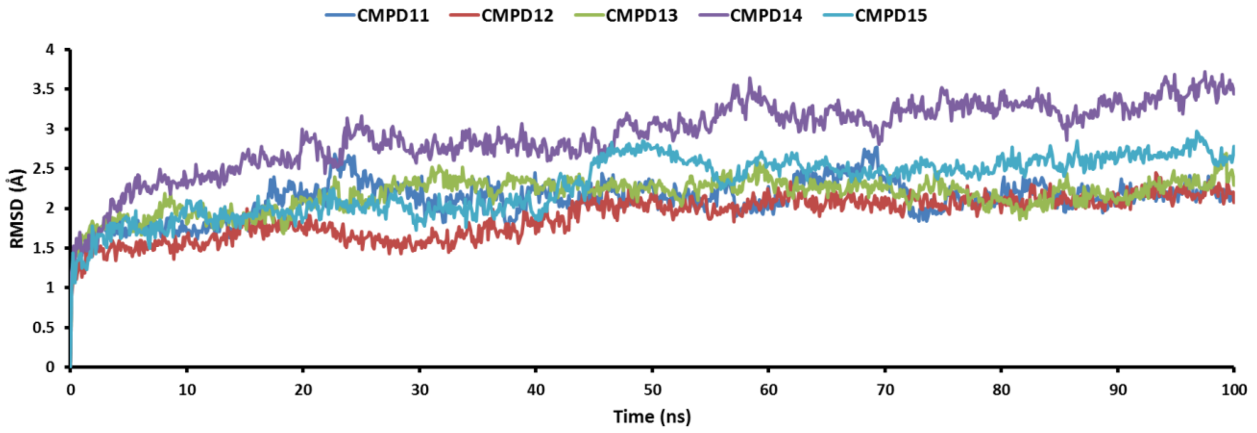

Figure S29. Time dependence of root mean squared deviation (RMSD) of Cα-atoms of AQP-3 in complex with CMPD16, CMPD17, CMP18, CMPD19, CMPD20.

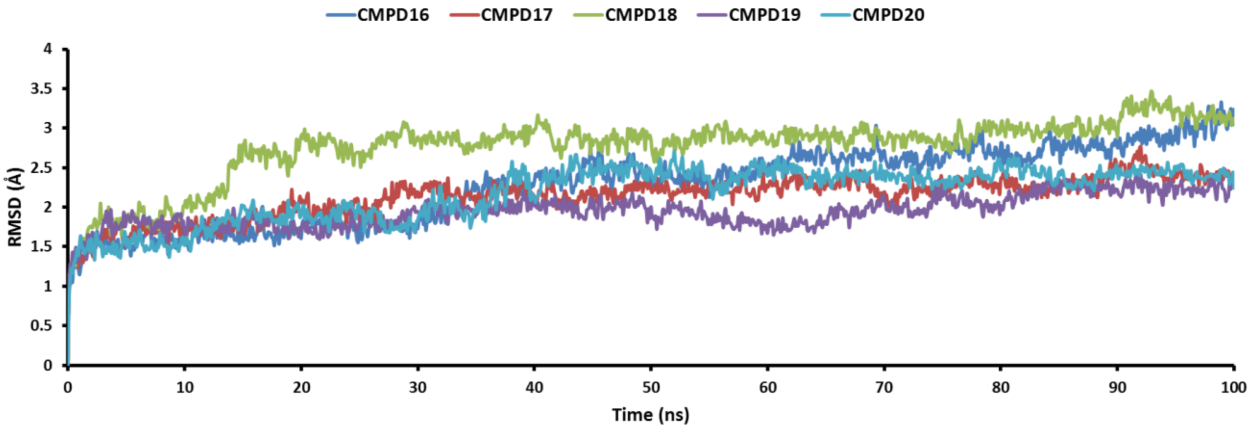

Abbreviations:
CMPD01: C8C
CMPD02: STOCKIN-03432
CMPD03: 25284644
CMPD04: 32927247
CMPD05: 27371521
CMPD06: 2801237
CMPD07: 5633879
CMPD08: 36994203
CMPD09: 16694164
CMPD10: 13477729
CMPD11: 36657947
CMPD12: 36716128
CMPD13: 3325122
CMPD14: 42888719
CMPD15: IP6
CMPD16: 31879059
CMPD17: 31966421
CMPD18: 7658775
CMPD19: 25665268
CMPD20: 37101119

Figure S30. (a) Root mean squared fluctuations (RMSFs) of Cα-atoms along with contacts (green-colored vertical bars) of C8C in complex with AQP-3; (b) Protein ligand contacts histogram throughout the simulation for C8C in complex with AQP-3; (c) Protein-ligand interaction profile for C8C in complex with AQP-3; (d) Hydrogen bond interaction observed between protein and C8C in different frames throughout the simulation; (e) the secondary structure elements (SSE) for protein conformation during the simulation (% of Total SSE calculated were 50.63)

(a)

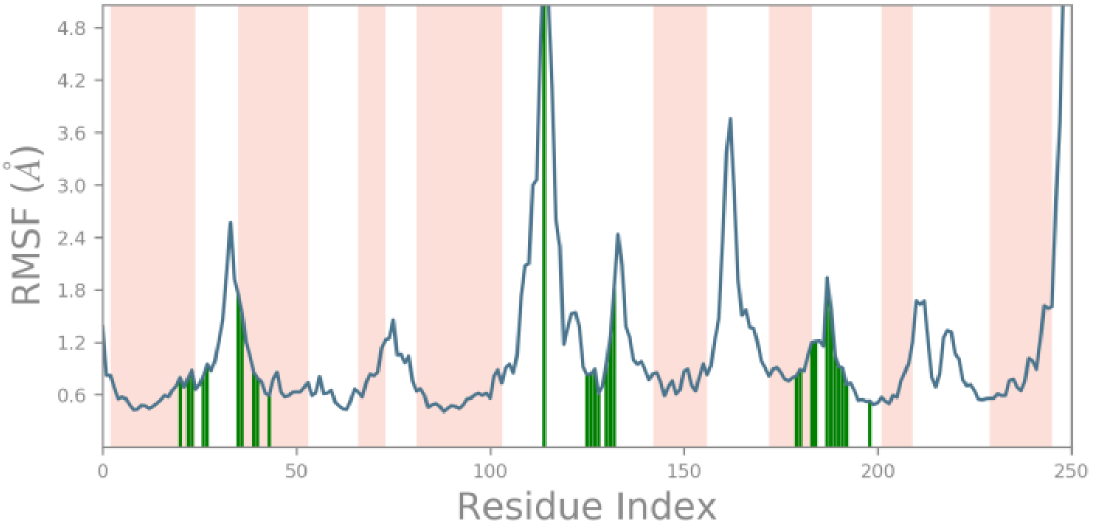

(b)

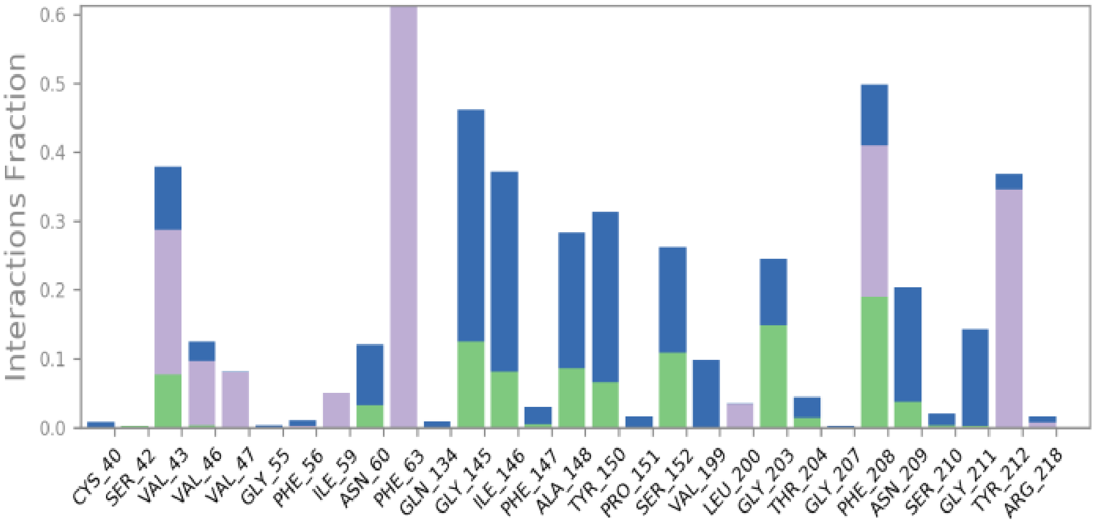

(c)

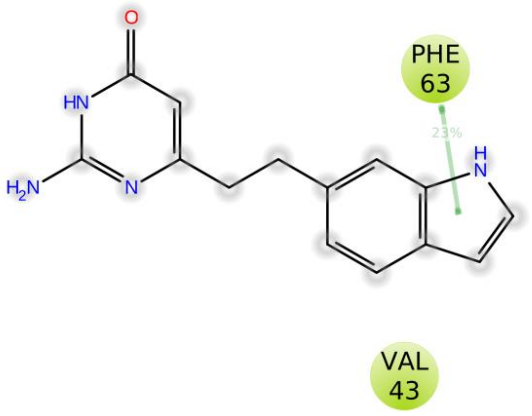
O
HN
H_2N
N
H
N
PHE
63
23%
VAL
43

(d)

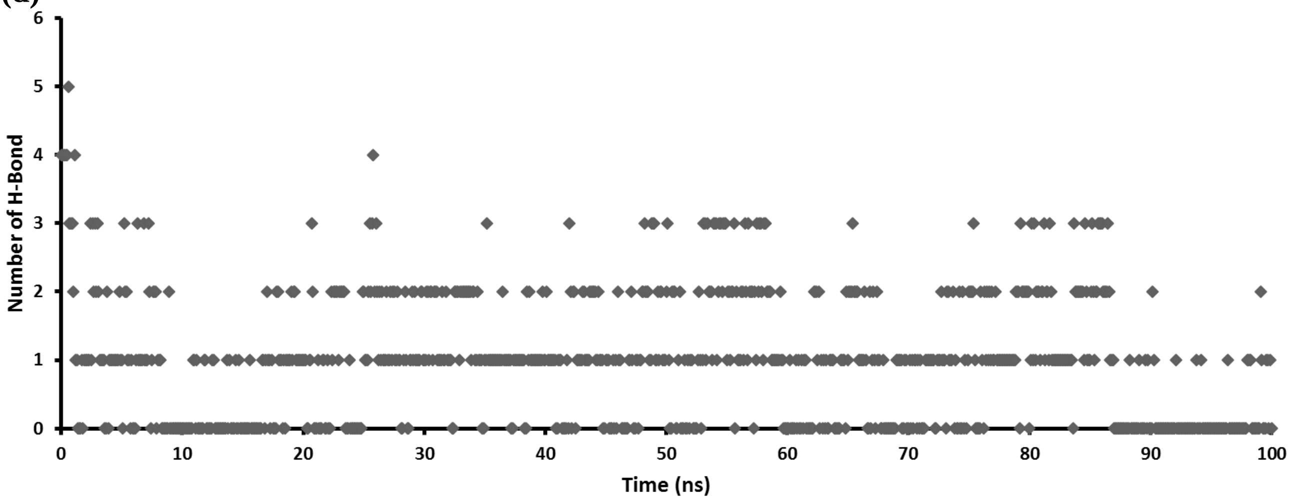
Number of H-Bond
Time (ns)
0
1
2
3
4
5
6
0
10
20
30
40
50
60
70
80
90
100

(e)

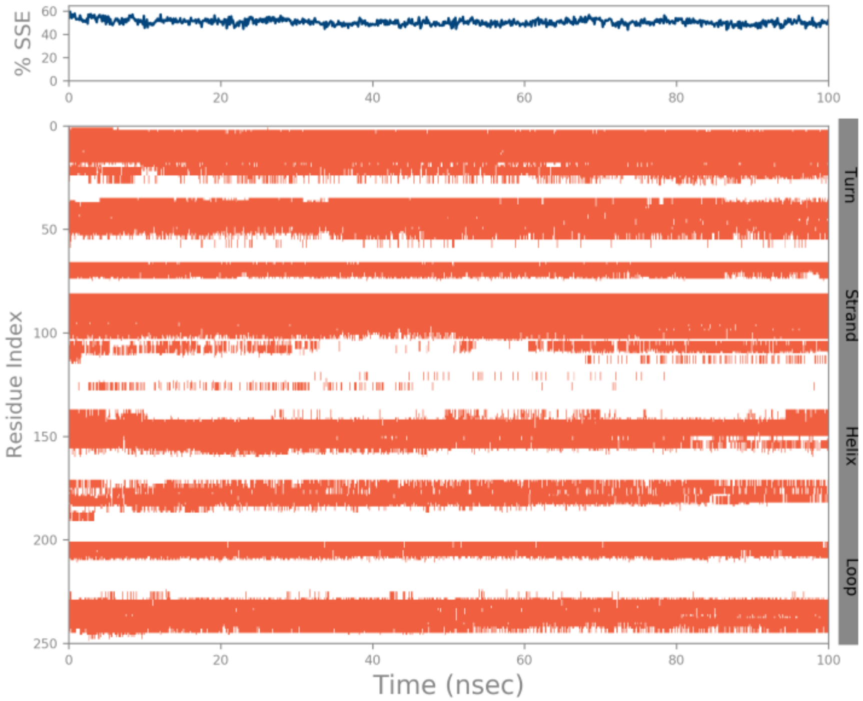
% SSE
Residue Index
Time (nsec)
Turn
Strand
Helix
Loop

Figure S31. (a) Root mean squared fluctuations (RMSFs) of Cα-atoms along with contacts (green-colored vertical bars) of STOCKIN-03432 in complex with AQP-3; (b) Protein ligand contacts histogram throughout the simulation for STOCKIN-03432 in complex with AQP-3; (c) Protein-ligand interaction profile for STOCKIN-03432 in complex with AQP-3; (d) Hydrogen bond interaction observed between protein and STOCKIN-03432 in different frames throughout the simulation; (e) the secondary structure elements (SSE) for protein conformation during the simulation (% of Total SSE calculated were 57.14).

(a)

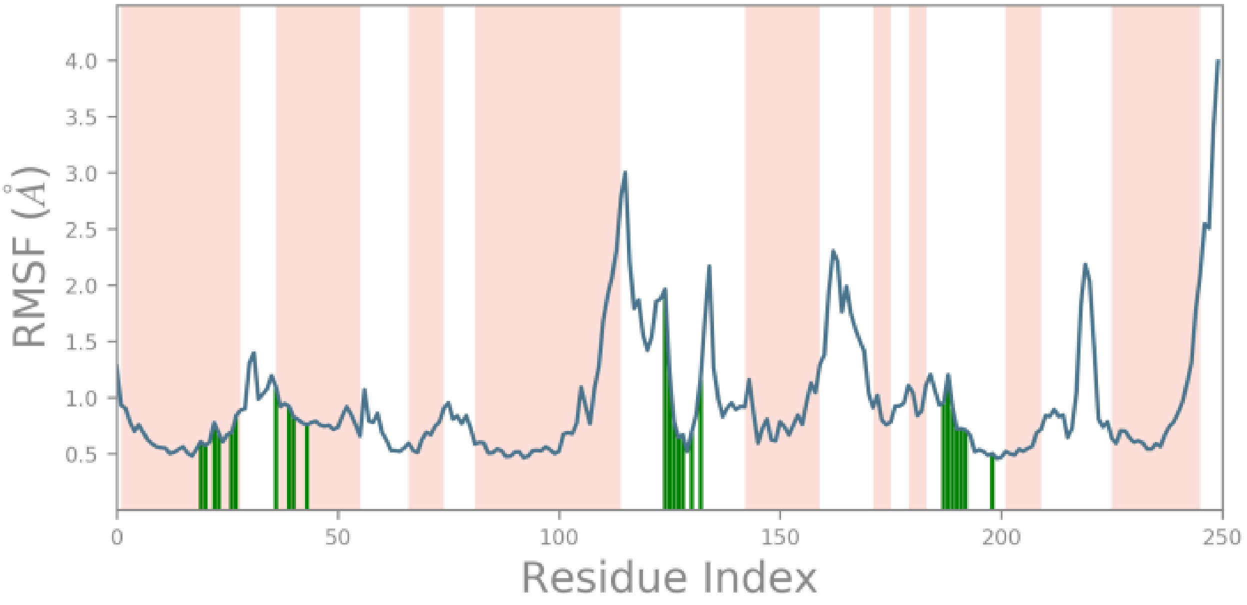

(b)

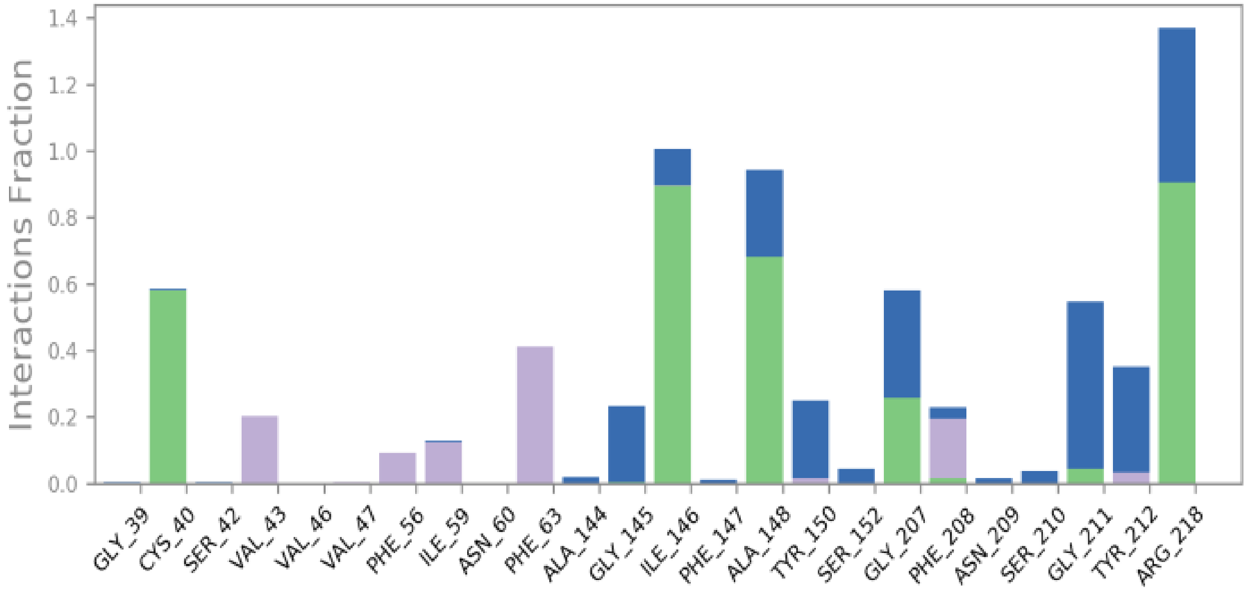

(c)

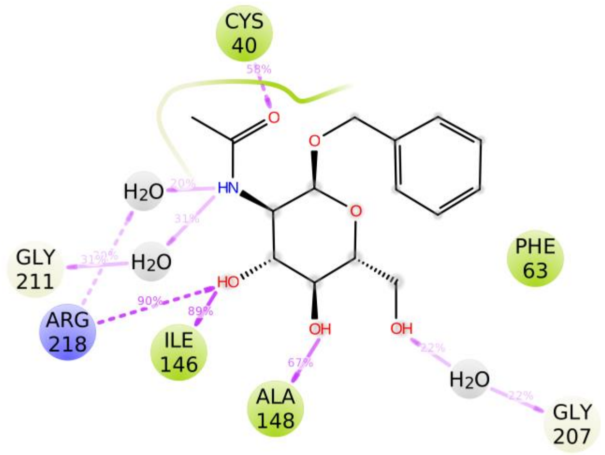
CYS 40
58%
H2O
20%
HN
31%
GLY 211
31%
30%
H2O
HO
90%
89%
ARG 218
ILE 146
OH
67%
ALA 148
OH
22%
H2O
22%
GLY 207
PHE 63

(d)

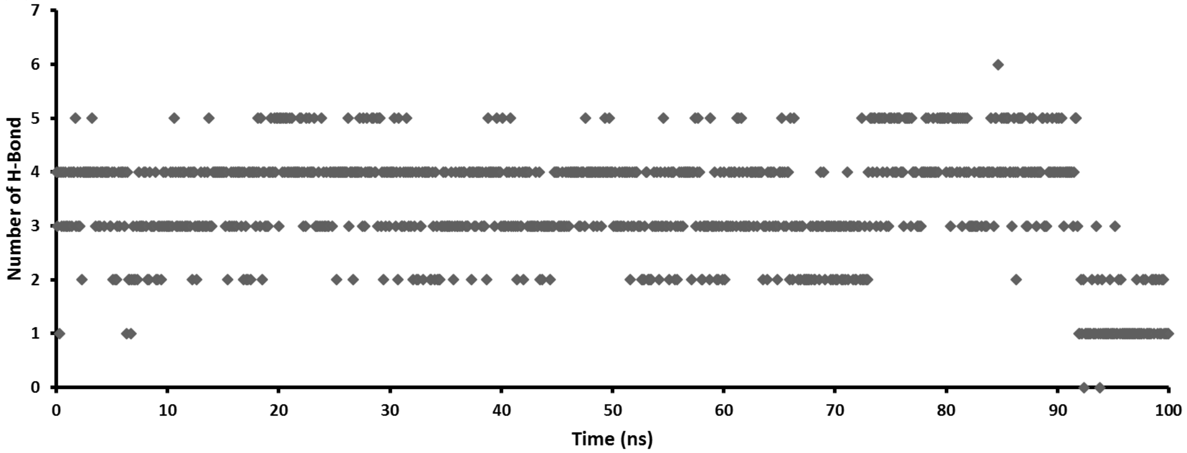
Number of H-Bond
Time (ns)
0
1
2
3
4
5
6
7
0
10
20
30
40
50
60
70
80
90
100

(e)

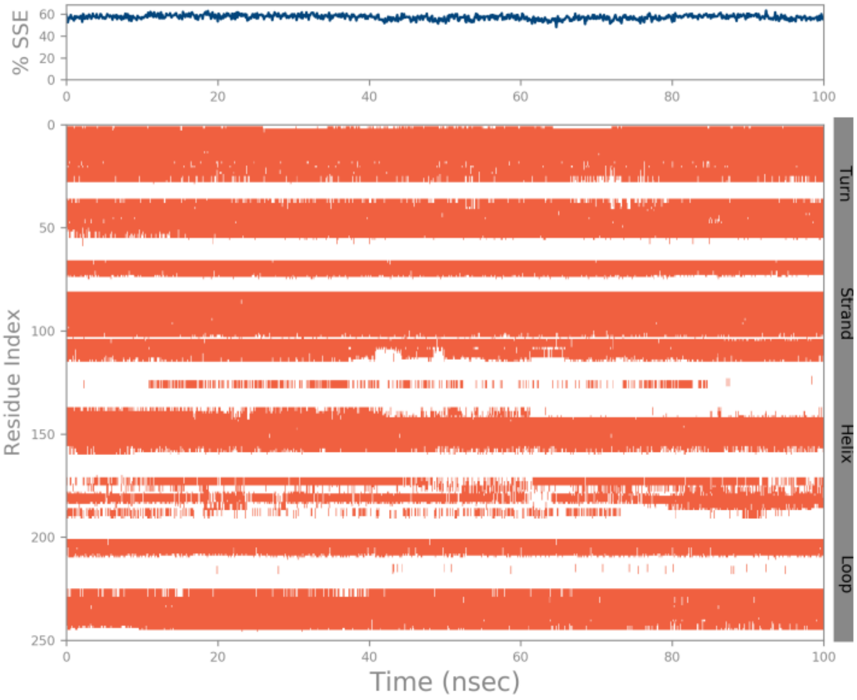
% SSE
Residue Index
Time (nsec)
Turn
Strand
Helix
Loop

Figure S32. (a) Root mean squared fluctuations (RMSFs) of Cα-atoms along with contacts (green-colored vertical bars) of 25284644 in complex with AQP-3; (b) Protein ligand contacts histogram throughout the simulation for 25284644 in complex with AQP-3; (c) Protein-ligand interaction profile for 25284644 in complex with AQP-3; (d) Hydrogen bond interaction observed between protein and 25284644 in different frames throughout the simulation; (e) the secondary structure elements (SSE) for protein conformation during the simulation (% of Total SSE calculated were 52.26).

(a)

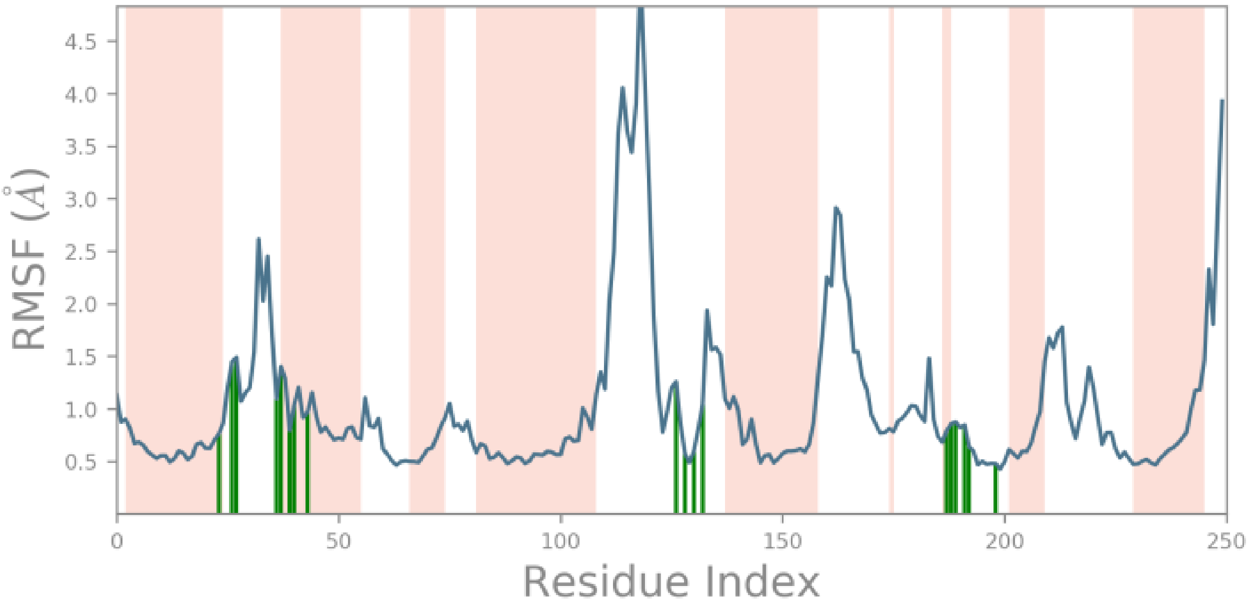

(b)

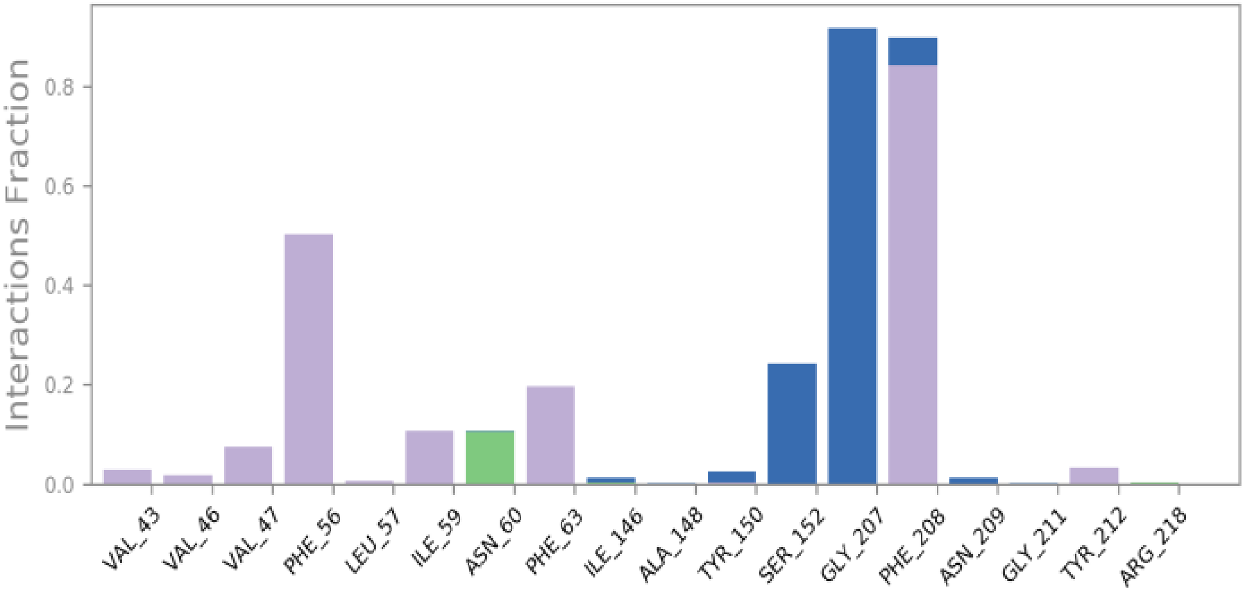

(c)

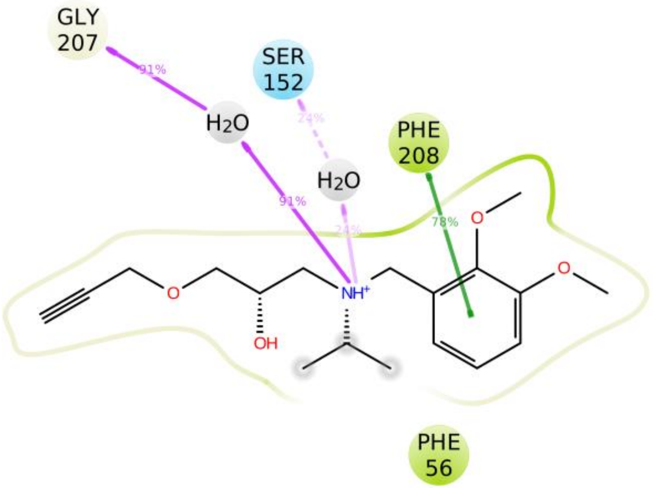

GLY 207
SER 152
H2O
H2O
PHE 208
91%
91%
24%
24%
78%
O
O
O
OH
NH+
PHE 56

(d)

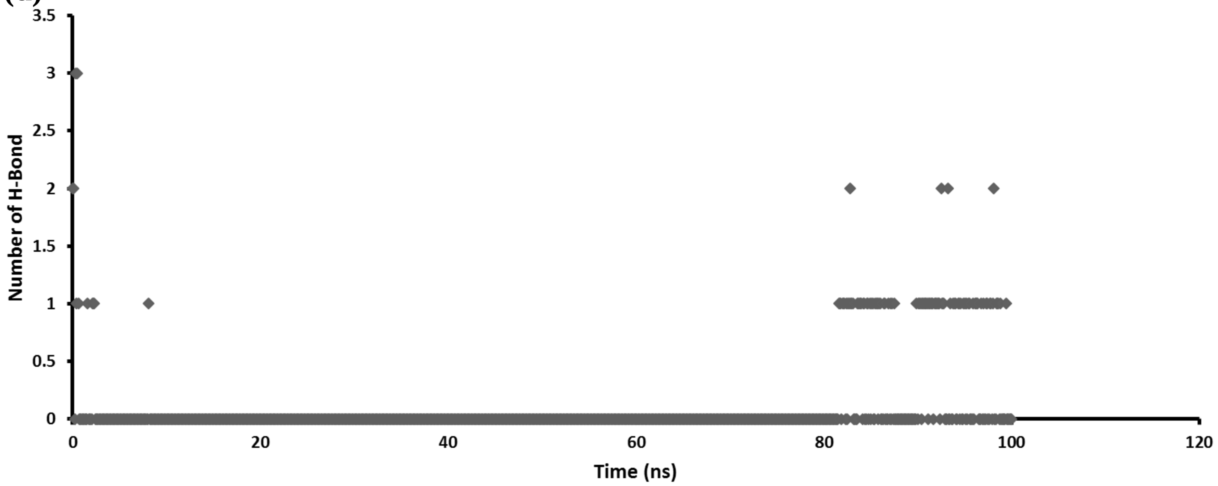

Number of H-Bond
3.5
3
2.5
2
1.5
1
0.5
0
0
20
40
60
80
100
120
Time (ns)

(e)

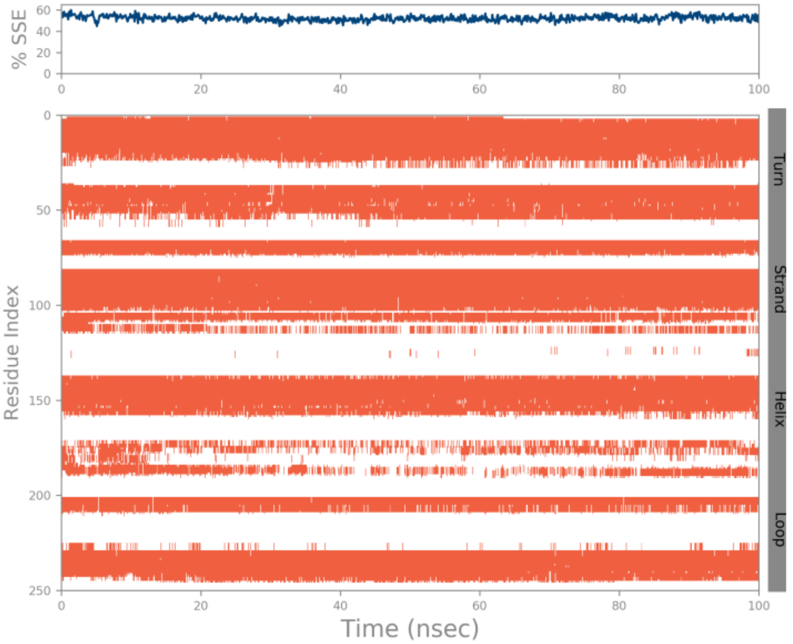

% SSE
60
40
20
0
Residue Index
0
50
100
150
200
250
Turn
Strand
Helix
Loop
Time (nsec)

Figure S33. (a) Root mean squared fluctuations (RMSFs) of Cα-atoms along with contacts (green-colored vertical bars) of 32927247 in complex with AQP-3; (b) Protein ligand contacts histogram throughout the simulation for 32927247 in complex with AQP-3; (c) Protein-ligand interaction profile for 32927247 in complex with AQP-3; (d) Hydrogen bond interaction observed between protein and 32927247 in different frames throughout the simulation; (e) the secondary structure elements (SSE) for protein conformation during the simulation (% of Total SSE calculated were 53.89).

(a)

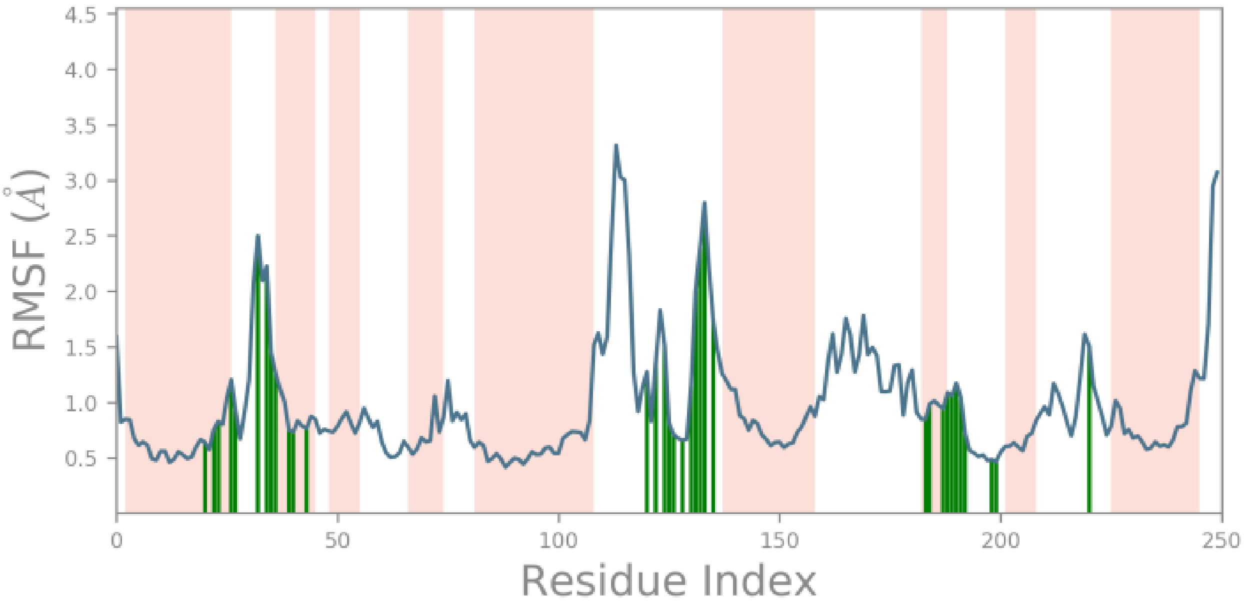

(b)

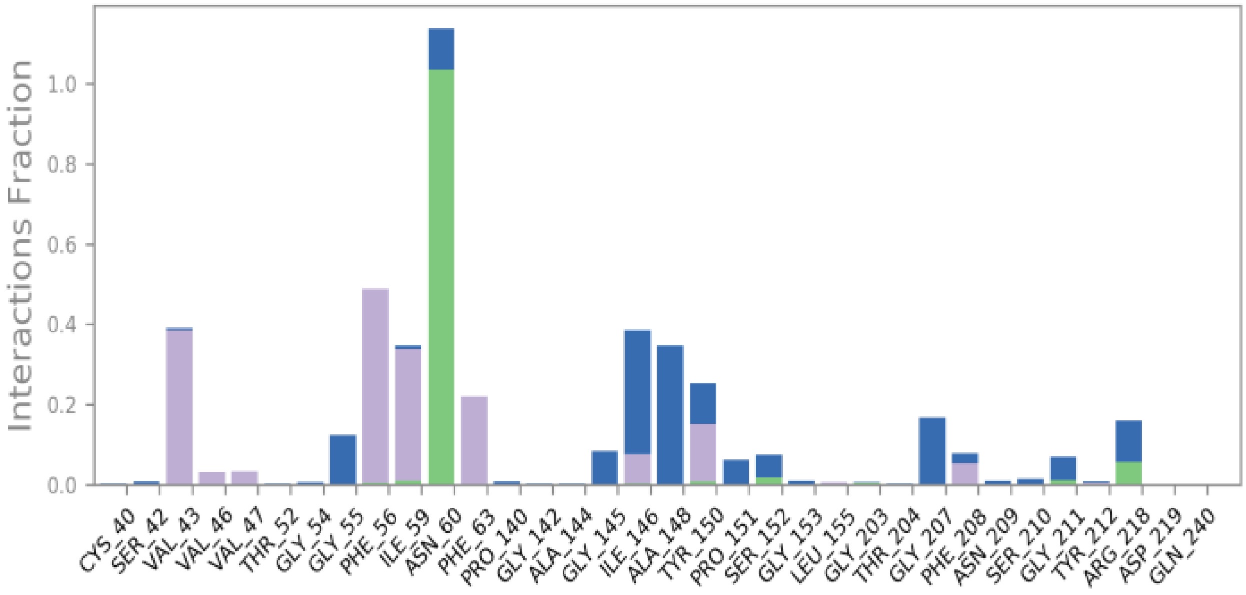

(c)

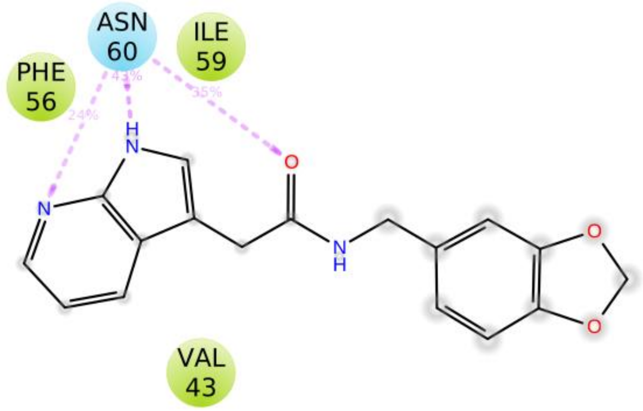
ASN 60
ILE 59
PHE 56
VAL 43
43%
35%
24%

(d)

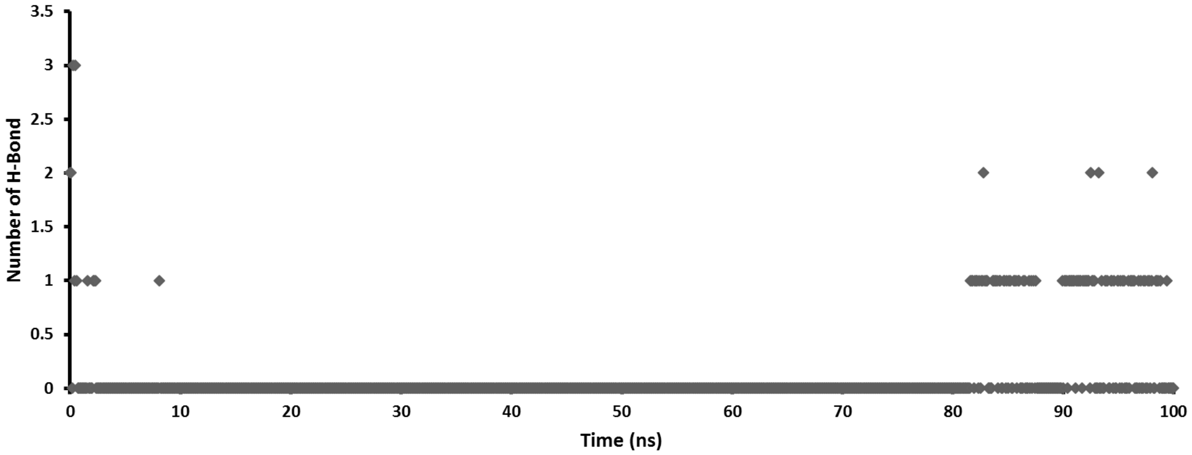
Number of H-Bond
Time (ns)

(e)

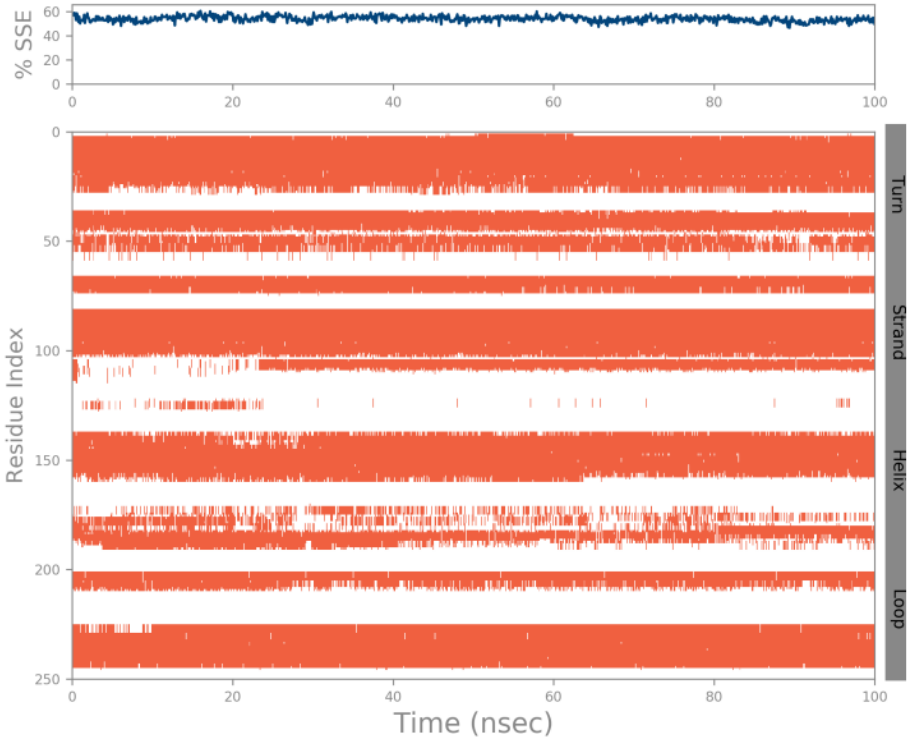
% SSE
Residue Index
Time (nsec)
Turn
Strand
Helix
Loop

Figure S34. (a) Root mean squared fluctuations (RMSFs) of Cα-atoms along with contacts (green-colored vertical bars) of 27371521 in complex with AQP-3; (b) Protein ligand contacts histogram throughout the simulation for 27371521 in complex with AQP-3; (c) Protein-ligand interaction profile for 27371521 in complex with AQP-3; (d) Hydrogen bond interaction observed between protein and 27371521 in different frames throughout the simulation; (e) the secondary structure elements (SSE) for protein conformation during the simulation (% of Total SSE calculated were 51.67).

(a)

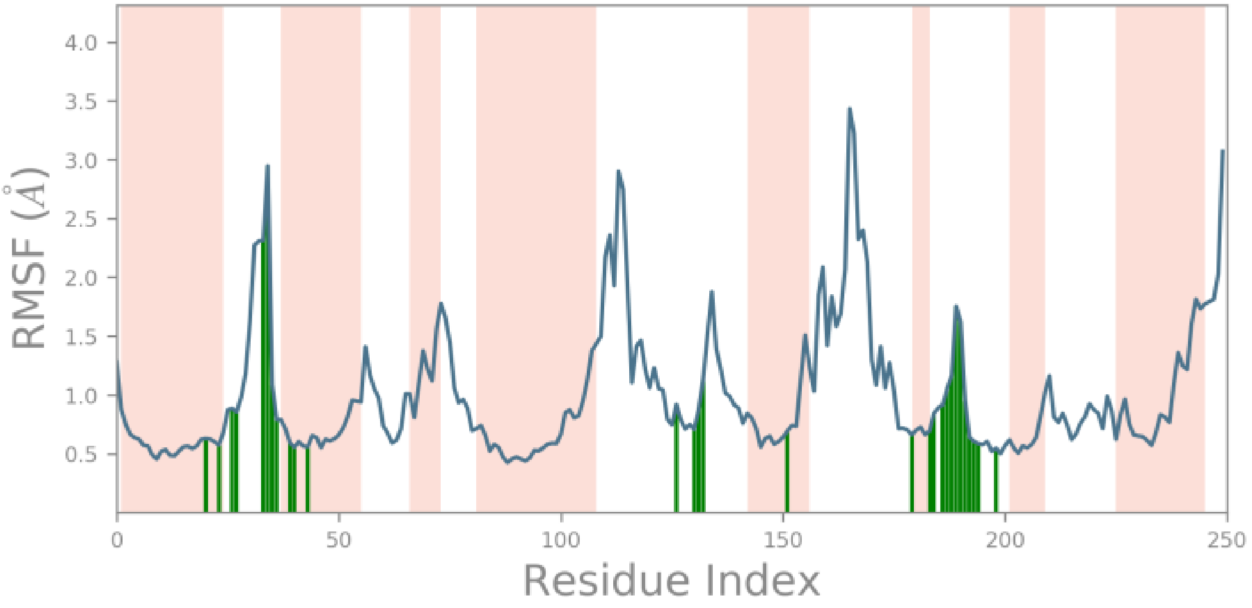

(b)

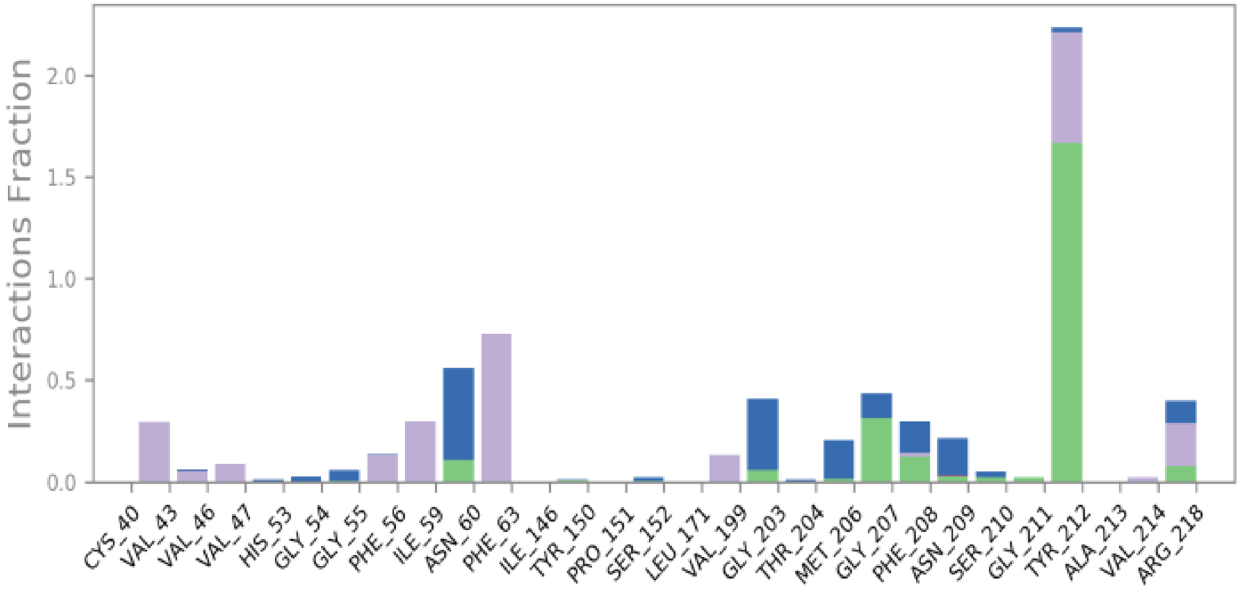

(c)

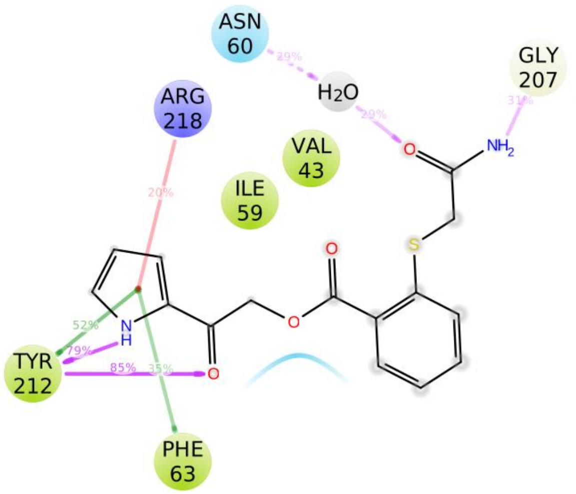

ASN 60
GLY 207
ARG 218
VAL 43
ILE 59
TYR 212
PHE 63

(d)

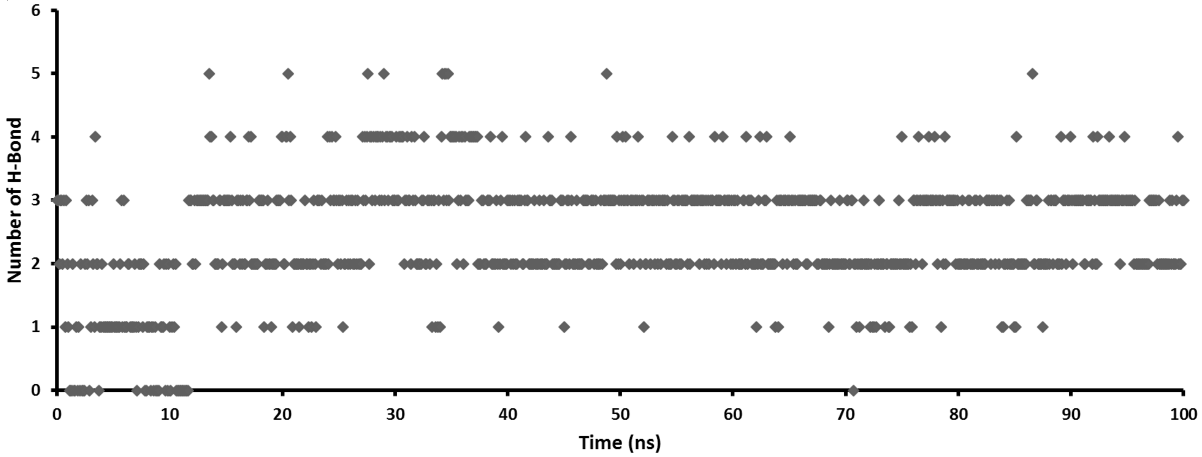

Number of H-Bond
Time (ns)

(e)

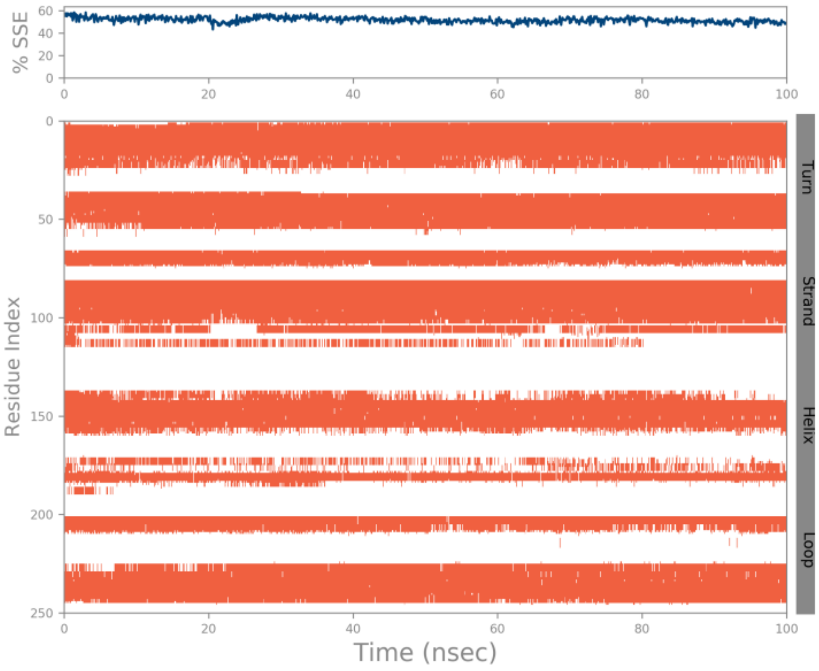

% SSE
Residue Index
Time (nsec)
Turn
Strand
Helix
Loop

Figure S35. (a) Root mean squared fluctuations (RMSFs) of Cα-atoms along with contacts (green-colored vertical bars) of 2801237 in complex with AQP-3; (b) Protein ligand contacts histogram throughout the simulation for 2801237 in complex with AQP-3; (c) Protein-ligand interaction profile for 2801237 in complex with AQP-3; (d) Hydrogen bond interaction observed between protein and 2801237 in different frames throughout the simulation; (e) the secondary structure elements (SSE) for protein conformation during the simulation (% of Total SSE calculated were 52.77).

(a)

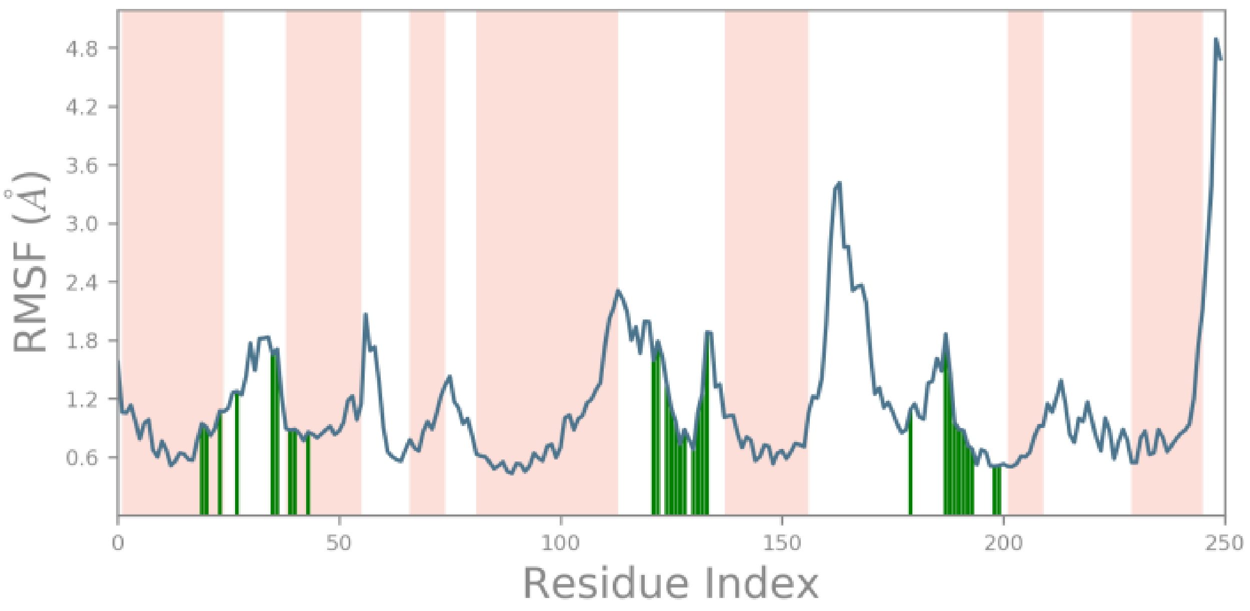

(b)

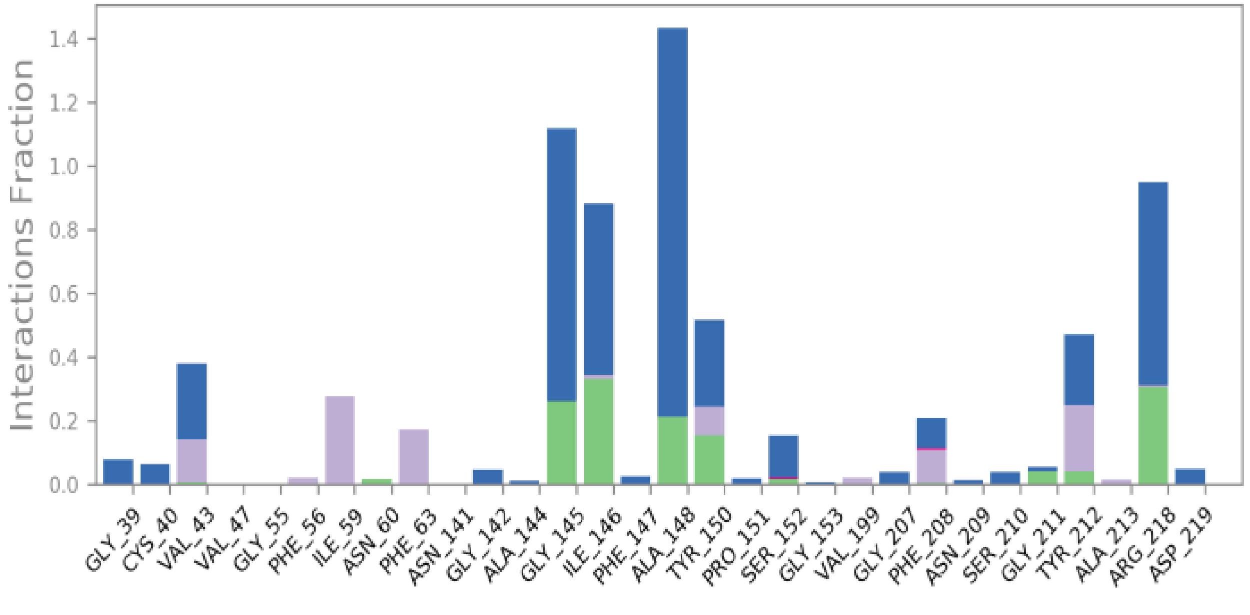

(c)

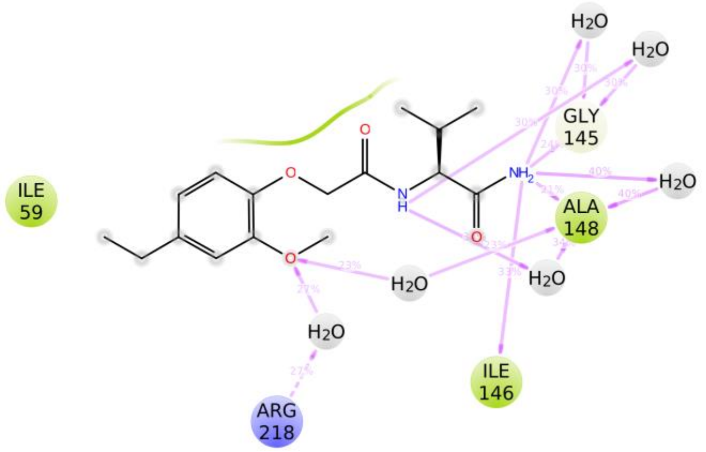
H2O
H2O
GLY
145
NH2
H2O
ILE
59
N
H
O
O
O
ALA
148
O
H2O
H2O
H2O
ILE
146
ARG
218

(d)

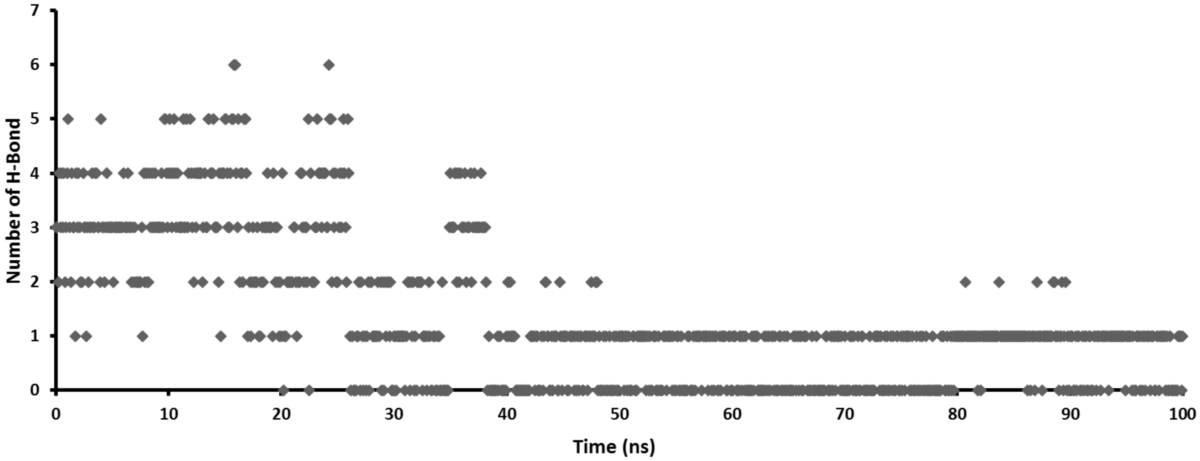
Number of H-Bond
Time (ns)
0
1
2
3
4
5
6
7
0
10
20
30
40
50
60
70
80
90
100

(e)

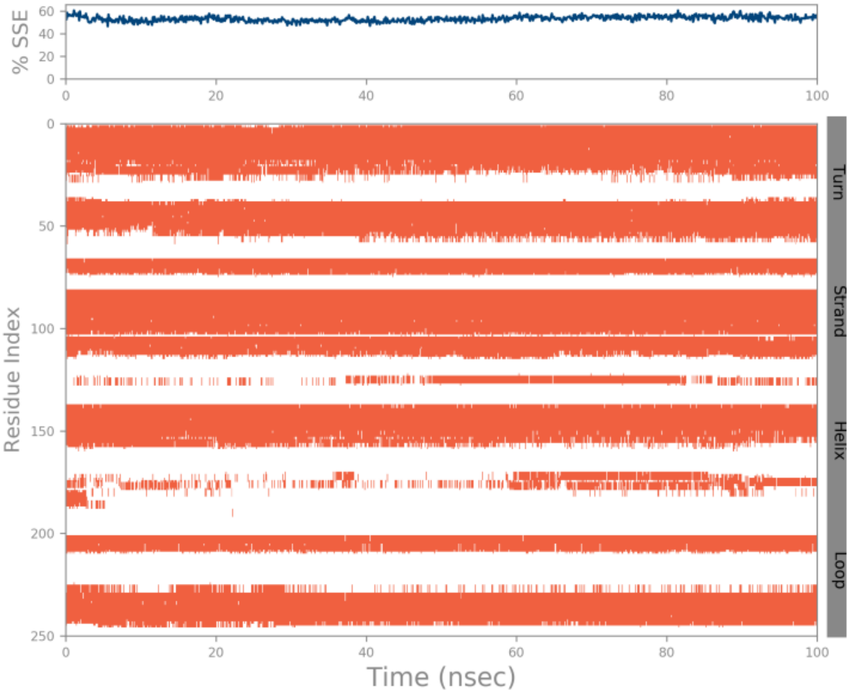
% SSE
Residue Index
Time (nsec)
Turn
Strand
Helix
Loop

Figure S36. (a) Root mean squared fluctuations (RMSFs) of Cα-atoms along with contacts (green-colored vertical bars) of 5633879 in complex with AQP-3; (b) Protein ligand contacts histogram throughout the simulation for 5633879 in complex with AQP-3; (c) Protein-ligand interaction profile for 5633879 in complex with AQP-3; (d) Hydrogen bond interaction observed between protein and 5633879 in different frames throughout the simulation; (e) the secondary structure elements (SSE) for protein conformation during the simulation (% of Total SSE calculated were 54.22).

(a)

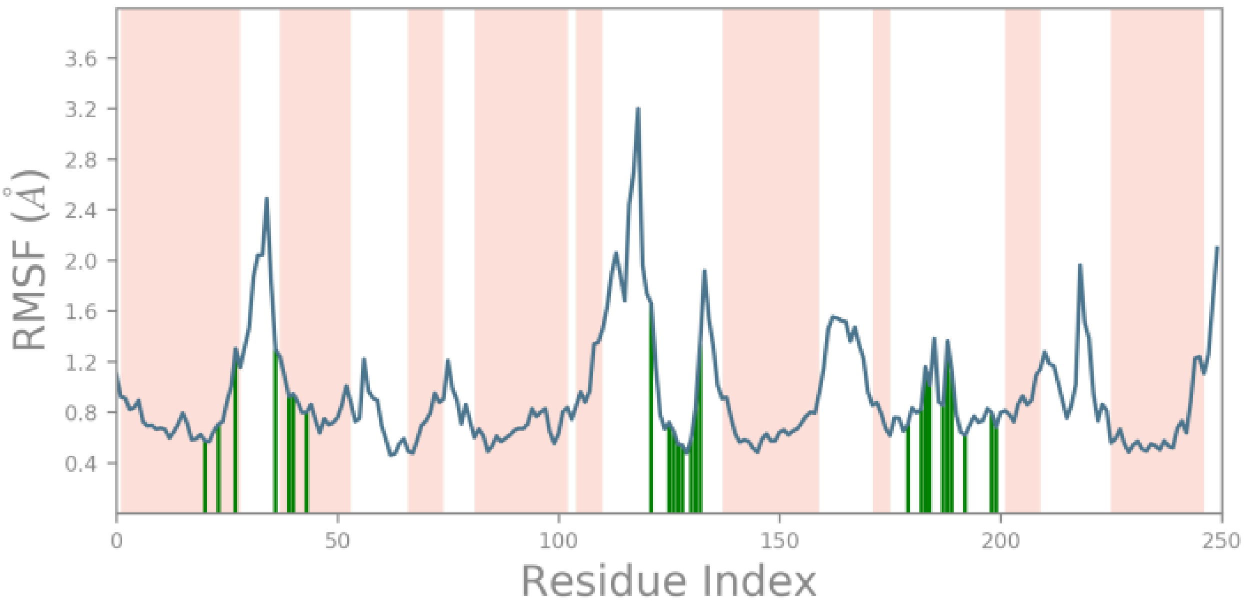

(b)

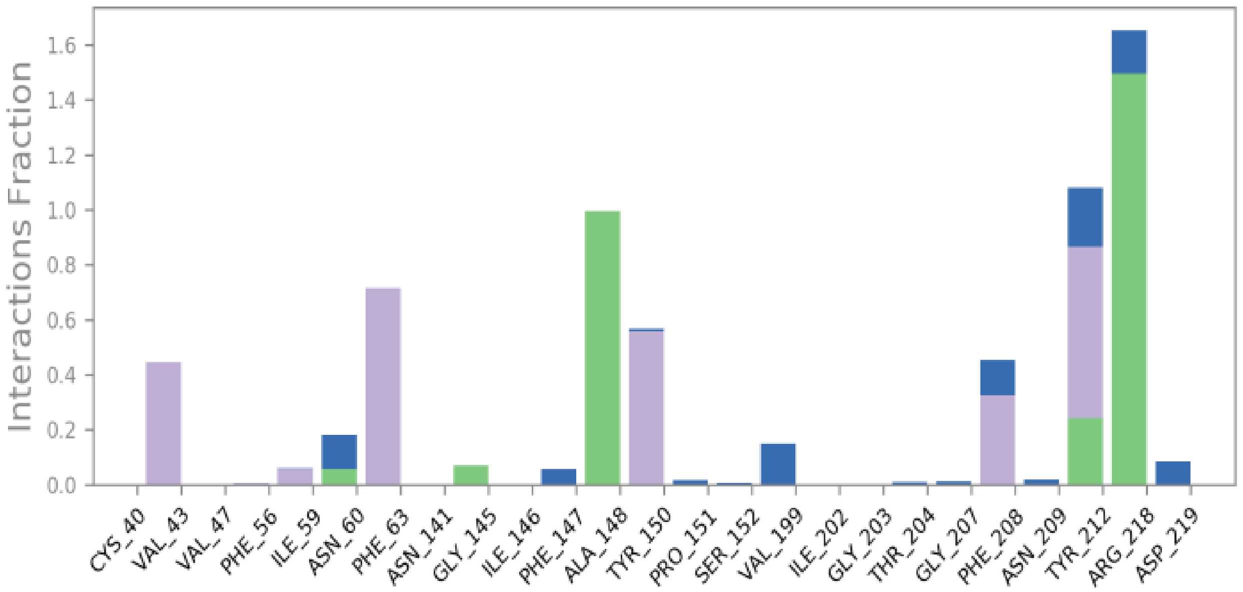

(c)

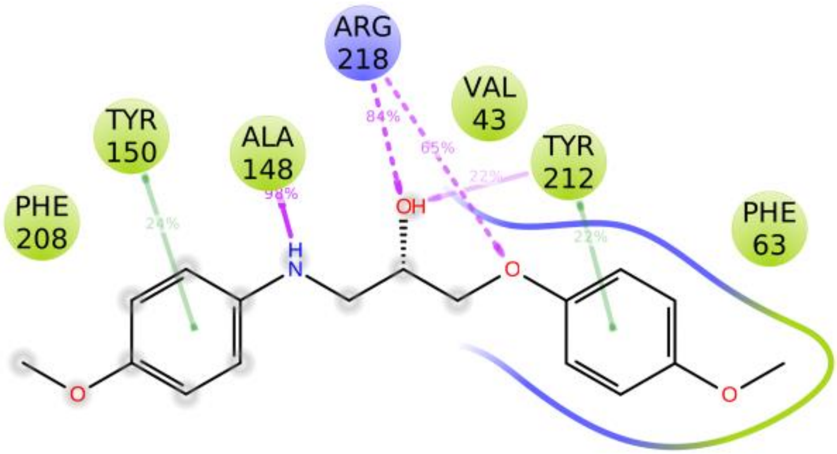
ARG
218
VAL
43
TYR
150
ALA
148
TYR
212
PHE
208
PHE
63
84%
65%
22%
98%
24%
22%
OH
H
N
O
O
O

(d)

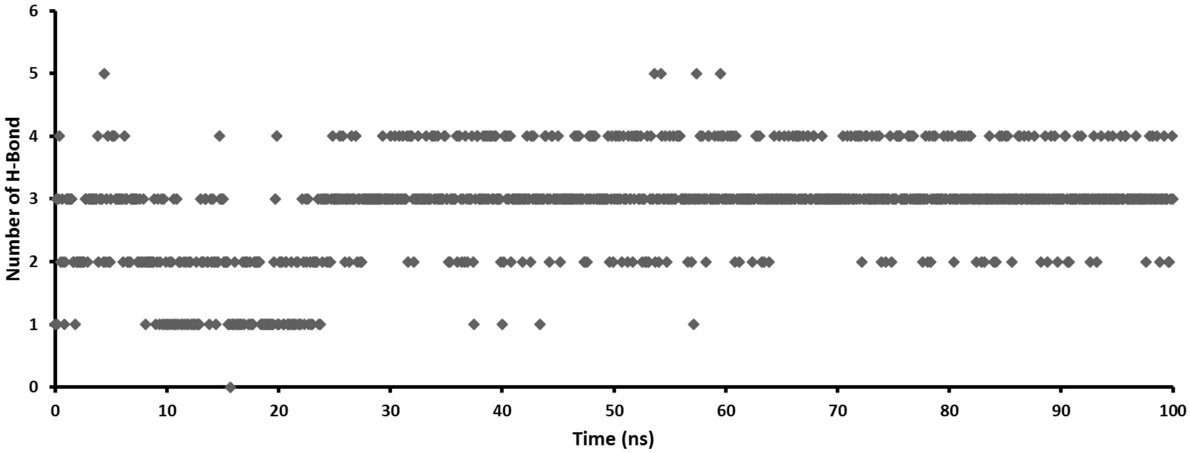
Number of H-Bond
Time (ns)
0
1
2
3
4
5
6
10
20
30
40
50
60
70
80
90
100

(e)

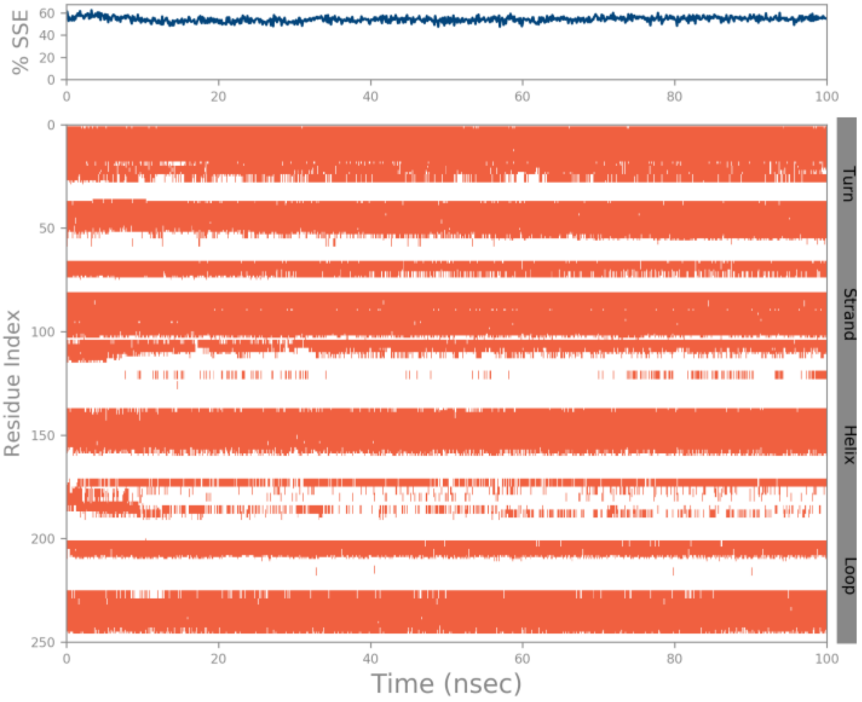
% SSE
Residue Index
Time (nsec)
Turn
Strand
Helix
Loop

Figure S37. (a) Root mean squared fluctuations (RMSFs) of Cα-atoms along with contacts (green-colored vertical bars) of 36994203 in complex with AQP-3; (b) Protein ligand contacts histogram throughout the simulation for 36994203 in complex with AQP-3; (c) Protein-ligand interaction profile for 36994203 in complex with AQP-3; (d) Hydrogen bond interaction observed between protein and 36994203 in different frames throughout the simulation; (e) the secondary structure elements (SSE) for protein conformation during the simulation (% of Total SSE calculated were 53.93).

(a)

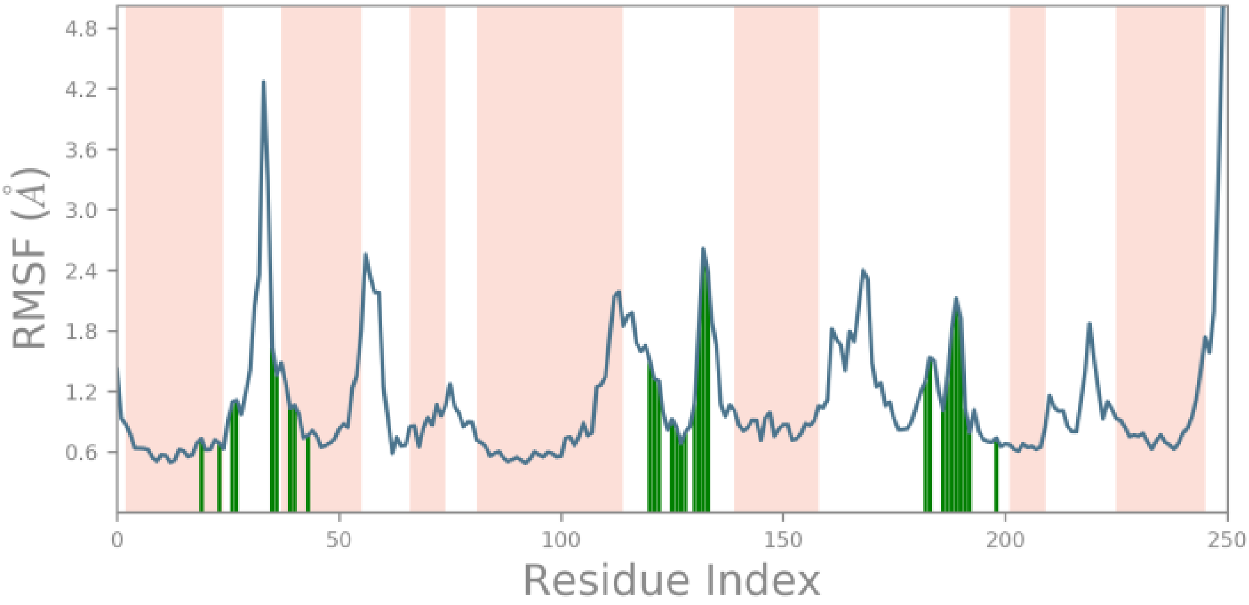

(b)

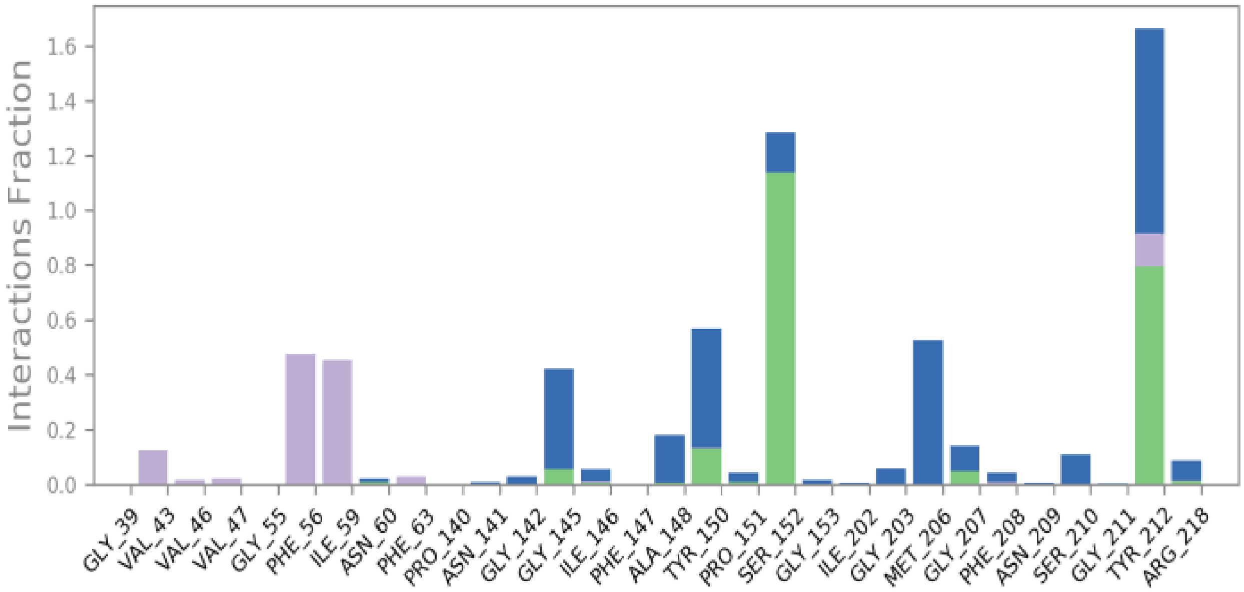

(c)

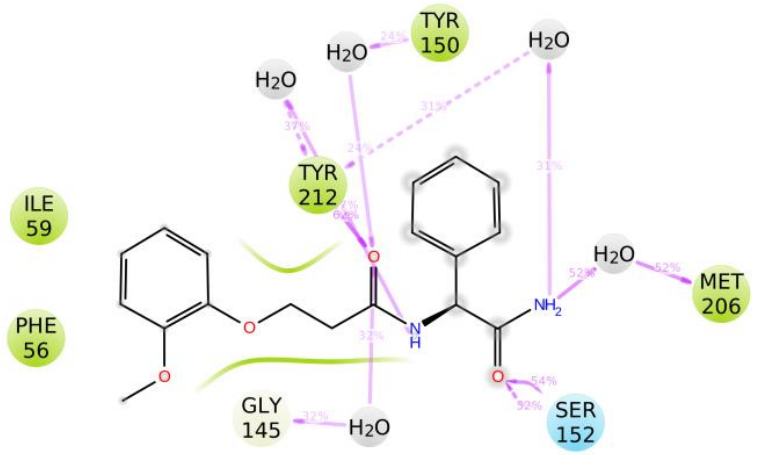
TYR
150
H2O
H2O
H2O
TYR
212
ILE
59
PHE
56
GLY
145
H2O
H2O
MET
206
SER
152

(d)

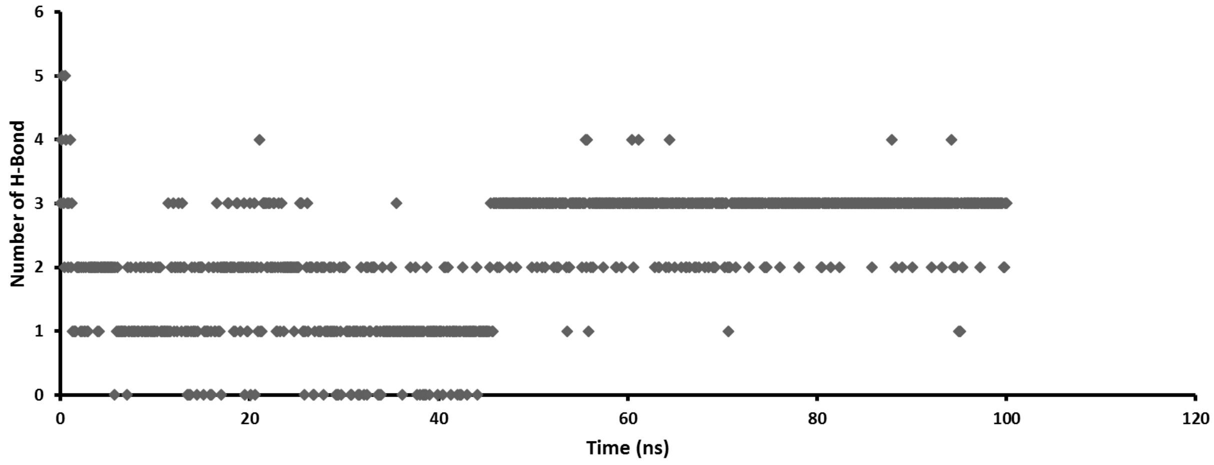
Number of H-Bond
Time (ns)

(e)

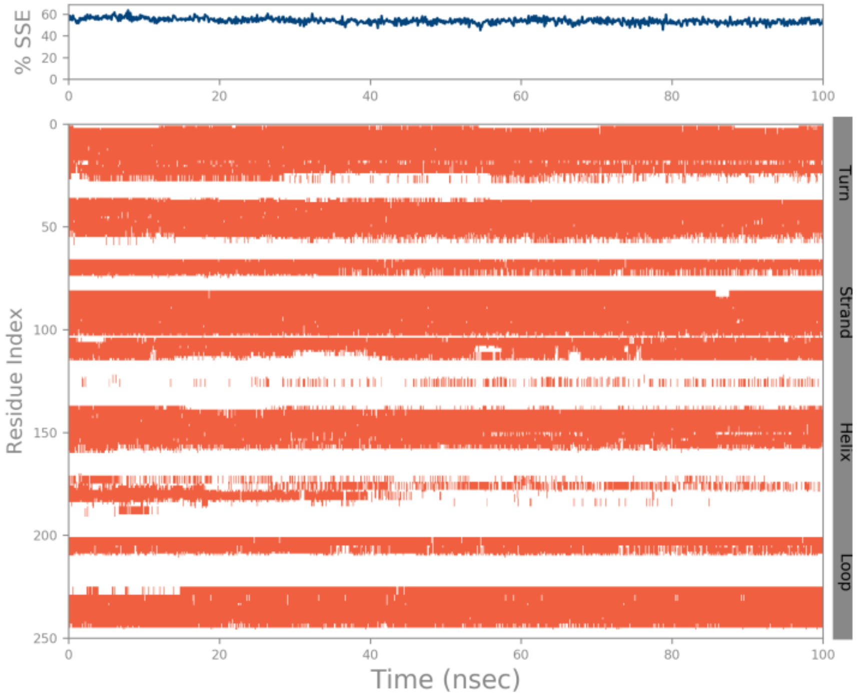
% SSE
Residue Index
Time (nsec)
Turn
Strand
Helix
Loop

Figure S38. (a) Root mean squared fluctuations (RMSFs) of Cα-atoms along with contacts (green-colored vertical bars) of 16694164 in complex with AQP-3; (b) Protein ligand contacts histogram throughout the simulation for 16694164 in complex with AQP-3; (c) Protein-ligand interaction profile for 16694164 in complex with AQP-3; (d) Hydrogen bond interaction observed between protein and 16694164 in different frames throughout the simulation; (e) the secondary structure elements (SSE) for protein conformation during the simulation (% of Total SSE calculated were 52.16).

(a)

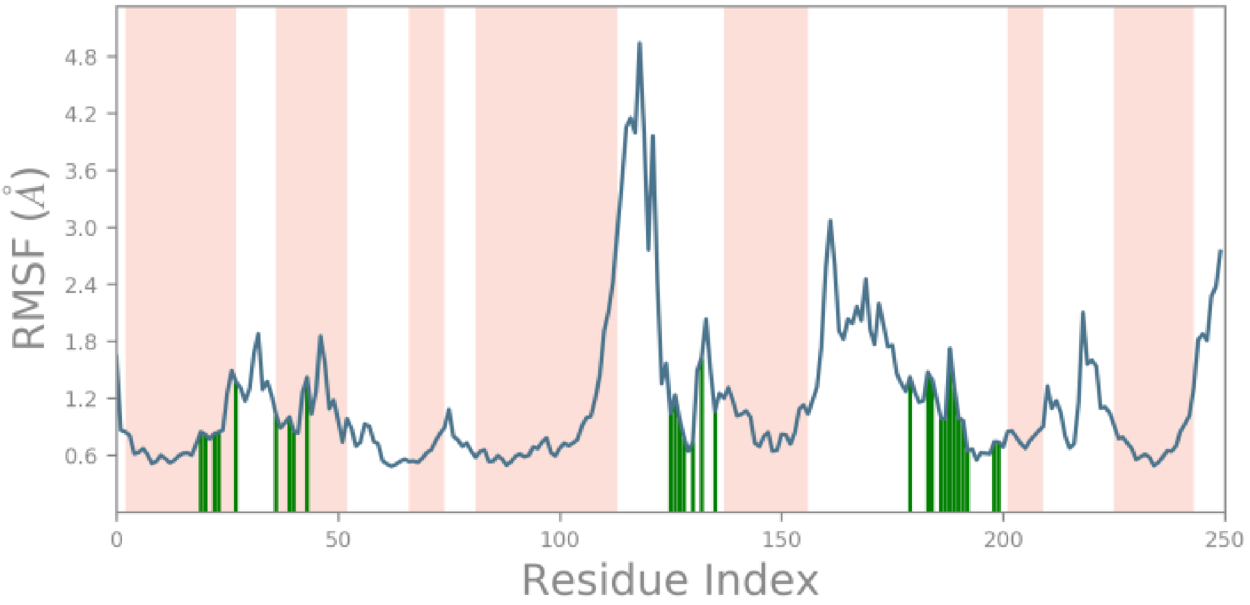

(b)

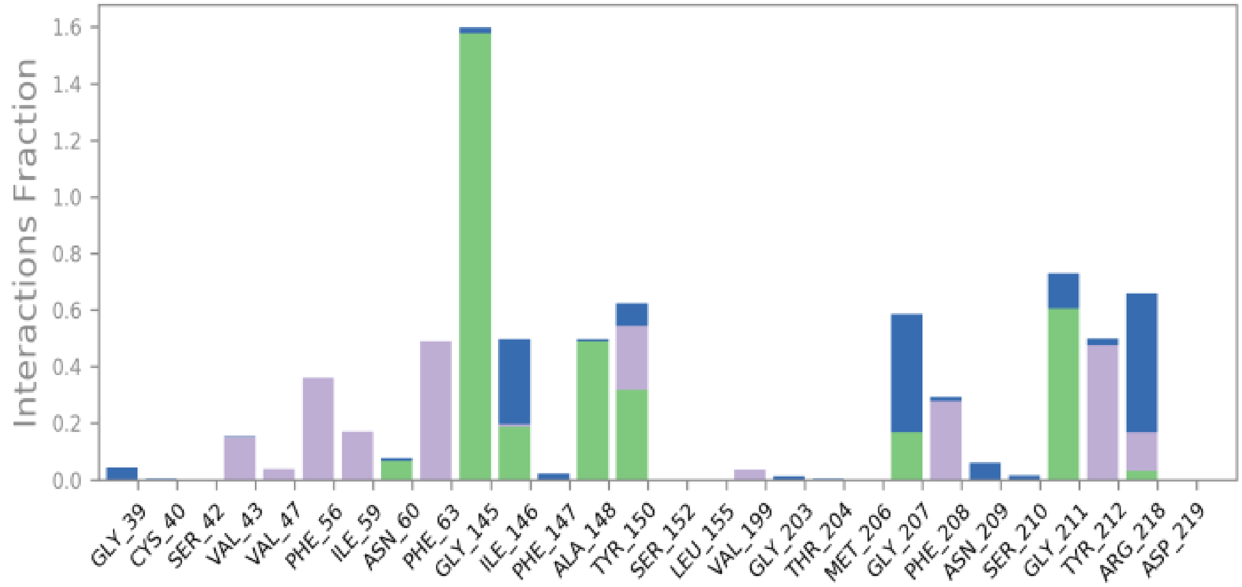

(c)

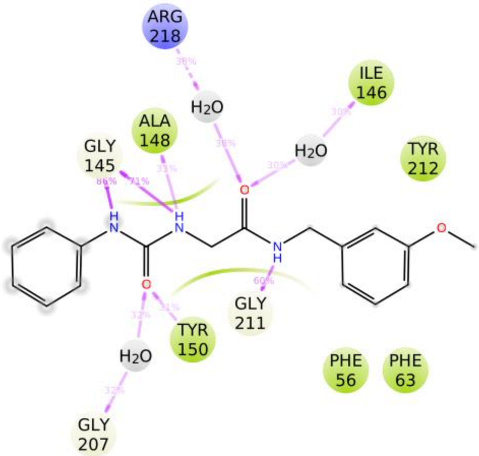
ARG 218
ILE 146
H2O
ALA 148
GLY 145
H2O
TYR 212
GLY 211
TYR 150
H2O
PHE 56
PHE 63
GLY 207

(d)

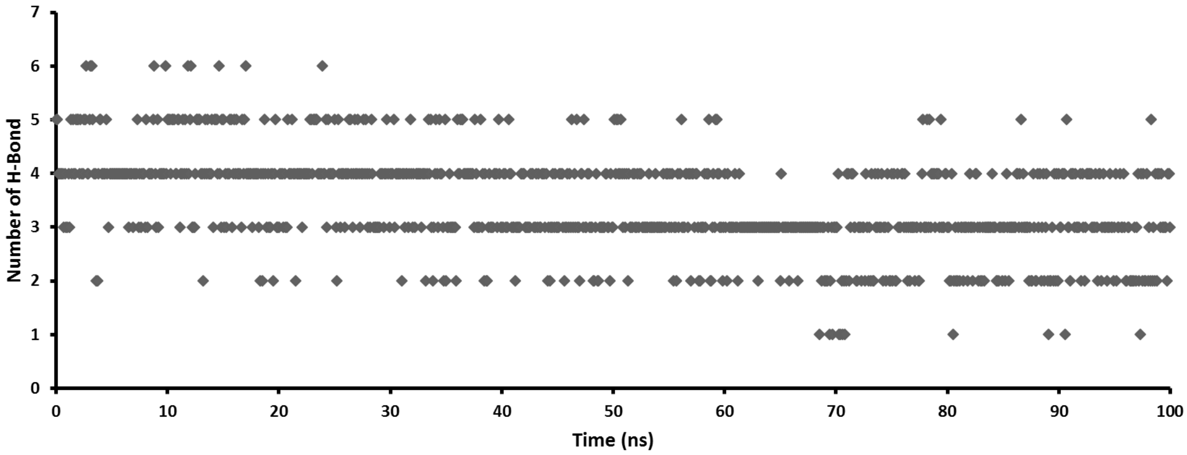
Number of H-Bond
Time (ns)
0
1
2
3
4
5
6
7
10
20
30
40
50
60
70
80
90
100

(e)

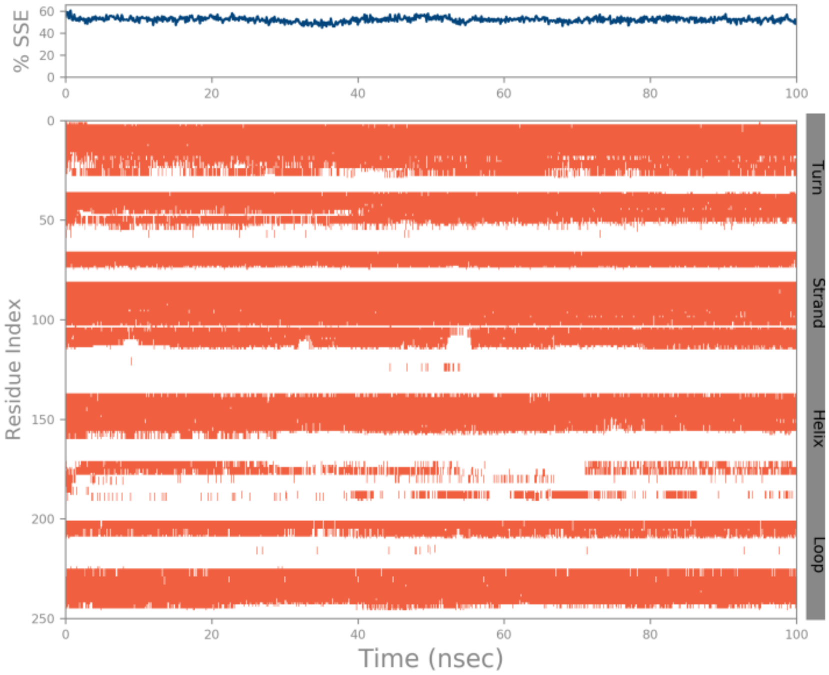
% SSE
Residue Index
Time (nsec)
Turn
Strand
Helix
Loop

Figure S39. (a) Root mean squared fluctuations (RMSFs) of Cα-atoms along with contacts (green-colored vertical bars) of 13477729 in complex with AQP-3; (b) Protein ligand contacts histogram throughout the simulation for 13477729 in complex with AQP-3; (c) Protein-ligand interaction profile for 13477729 in complex with AQP-3; (d) Hydrogen bond interaction observed between protein and 13477729 in different frames throughout the simulation; (e) the secondary structure elements (SSE) for protein conformation during the simulation (% of Total SSE calculated were 54.68).

(a)

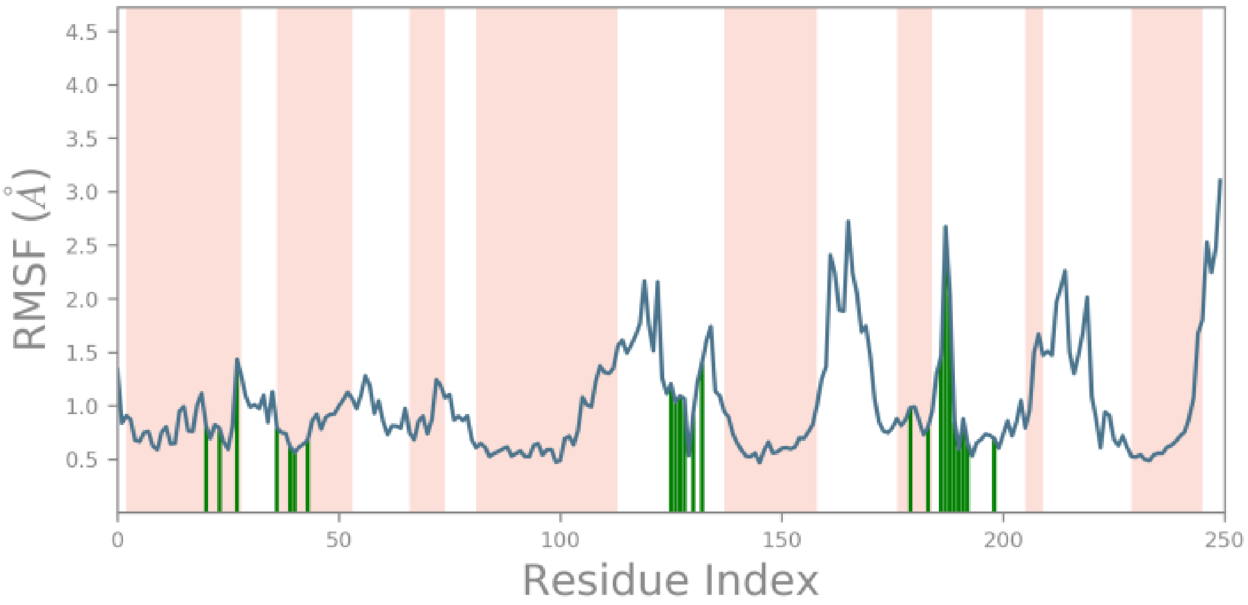

(b)

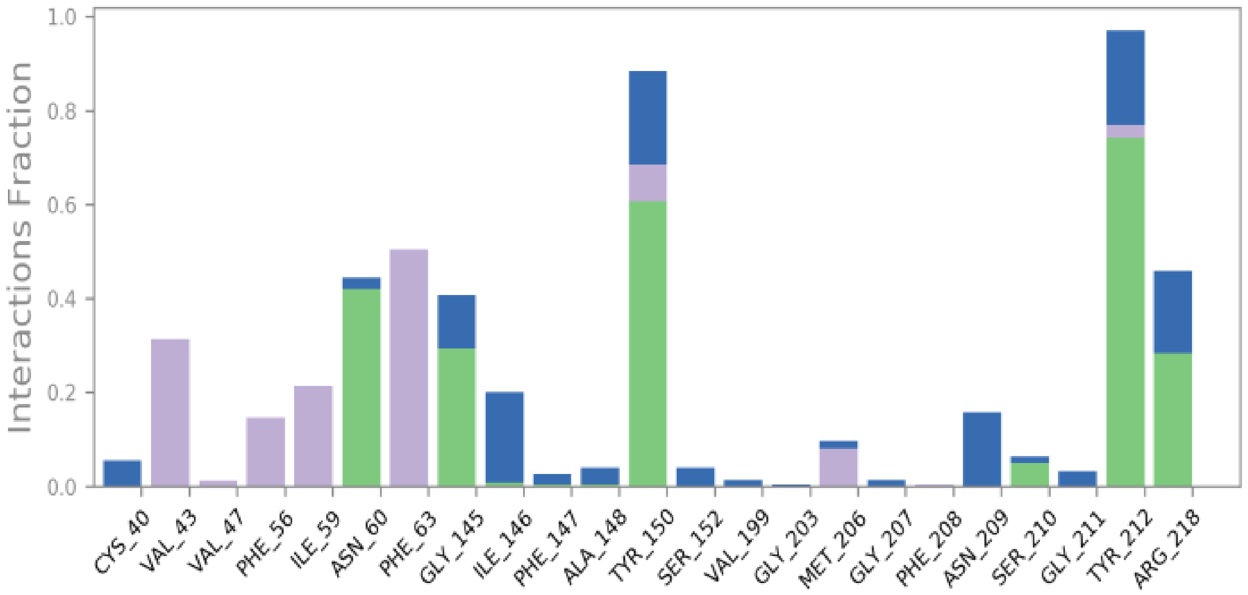

(c)

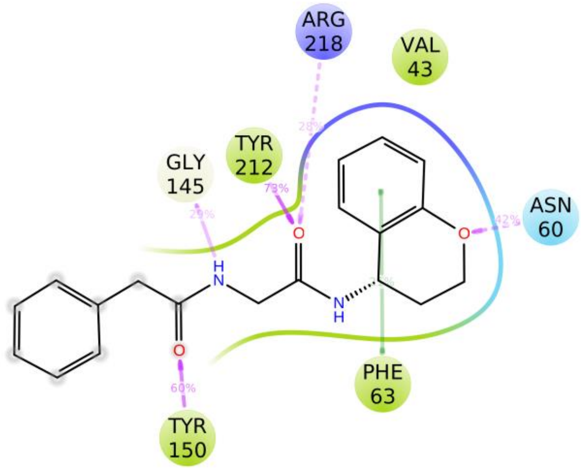

ARG 218
VAL 43
TYR 212
GLY 145
ASN 60
PHE 63
TYR 150

(d)

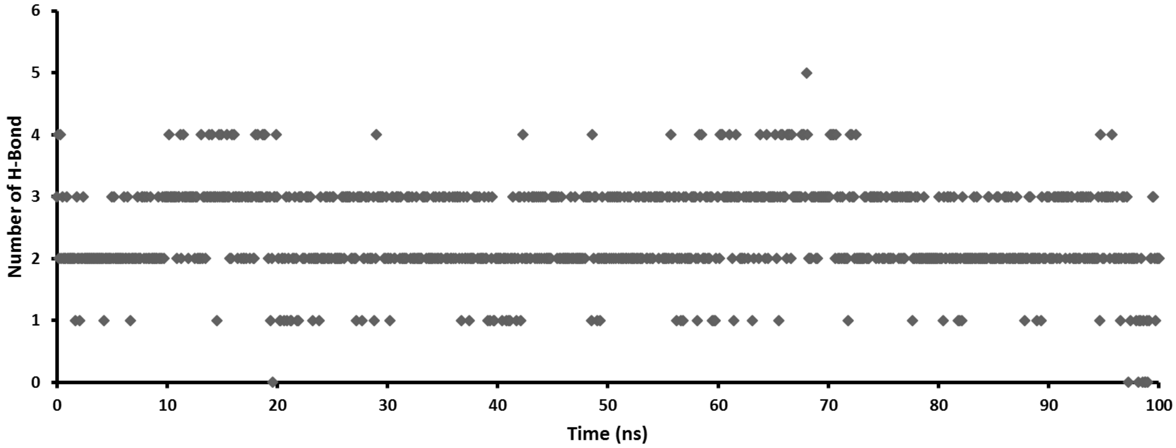

Number of H-Bond
Time (ns)

(e)

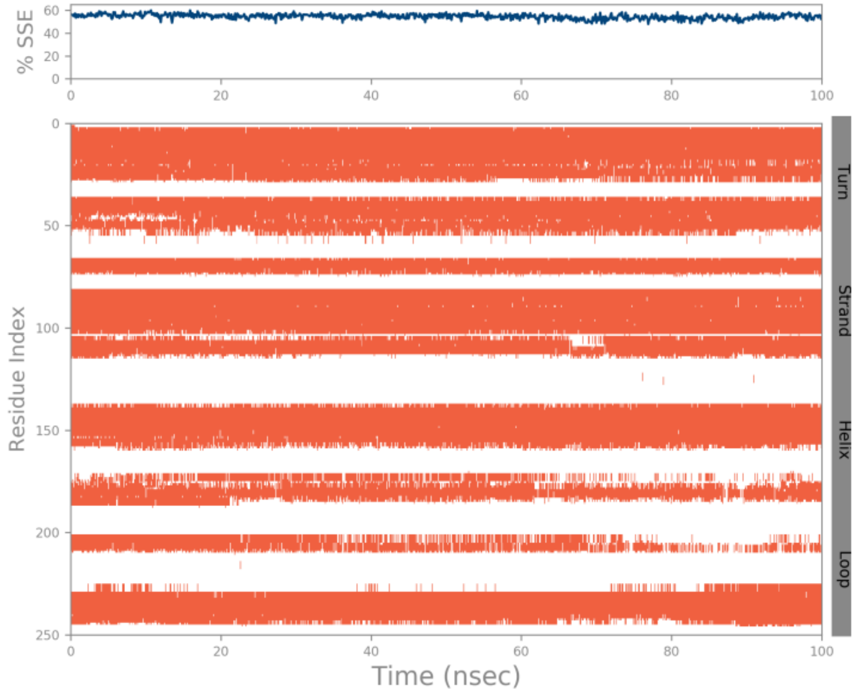

% SSE
Residue Index
Time (nsec)
Turn
Strand
Helix
Loop

Figure S40. (a) Root mean squared fluctuations (RMSFs) of Cα-atoms along with contacts (green-colored vertical bars) of 36657947 in complex with AQP-3; (b) Protein ligand contacts histogram throughout the simulation for 36657947 in complex with AQP-3; (c) Protein-ligand interaction profile for 36657947 in complex with AQP-3; (d) Hydrogen bond interaction observed between protein and 36657947 in different frames throughout the simulation; (e) the secondary structure elements (SSE) for protein conformation during the simulation (% of Total SSE calculated were 53.50).

(a)

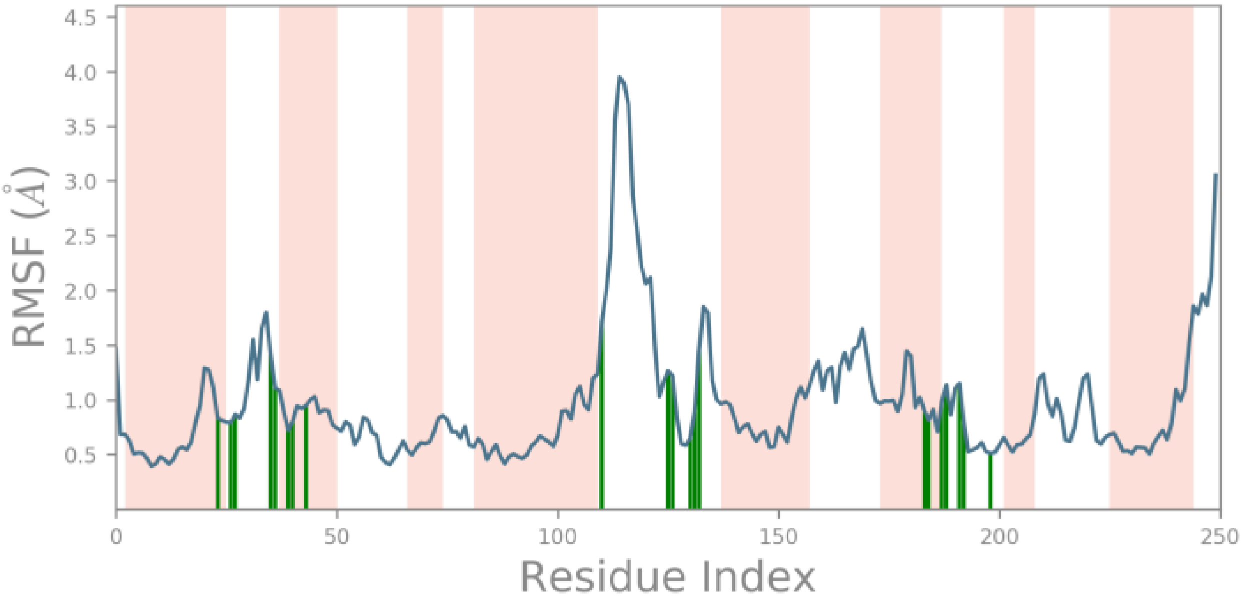

(b)

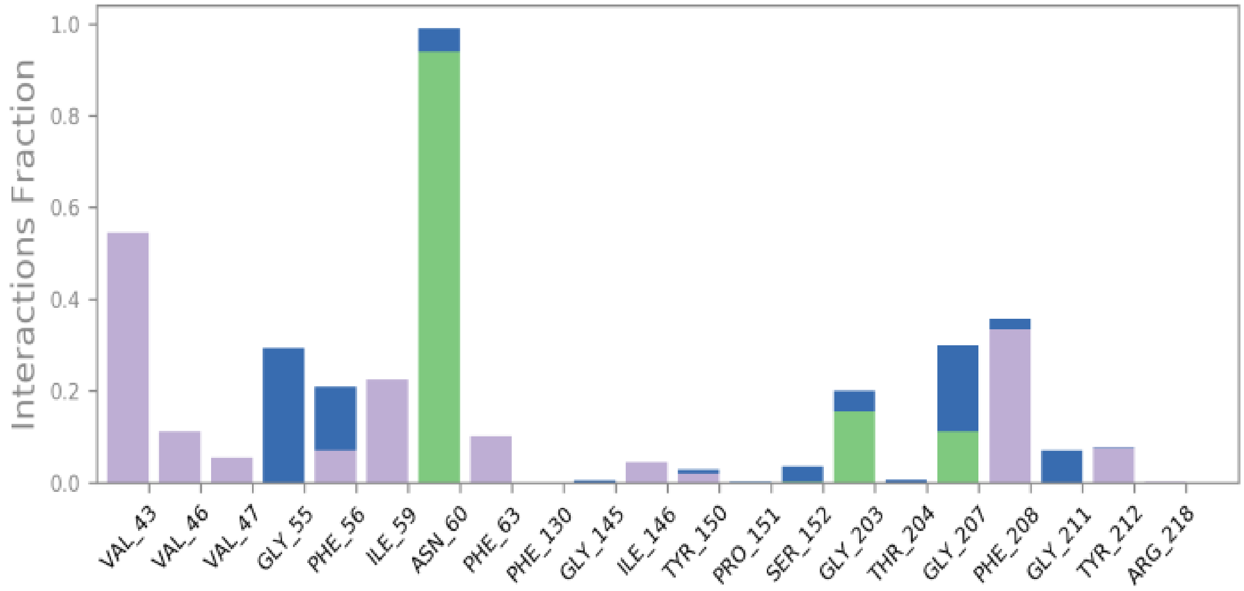

(c)

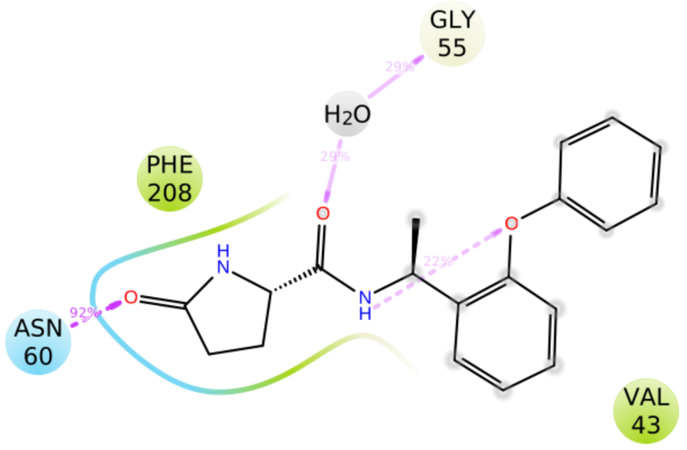
GLY
55
29%
H_2O
29%
PHE
208
O
H
N
O
N
H
22%
O
92%
ASN
60
VAL
43

(d)

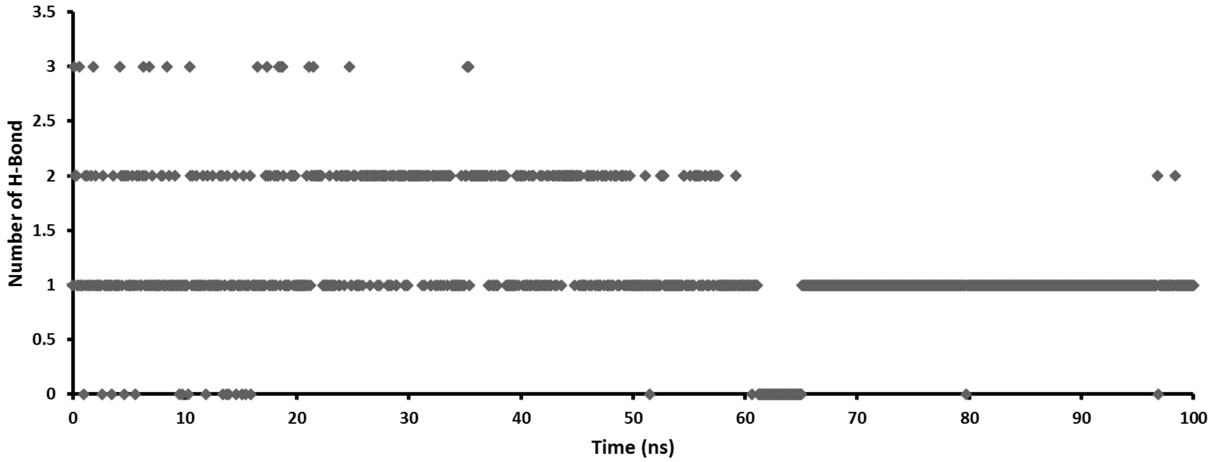
Number of H-Bond
3.5
3
2.5
2
1.5
1
0.5
0
0
10
20
30
40
50
60
70
80
90
100
Time (ns)

(e)

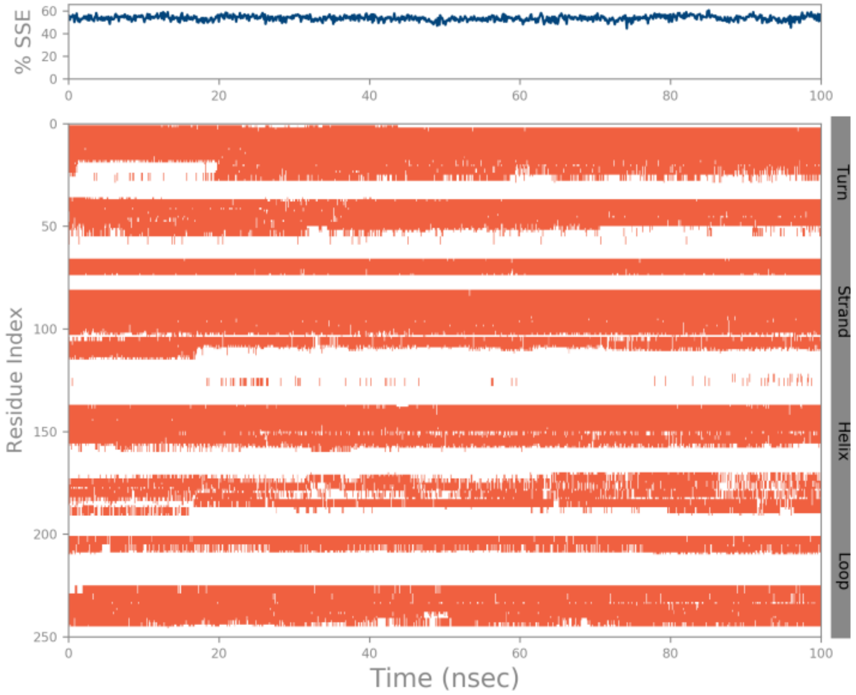
% SSE
60
40
20
0
0
20
40
60
80
100
Residue Index
0
50
100
150
200
250
Turn
Strand
Helix
Loop
Time (nsec)

Figure S41. (a) Root mean squared fluctuations (RMSFs) of Cα-atoms along with contacts (green-colored vertical bars) of 36716128 in complex with AQP-3; (b) Protein ligand contacts histogram throughout the simulation for 36716128 in complex with AQP-3; (c) Protein-ligand interaction profile for 36716128 in complex with AQP-3; (d) Hydrogen bond interaction observed between protein and 36716128 in different frames throughout the simulation; (e) the secondary structure elements (SSE) for protein conformation during the simulation (% of Total SSE calculated were 56.02).

(a)

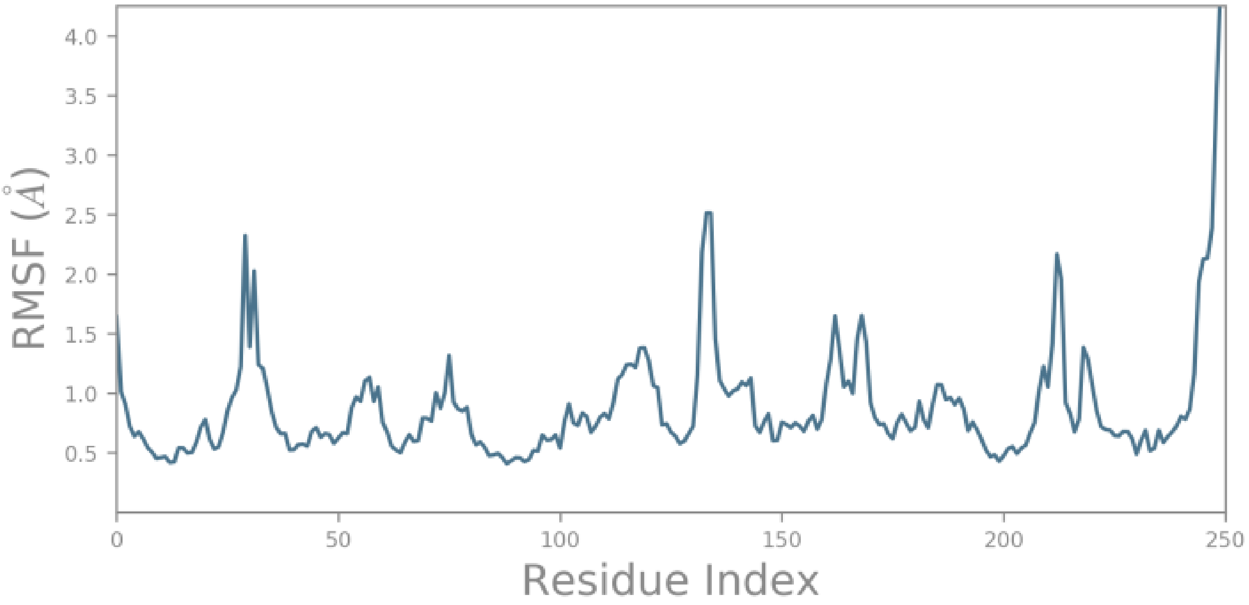

(b)

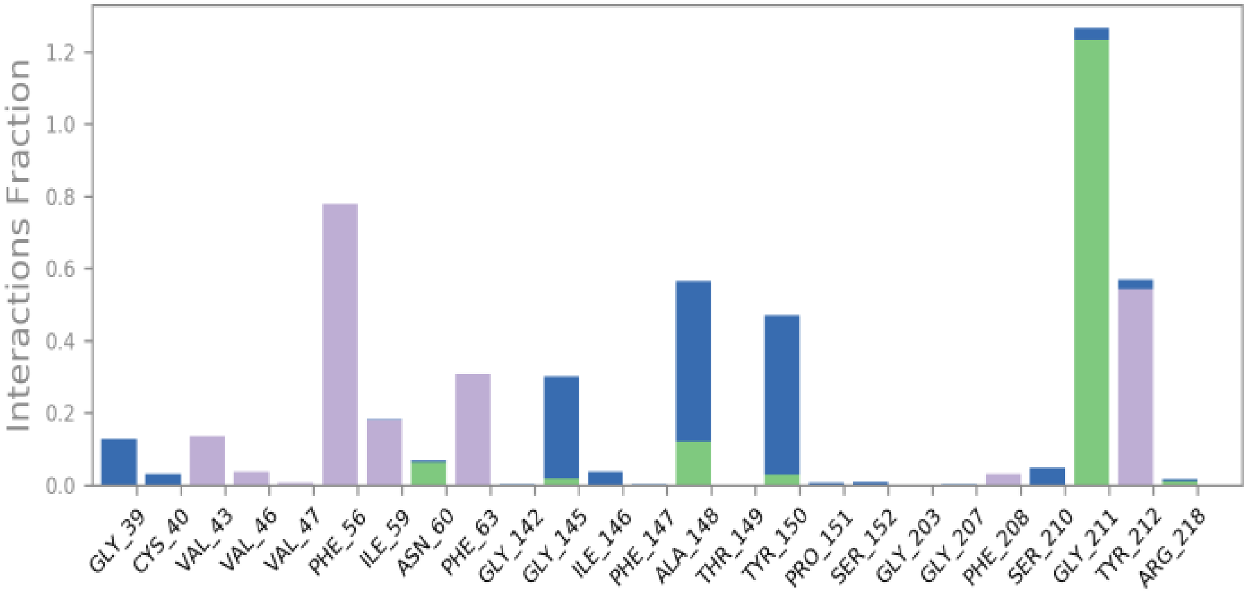

(c)

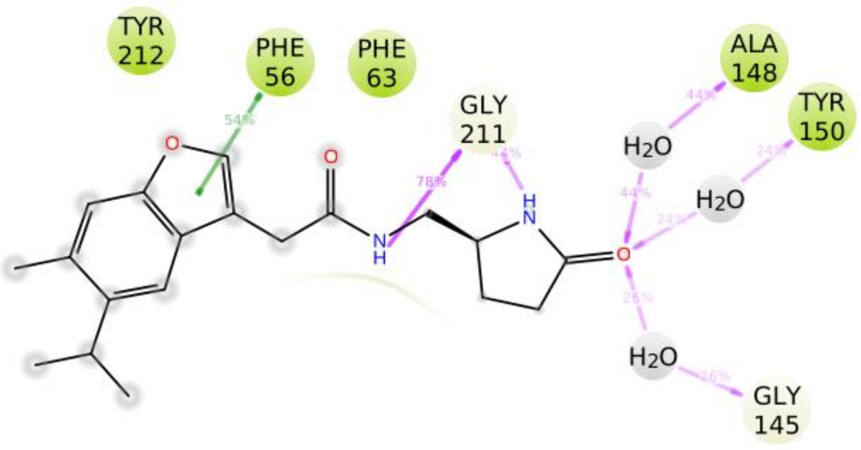

TYR 212
PHE 56
PHE 63
GLY 211
ALA 148
TYR 150
H2O
H2O
H2O
GLY 145
54%
78%
44%
44%
44%
24%
24%
26%
26%
O
O
N H
H N
O
O

(d)

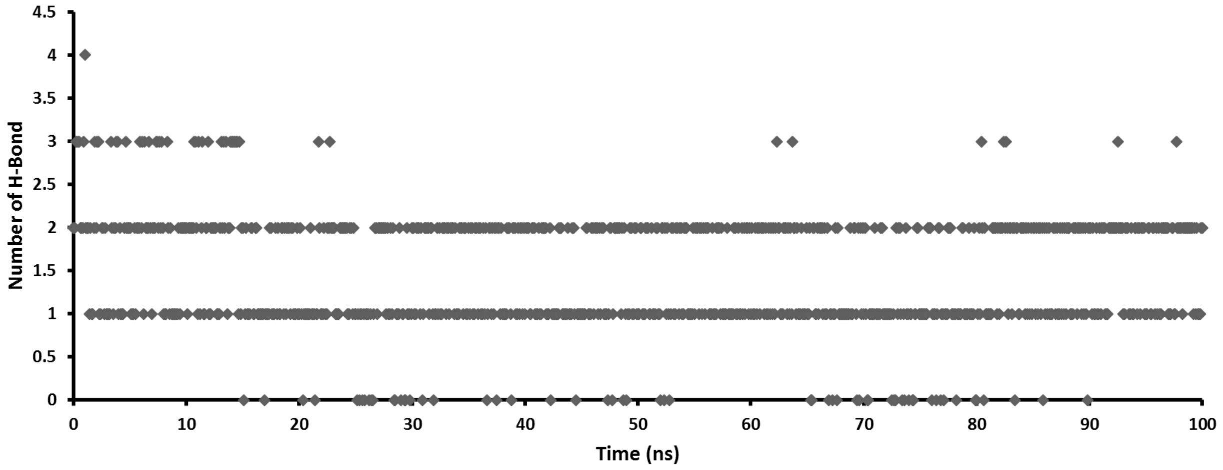

Number of H-Bond
Time (ns)
0
0.5
1
1.5
2
2.5
3
3.5
4
4.5
0
10
20
30
40
50
60
70
80
90
100

(e)

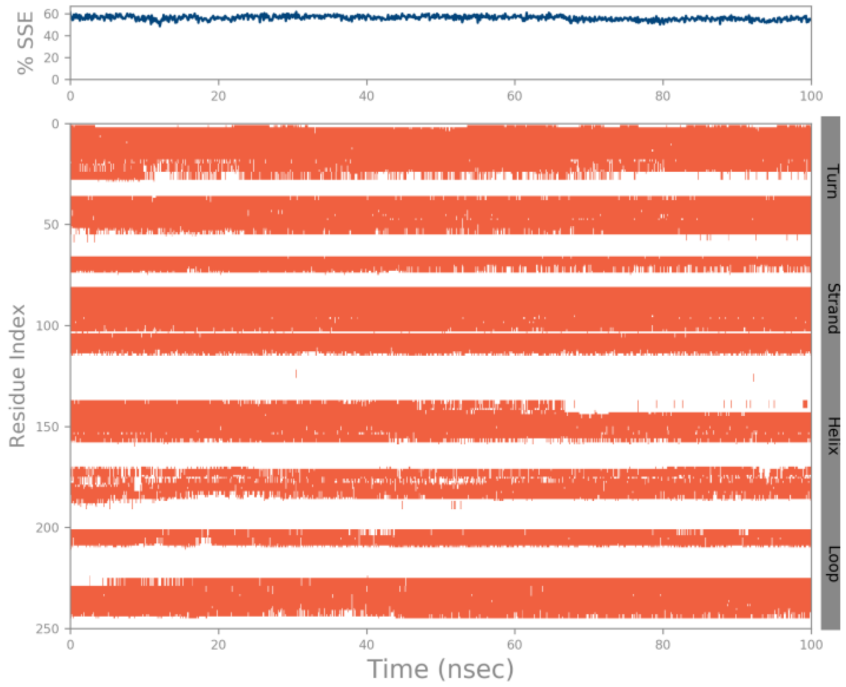

% SSE
Residue Index
Time (nsec)
Turn
Strand
Helix
Loop
0
20
40
60
80
100
50
150
200
250

Figure S42. (a) Root mean squared fluctuations (RMSFs) of Cα-atoms along with contacts (green-colored vertical bars) of 3325122 in complex with AQP-3; (b) Protein ligand contacts histogram throughout the simulation for 3325122 in complex with AQP-3; (c) Protein-ligand interaction profile for 3325122 in complex with AQP-3; (d) Hydrogen bond interaction observed between protein and 3325122 in different frames throughout the simulation; (e) the secondary structure elements (SSE) for protein conformation during the simulation (% of Total SSE calculated were 54.24).

(a)

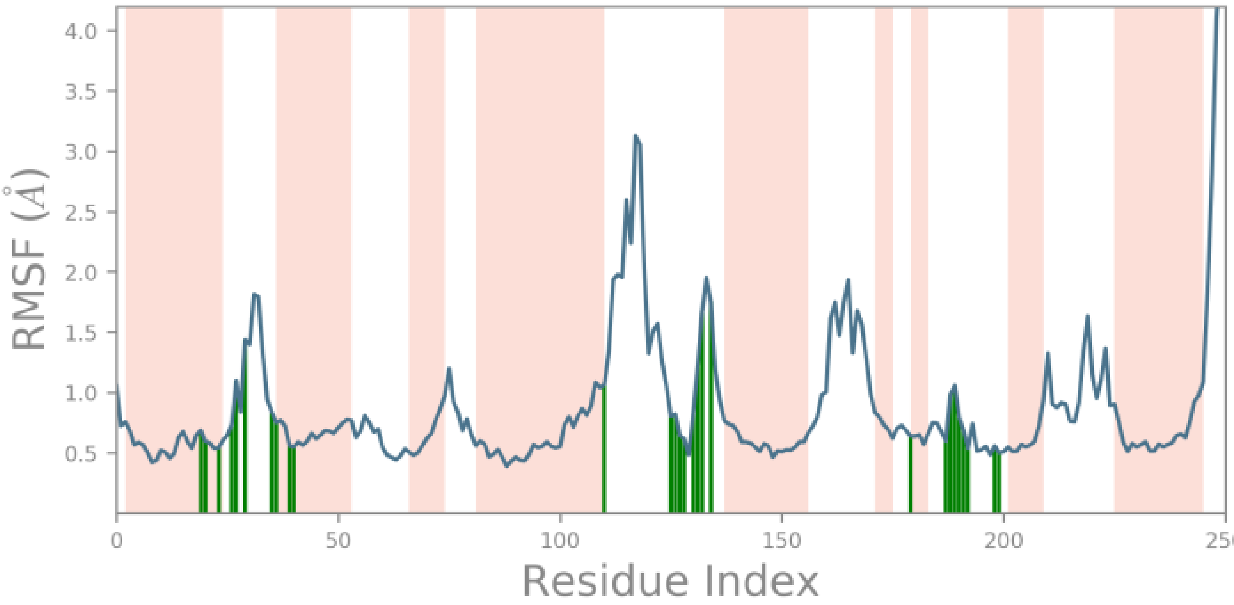

(b)

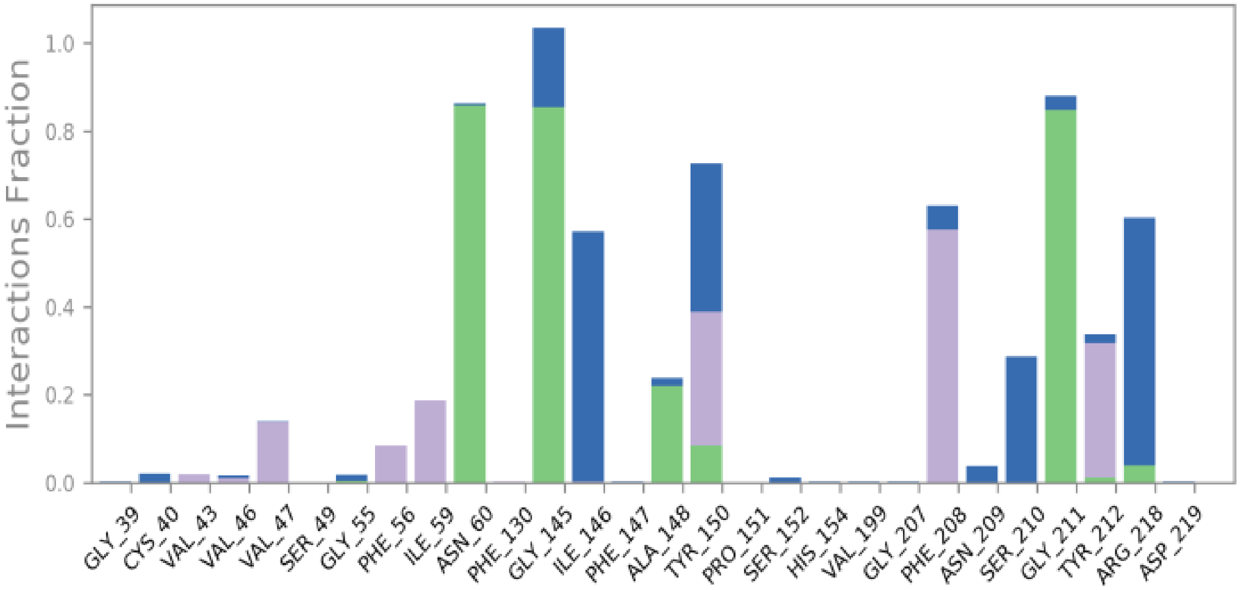

(c)

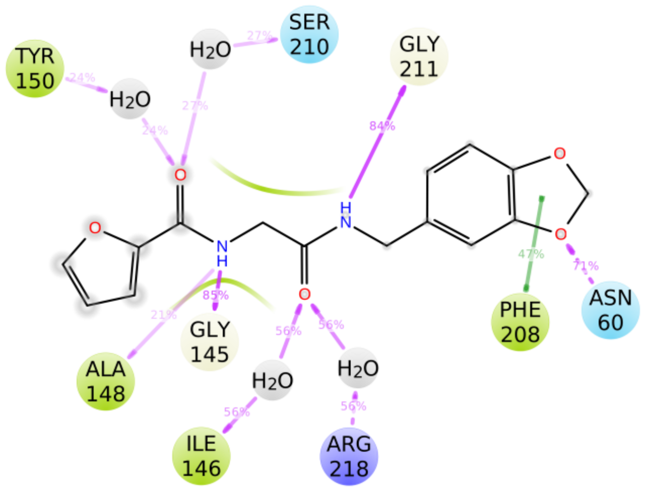

TYR 150
H2O
SER 210
GLY 211
H2O
O
O
N H
H N
O
O
GLY 145
ALA 148
H2O
H2O
ILE 146
ARG 218
PHE 208
ASN 60

(d)

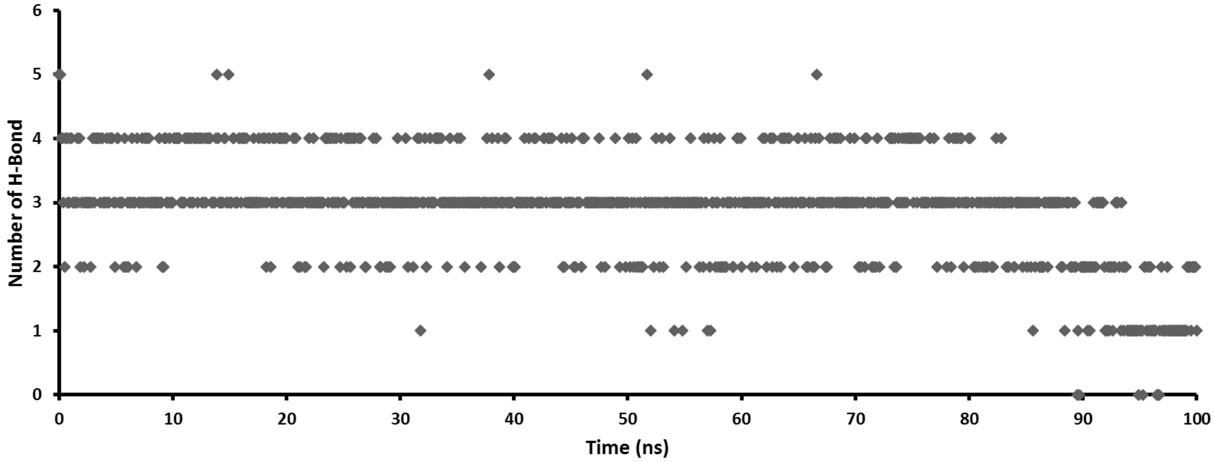

Number of H-Bond
Time (ns)

(e)

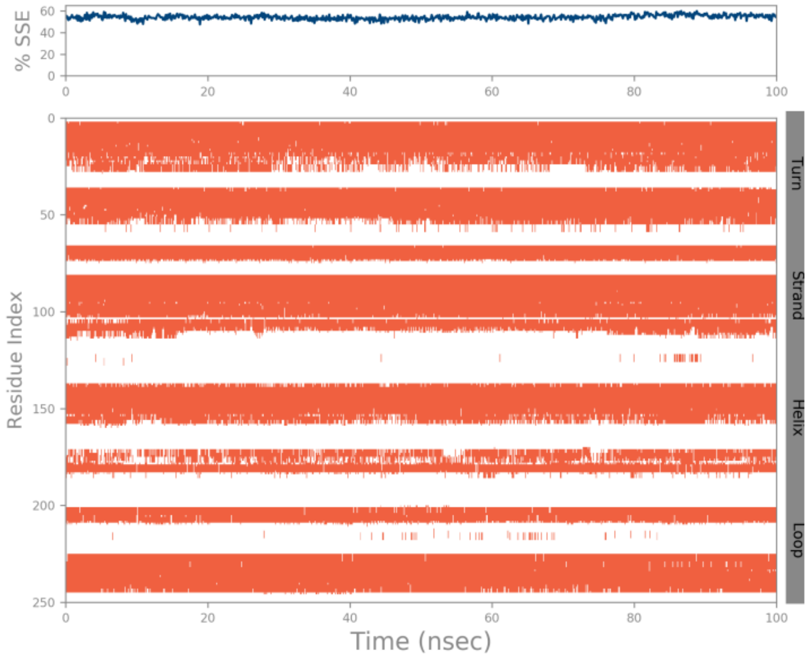

% SSE
Residue Index
Time (nsec)
Turn
Strand
Helix
Loop

Figure S43. (a) Root mean squared fluctuations (RMSFs) of Cα-atoms along with contacts (green-colored vertical bars) of 42888719 in complex with AQP-3; (b) Protein ligand contacts histogram throughout the simulation for 42888719 in complex with AQP-3; (c) Protein-ligand interaction profile for 42888719 in complex with AQP-3; (d) Hydrogen bond interaction observed between protein and 42888719 in different frames throughout the simulation; (e) the secondary structure elements (SSE) for protein conformation during the simulation (% of Total SSE calculated were 53.56).

(a)

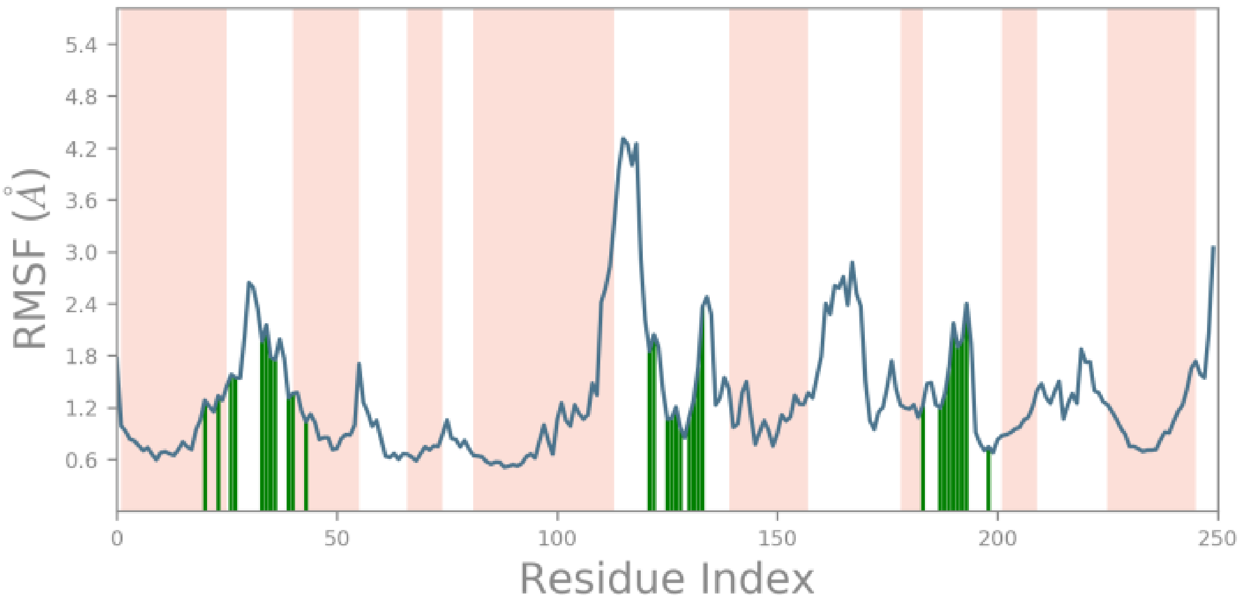

(b)

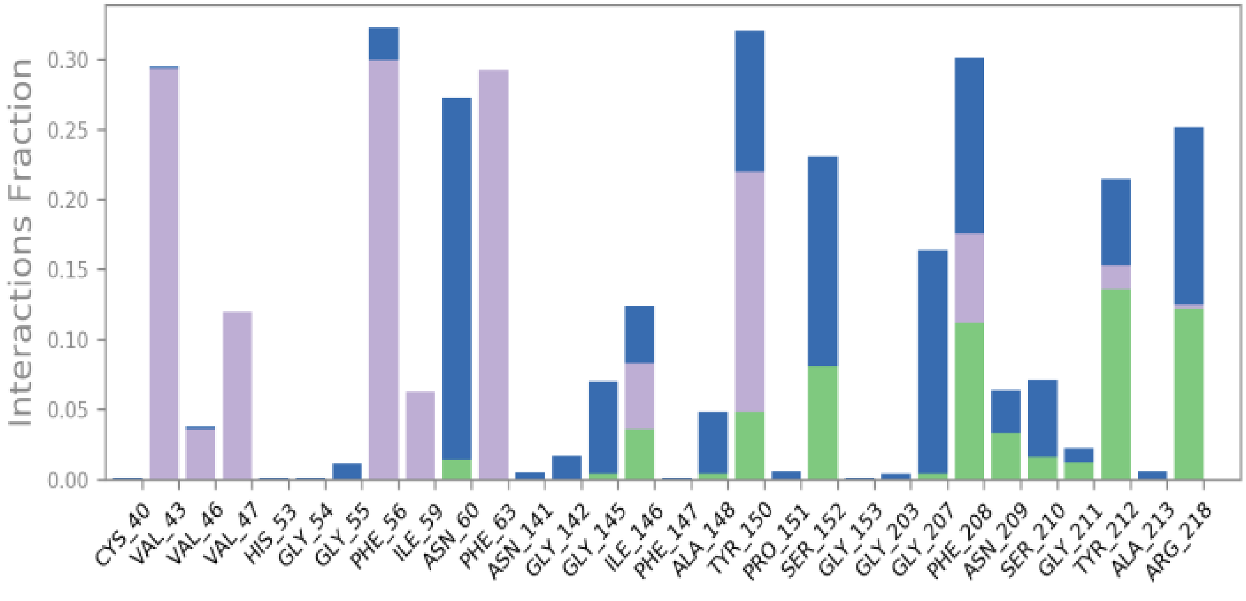

(c)

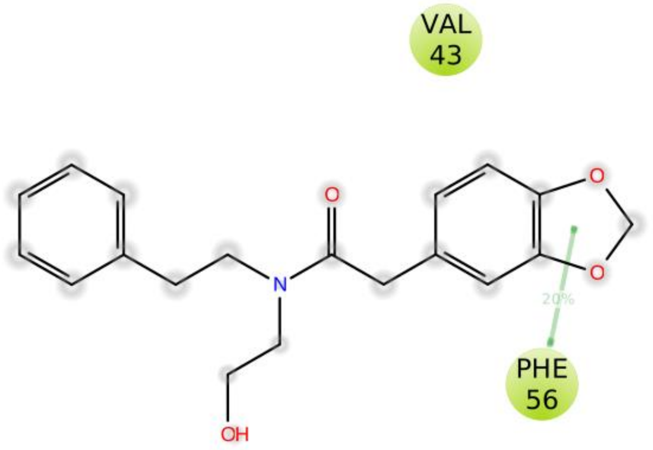
VAL
43
O
O
O
N
OH
20%
PHE
56

(d)

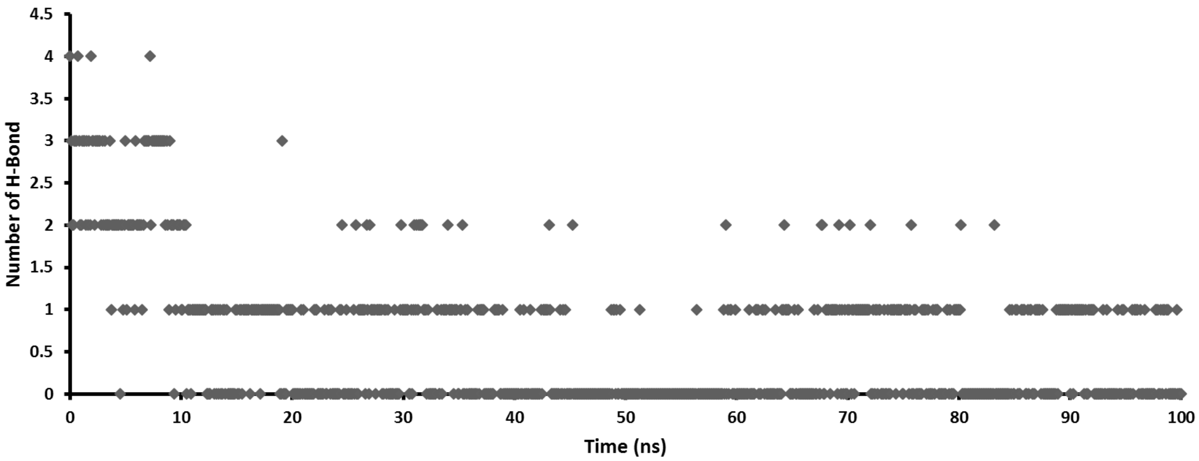
4.5
4
3.5
3
2.5
2
1.5
1
0.5
0
Number of H-Bond
0
10
20
30
40
50
60
70
80
90
100
Time (ns)

(e)

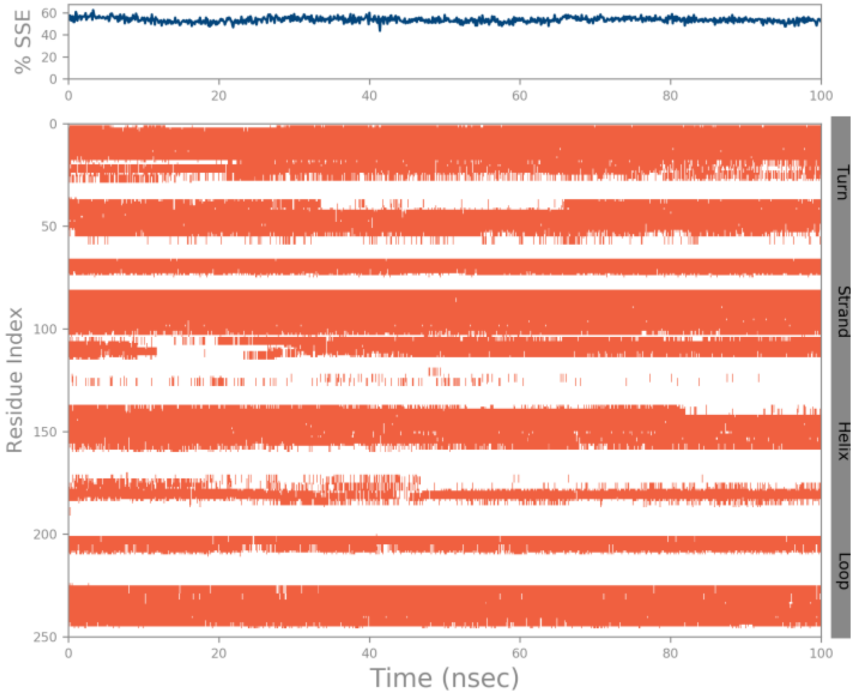
% SSE
60
40
20
0
20
40
60
80
100
Residue Index
50
100
150
200
250
Turn
Strand
Helix
Loop
Time (nsec)

Figure S44. (a) Root mean squared fluctuations (RMSFs) of Cα-atoms along with contacts (green-colored vertical bars) of IP6 in complex with AQP-3; (b) Protein ligand contacts histogram throughout the simulation for IP6 in complex with AQP-3; (c) Protein-ligand interaction profile for IP6 in complex with AQP-3; (d) Hydrogen bond interaction observed between protein and IP6 in different frames throughout the simulation; (e) the secondary structure elements (SSE) for protein conformation during the simulation (% of Total SSE calculated were 57.15).

(a)

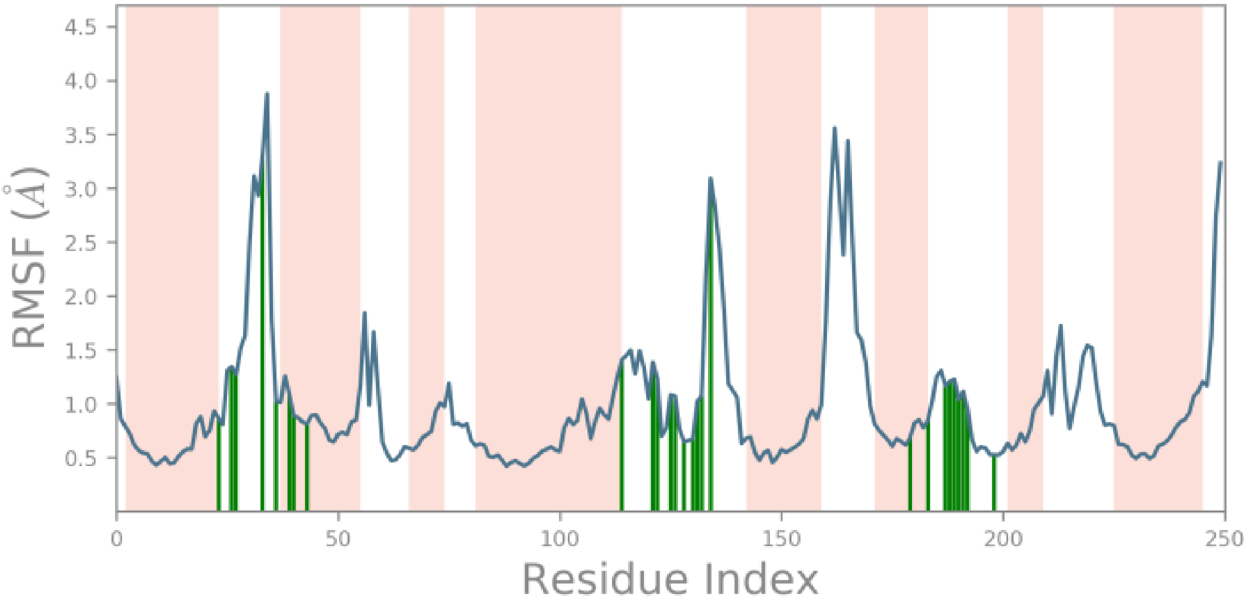

(b)

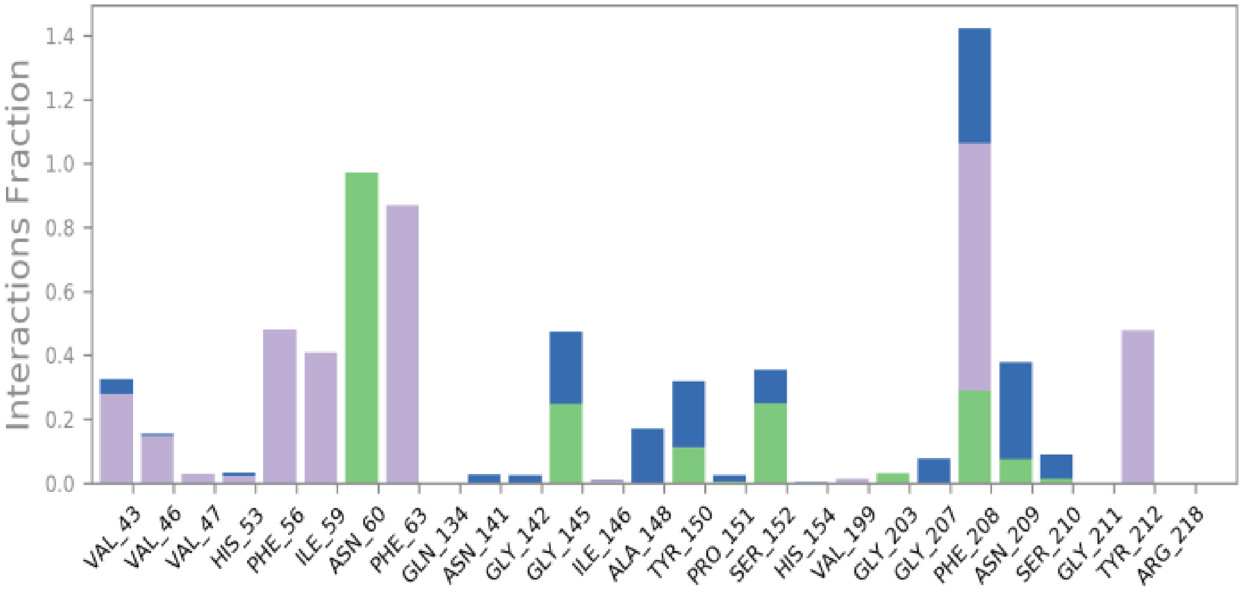

(c)

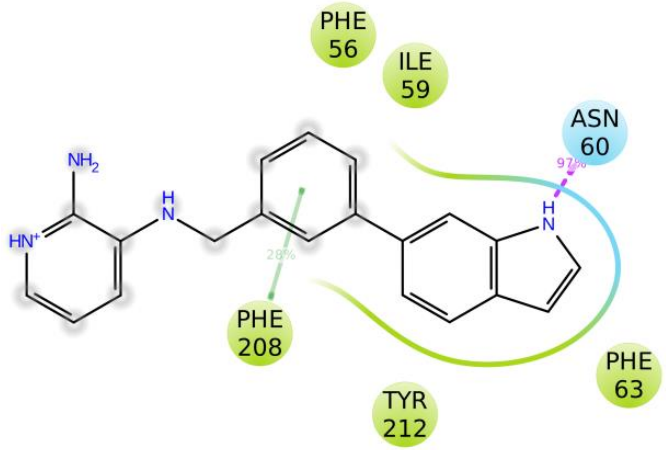
PHE
56
ILE
59
ASN
60
97%
NH2
HN+
H
N
28%
PHE
208
PHE
63
TYR
212

(d)

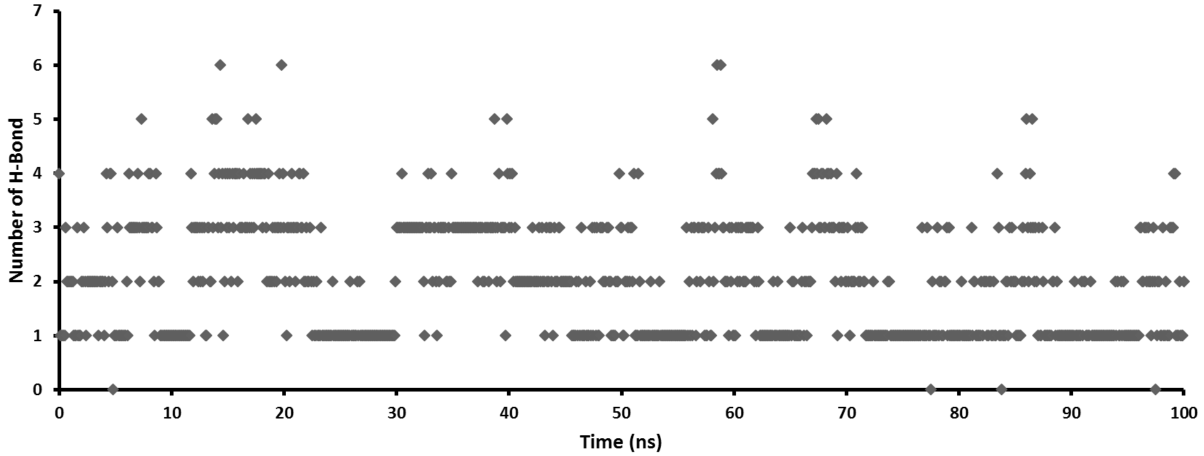
Number of H-Bond
Time (ns)
0
1
2
3
4
5
6
7
0
10
20
30
40
50
60
70
80
90
100

(e)

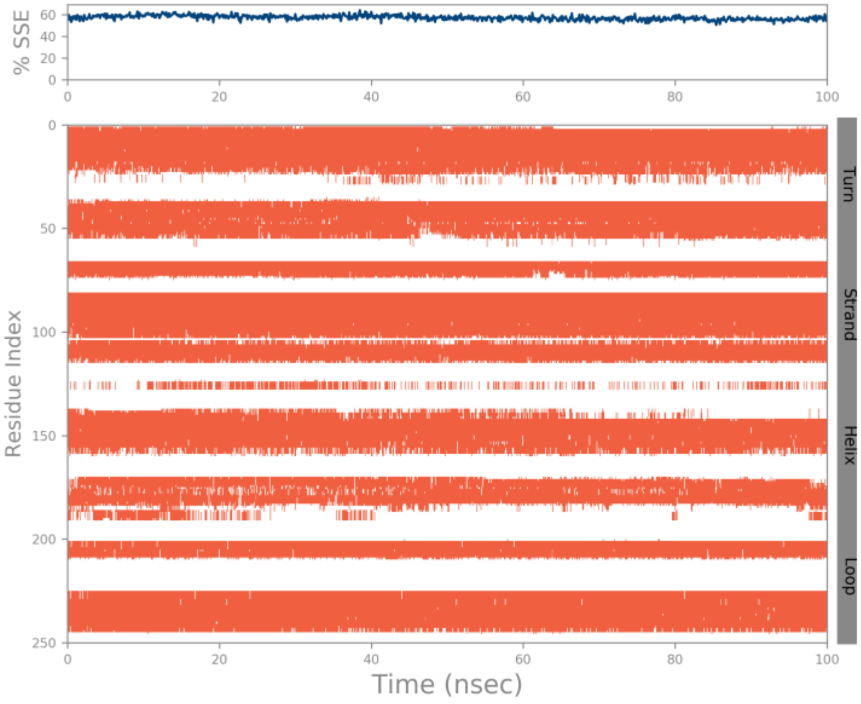
% SSE
Residue Index
Time (nsec)
Turn
Strand
Helix
Loop

Figure S45. (a) Root mean squared fluctuations (RMSFs) of Cα-atoms along with contacts (green-colored vertical bars) of 31879059 in complex with AQP-3; (b) Protein ligand contacts histogram throughout the simulation for 31879059 in complex with AQP-3; (c) Protein-ligand interaction profile for 31879059 in complex with AQP-3; (d) Hydrogen bond interaction observed between protein and 31879059 in different frames throughout the simulation; (e) the secondary structure elements (SSE) for protein conformation during the simulation (% of Total SSE calculated were 54.98).

(a)

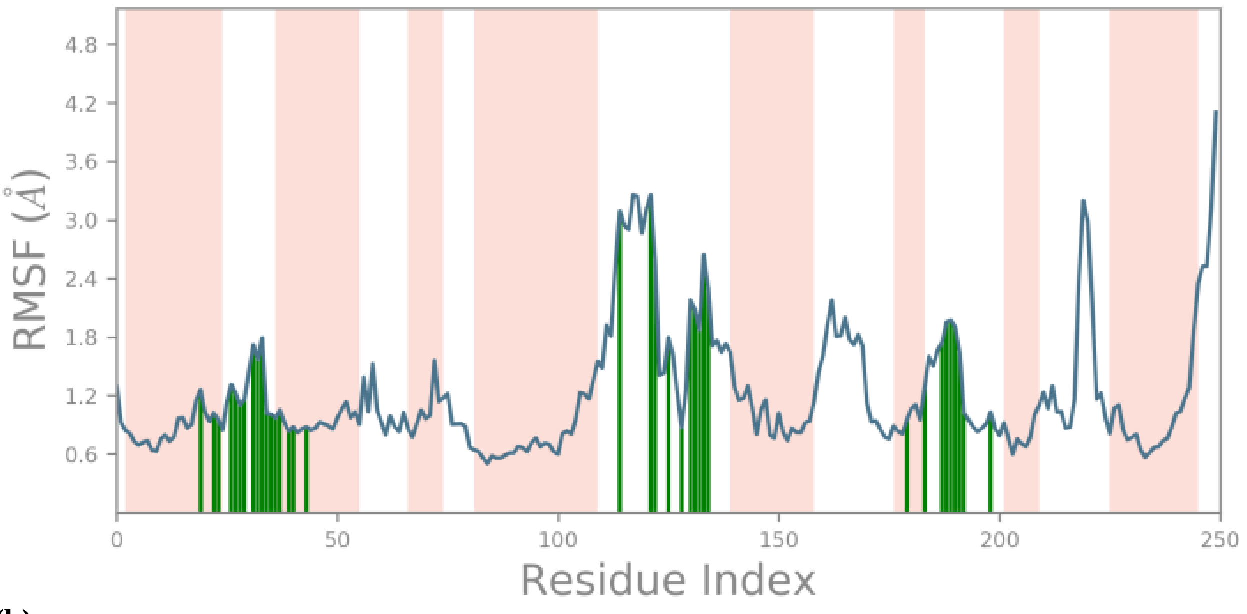

(b)

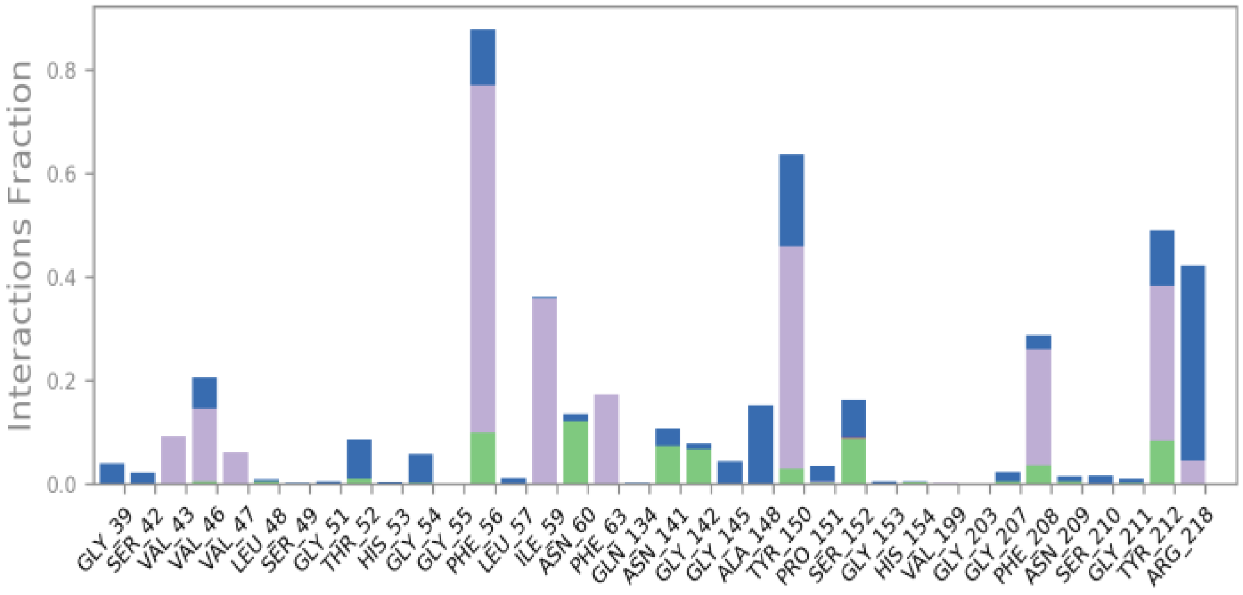

(c)

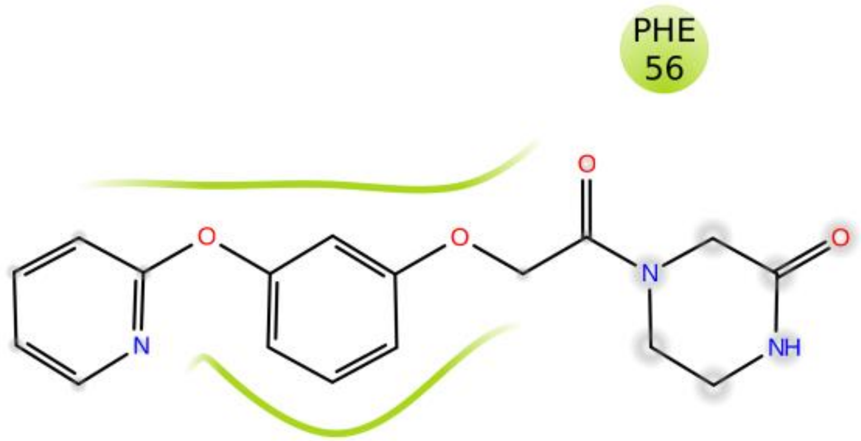

PHE 56
O
O
O
N
N
O
NH

(d)

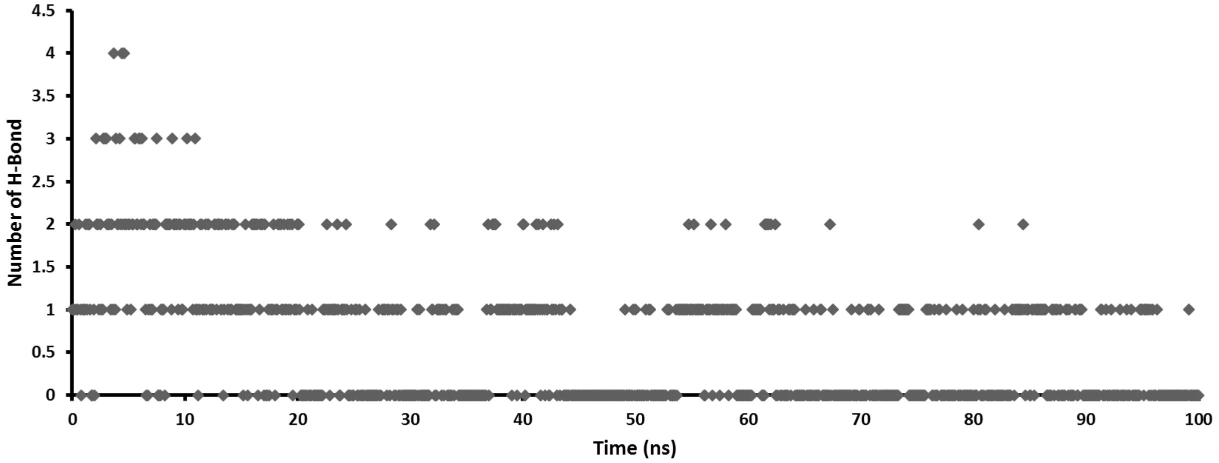

Number of H-Bond
4.5
4
3.5
3
2.5
2
1.5
1
0.5
0
0
10
20
30
40
50
60
70
80
90
100
Time (ns)

(e)

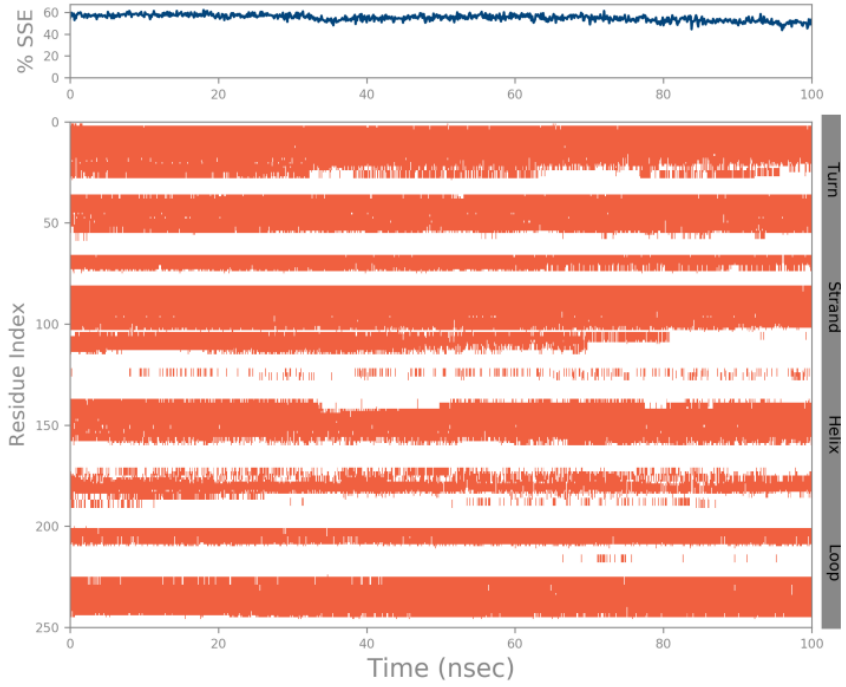

% SSE
60
40
20
0
0
20
40
60
80
100
Residue Index
0
50
100
150
200
250
Turn
Strand
Helix
Loop
Time (nsec)

Figure S46. (a) Root mean squared fluctuations (RMSFs) of Cα-atoms along with contacts (green-colored vertical bars) of 31966421 in complex with AQP-3; (b) Protein ligand contacts histogram throughout the simulation for 31966421 in complex with AQP-3; (c) Protein-ligand interaction profile for 31966421 in complex with AQP-3; (d) Hydrogen bond interaction observed between protein and 31966421 in different frames throughout the simulation; (e) the secondary structure elements (SSE) for protein conformation during the simulation (% of Total SSE calculated were 54.79).

(a)

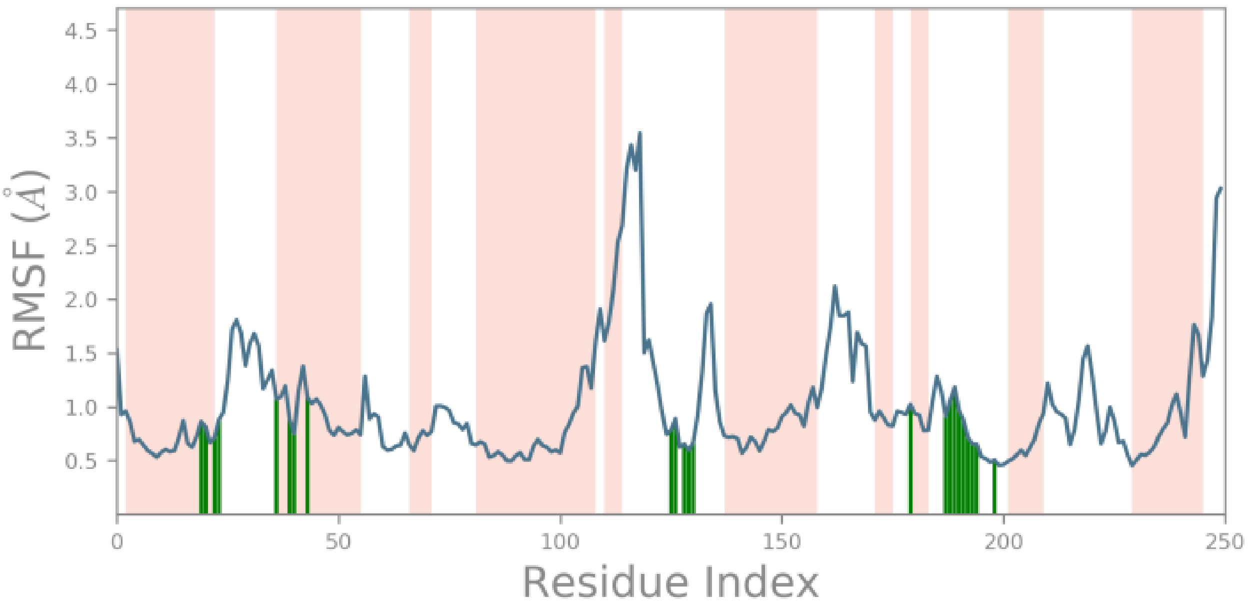

(b)

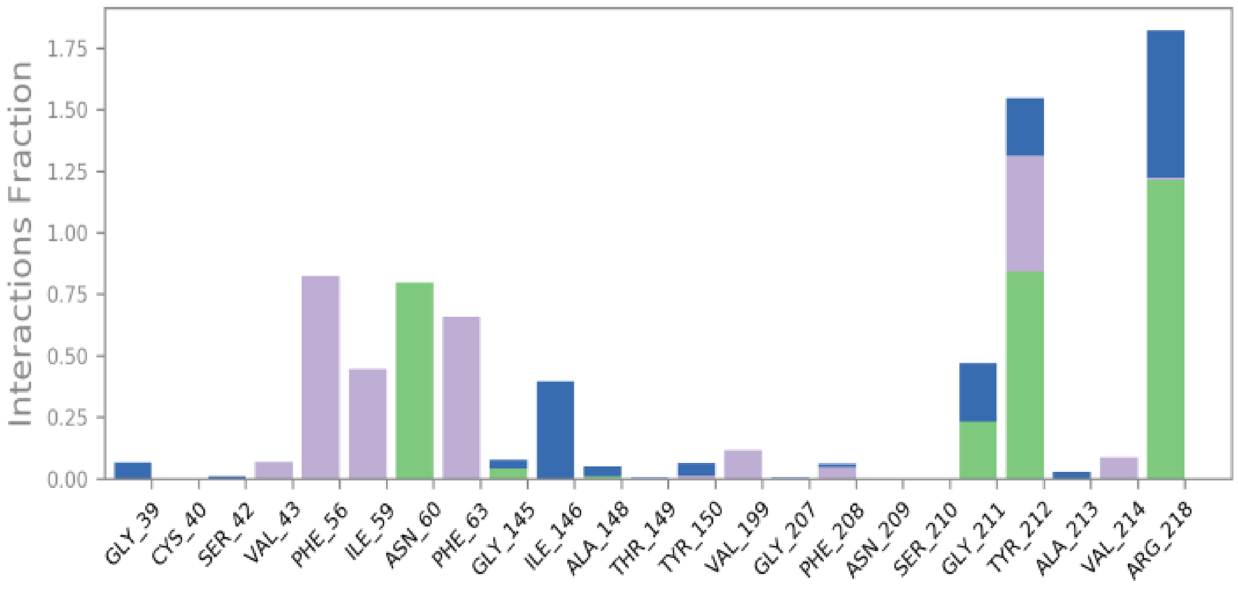

(c)

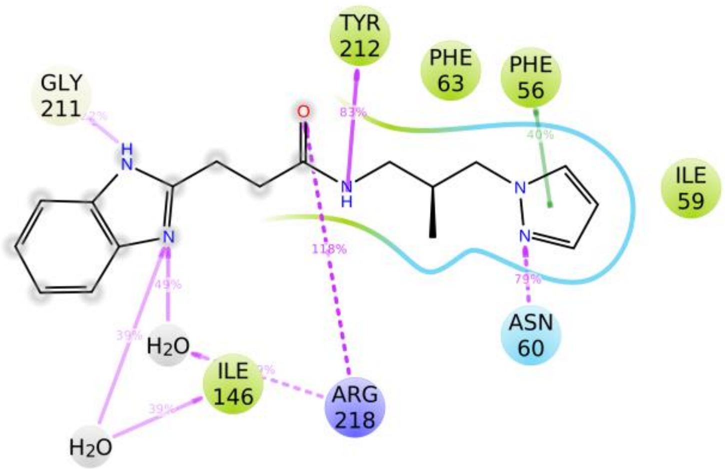

GLY 211
TYR 212
PHE 63
PHE 56
ILE 59
ASN 60
ILE 146
ARG 218
H2O
H2O
83%
40%
118%
79%
49%
39%
39%

(d)

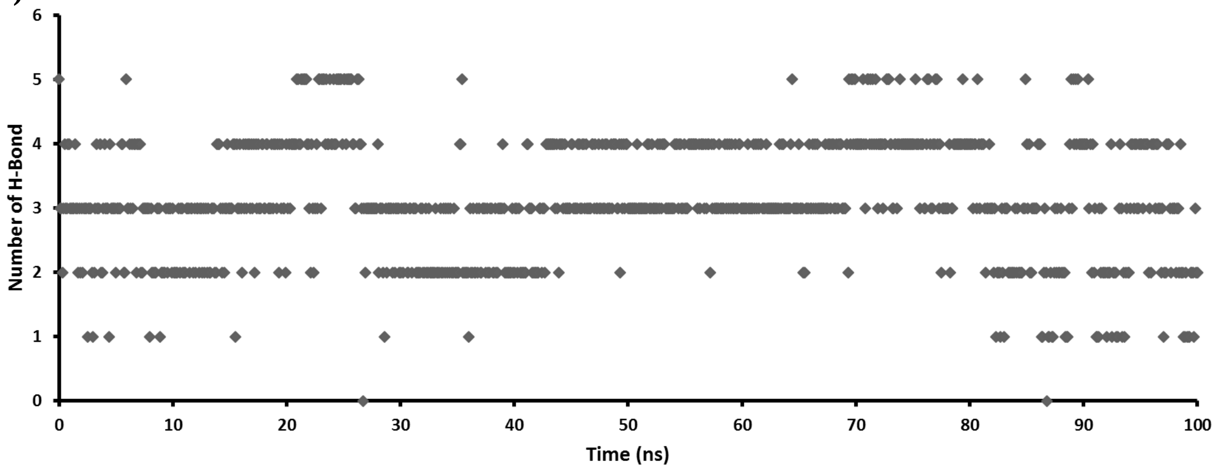

Number of H-Bond
Time (ns)
0
1
2
3
4
5
6
10
20
30
40
50
60
70
80
90
100

(e)

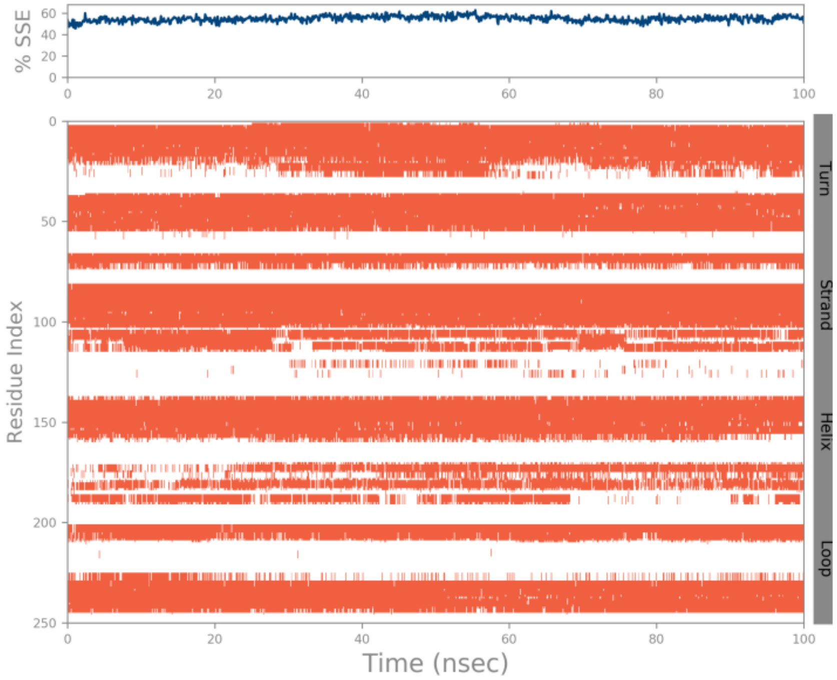

% SSE
Residue Index
Time (nsec)
Turn
Strand
Helix
Loop

Figure S47. (a) Root mean squared fluctuations (RMSFs) of Cα-atoms along with contacts (green-colored vertical bars) of 7658775 in complex with AQP-3; (b) Protein ligand contacts histogram throughout the simulation for 7658775 in complex with AQP-3; (c) Protein-ligand interaction profile for 7658775 in complex with AQP-3; (d) Hydrogen bond interaction observed between protein and 7658775 in different frames throughout the simulation; (e) the secondary structure elements (SSE) for protein conformation during the simulation (% of Total SSE calculated were 50.62).

(a)

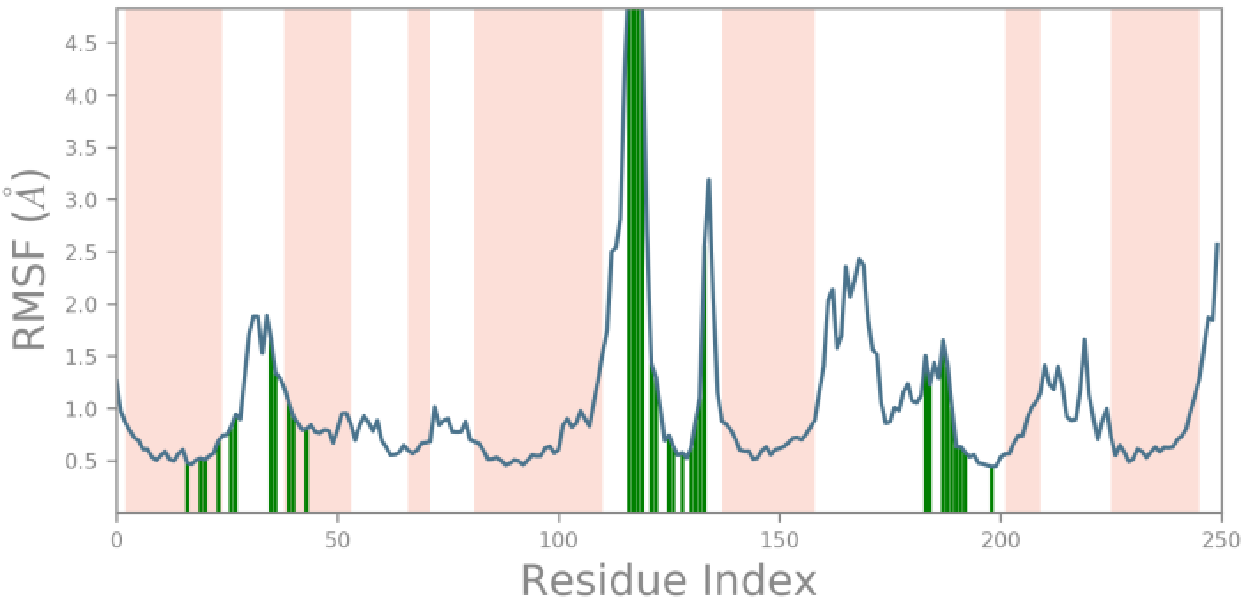

(b)

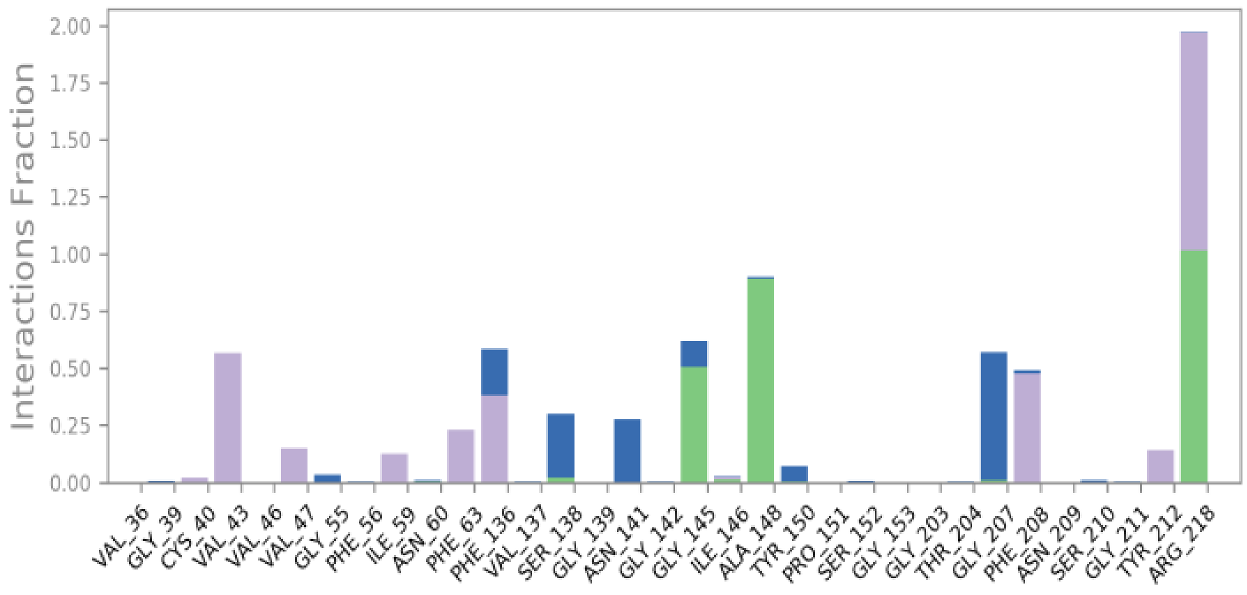

(c)

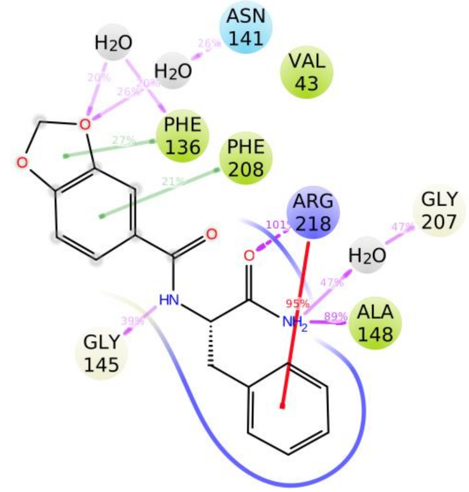
H2O
ASN
141
H2O
VAL
43
PHE
136
PHE
208
ARG
218
GLY
207
H2O
ALA
148
GLY
145
HN
NH2

(d)

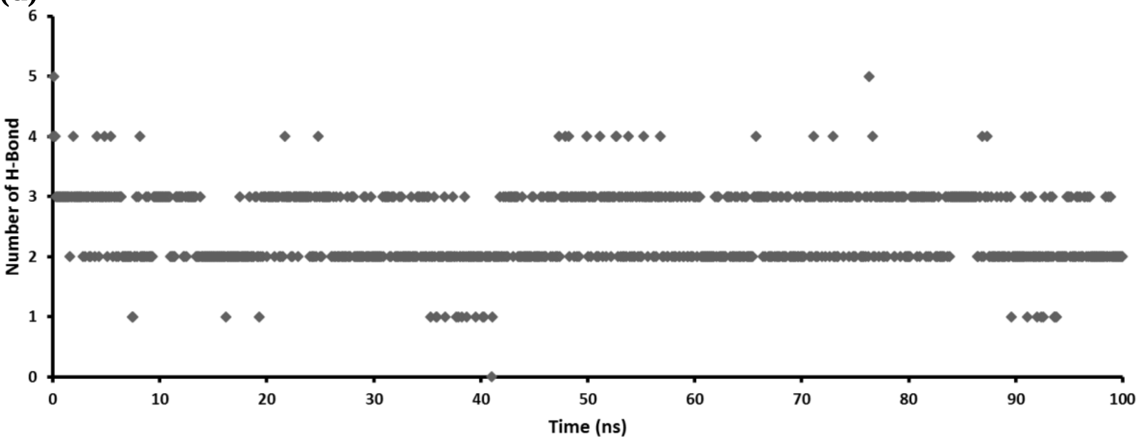
Number of H-Bond
Time (ns)

(e)

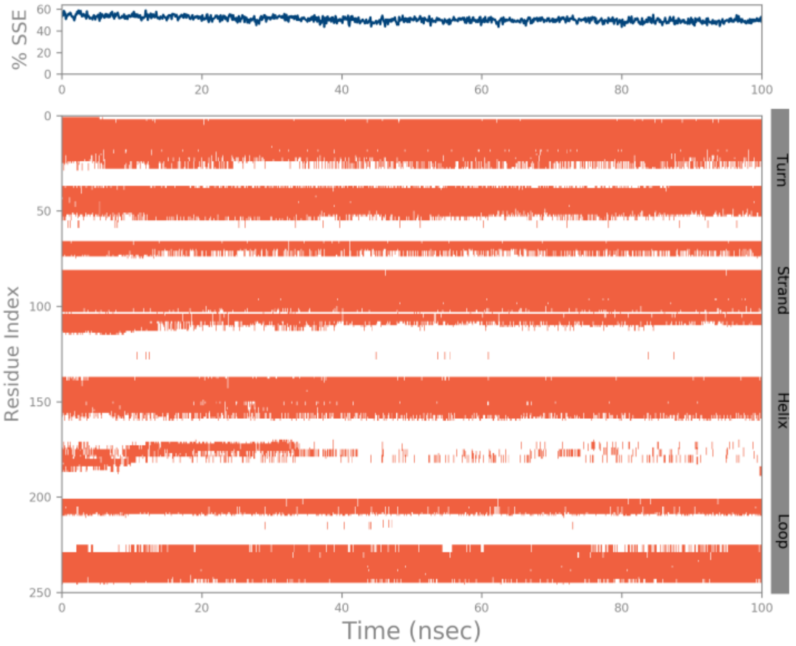
% SSE
Residue Index
Time (nsec)
Turn
Strand
Helix
Loop

Figure S48. (a) Time dependence of root mean squared deviation (RMSD) of Cα-atoms of AQP-3 in complex with 25665268; (b) Root mean squared fluctuations (RMSFs) of Cα-atoms along with contacts (green-colored vertical bars) of 25665268 in complex with AQP-3; (c) Protein ligand contacts histogram throughout the simulation for 25665268 in complex with AQP-3; (d) Protein-ligand interaction profile for 25665268 in complex with AQP-3; (e) Hydrogen bond interaction observed between protein and 25665268 in different frames throughout the simulation; (f) the secondary structure elements (SSE) for protein conformation during the simulation (% of Total SSE calculated were 53.53).

(a)

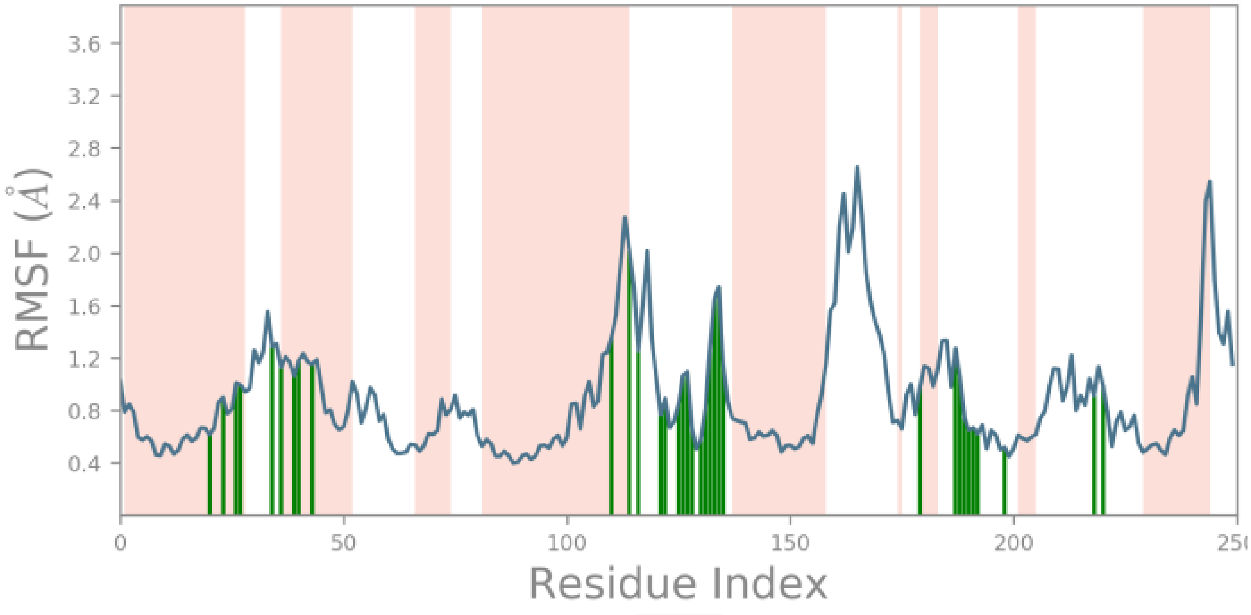

(b)

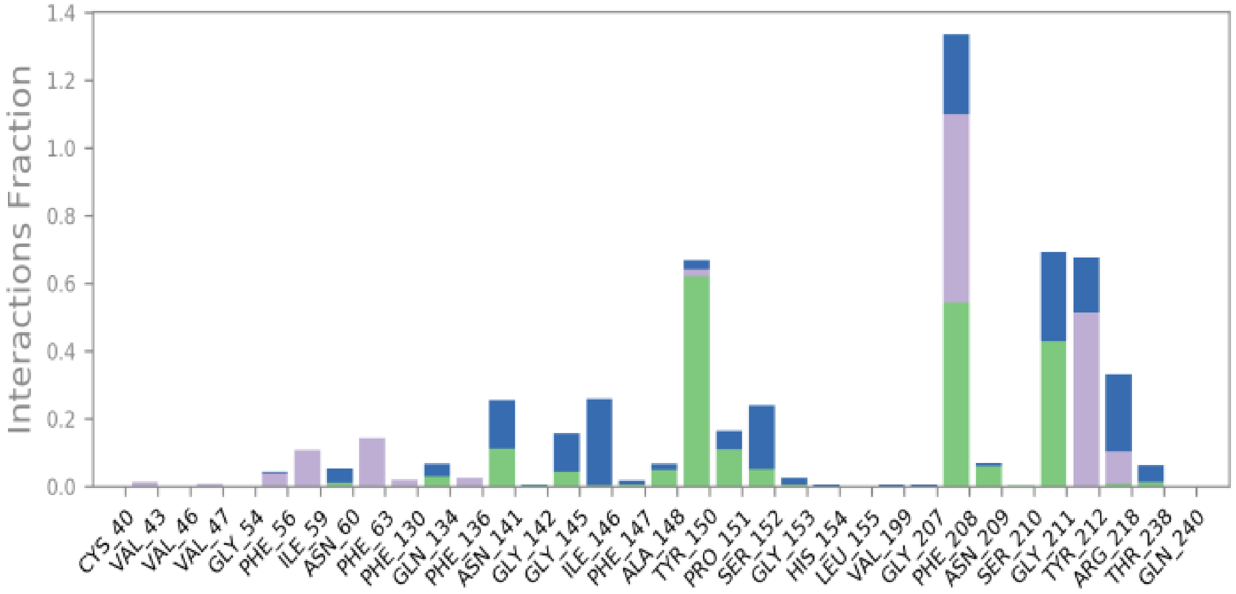

(c)

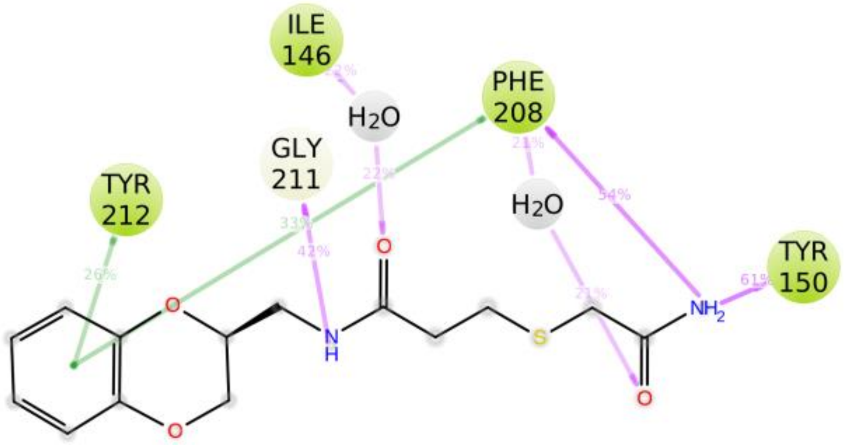
ILE 146
PHE 208
GLY 211
TYR 212
TYR 150
H2O
H2O
54%
61%
33%
42%
22%
26%
NH2
O
N
H
S

(d)

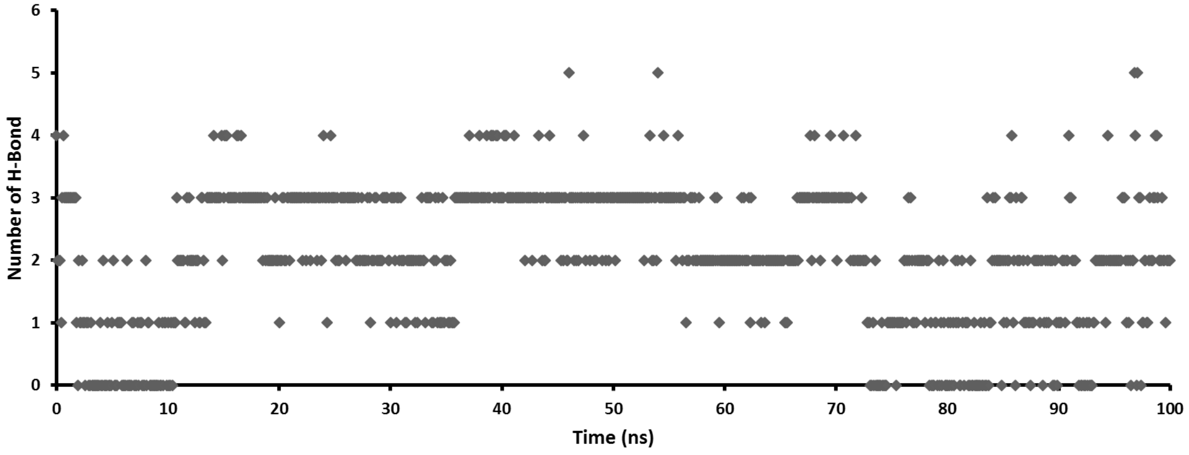
Number of H-Bond
Time (ns)
0
1
2
3
4
5
6
0
10
20
30
40
50
60
70
80
90
100

(e)

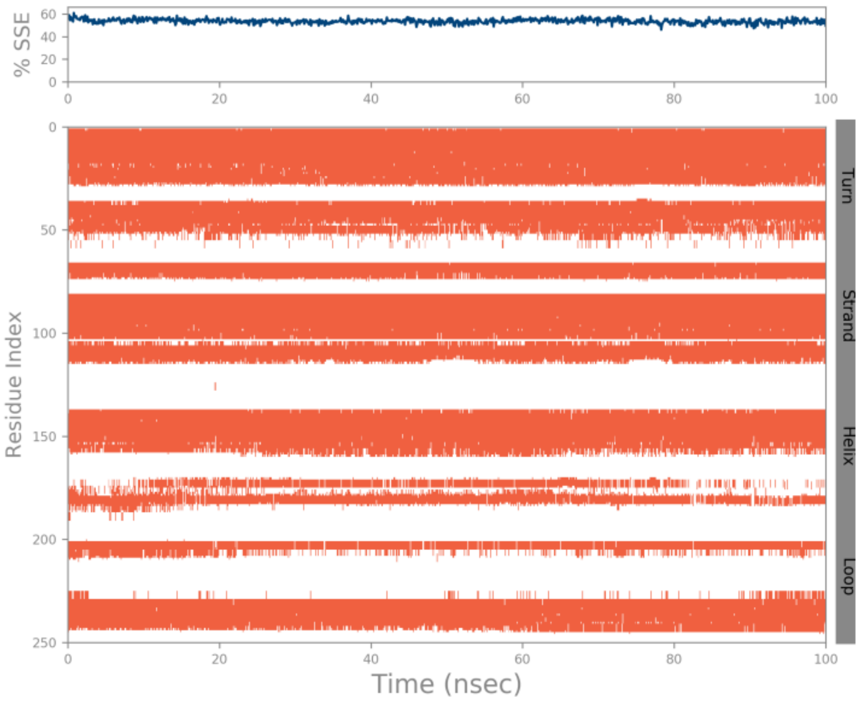
% SSE
Residue Index
Time (nsec)
Turn
Strand
Helix
Loop

Figure S49. (a) Root mean squared fluctuations (RMSFs) of Cα-atoms along with contacts (green-colored vertical bars) of 37101119 in complex with AQP-3; (b) Protein ligand contacts histogram throughout the simulation for 37101119 in complex with AQP-3; (c) Protein-ligand interaction profile for 37101119 in complex with AQP-3; (d) Hydrogen bond interaction observed between protein and 37101119 in different frames throughout the simulation; (e) the secondary structure elements (SSE) for protein conformation during the simulation (% of Total SSE calculated were 52.76).

(a)

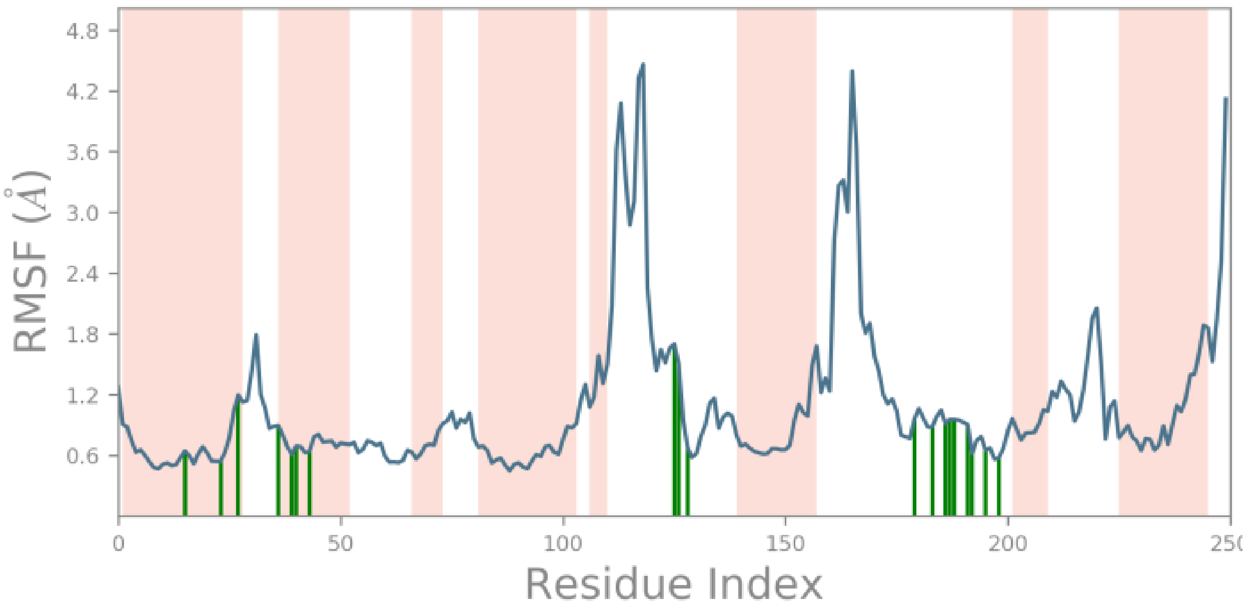

(b)

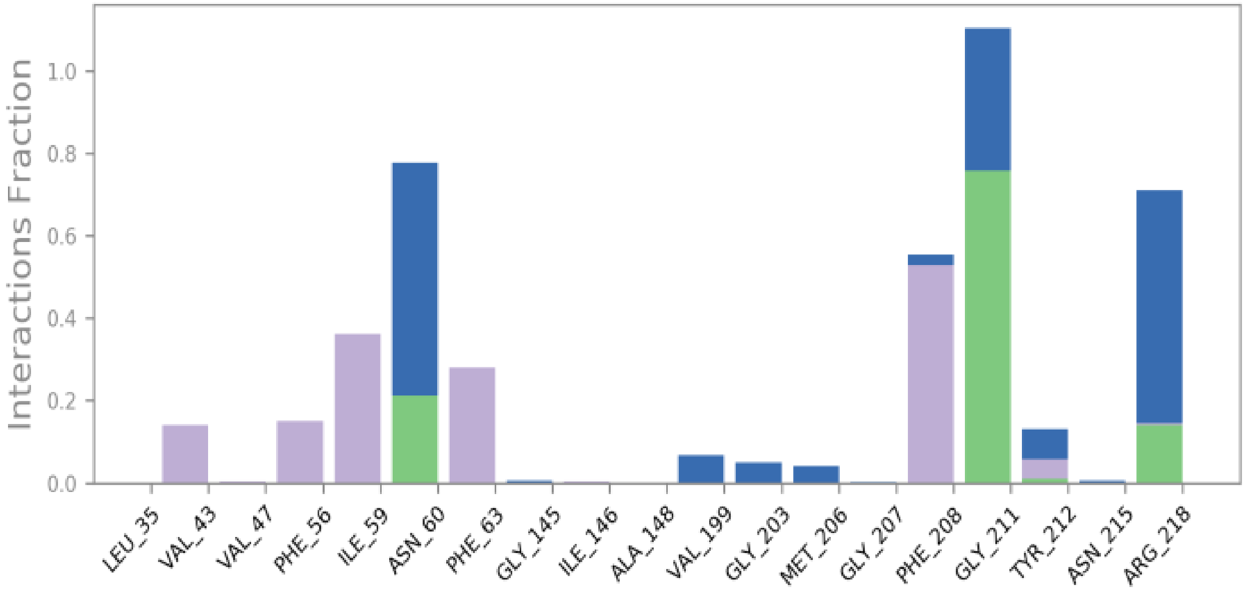

(c)

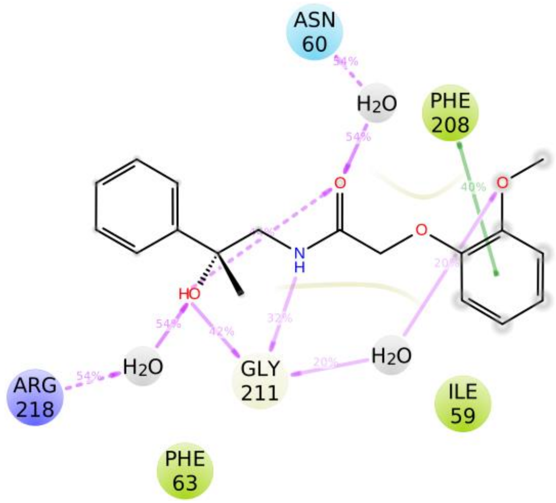
ASN
60
H2O
PHE
208
O
N
H
O
O
HO
H2O
H2O
ARG
218
GLY
211
ILE
59
PHE
63

(d)

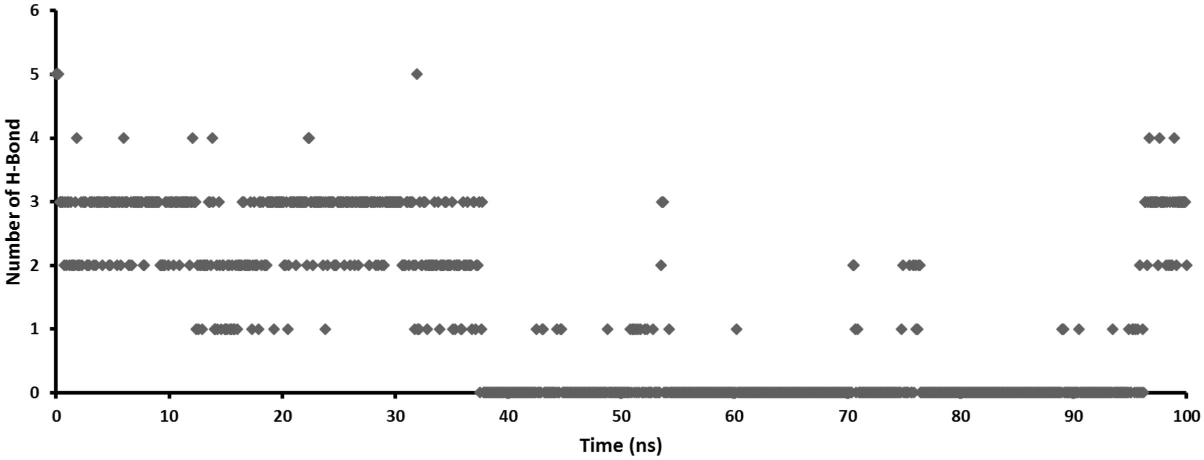
Number of H-Bond
Time (ns)

(e)

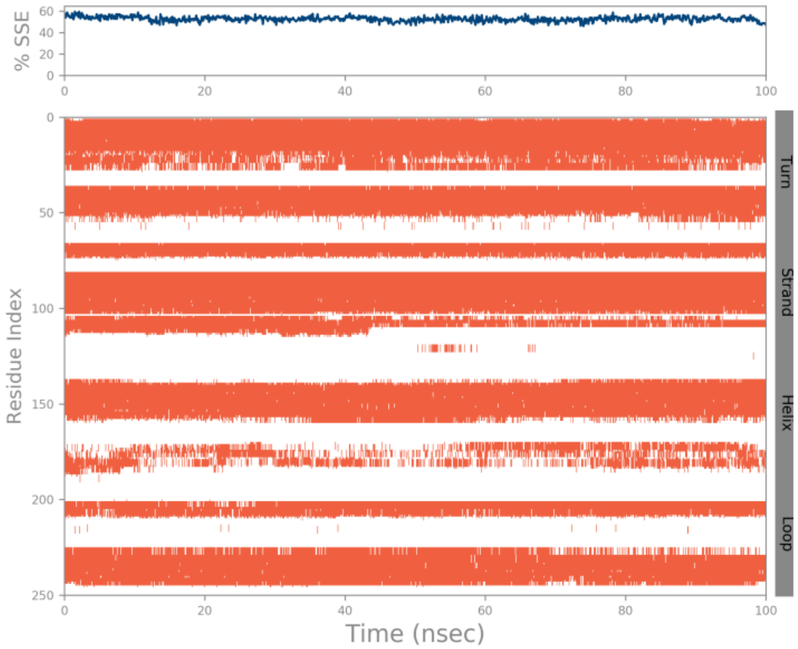
% SSE
Residue Index
Turn
Strand
Helix
Loop
Time (nsec)

Figure S50. Time dependence of root mean squared deviation (RMSD) of ligand (CMPD01, CMPD02, CMPD03, CMPD04, CMPD05) in binding pocket of AQP-3 protein.

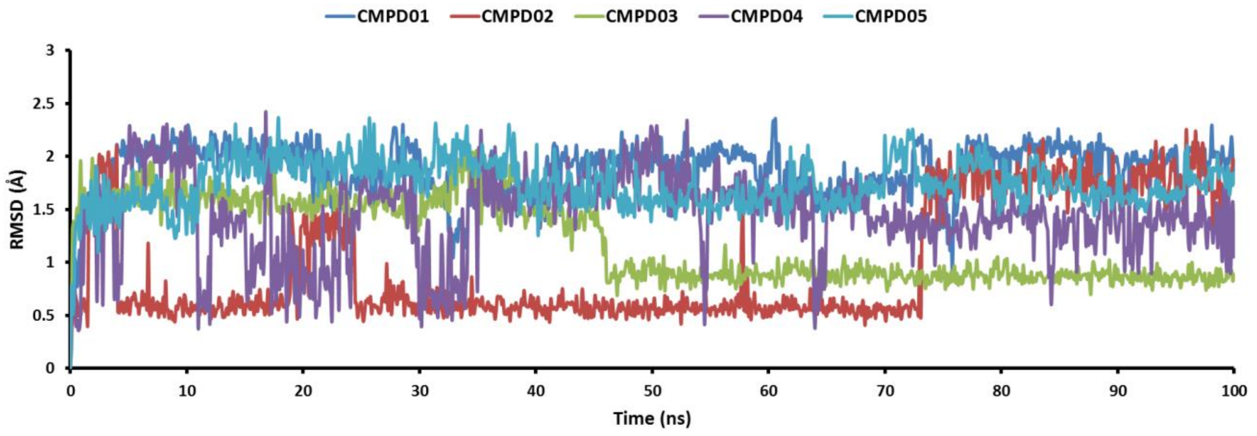

Figure S51. Time dependence of root mean squared deviation (RMSD) of ligand (CMPD06, CMPD07, CMPD08, CMPD09, CMPD10) in binding pocket of AQP-3 protein.

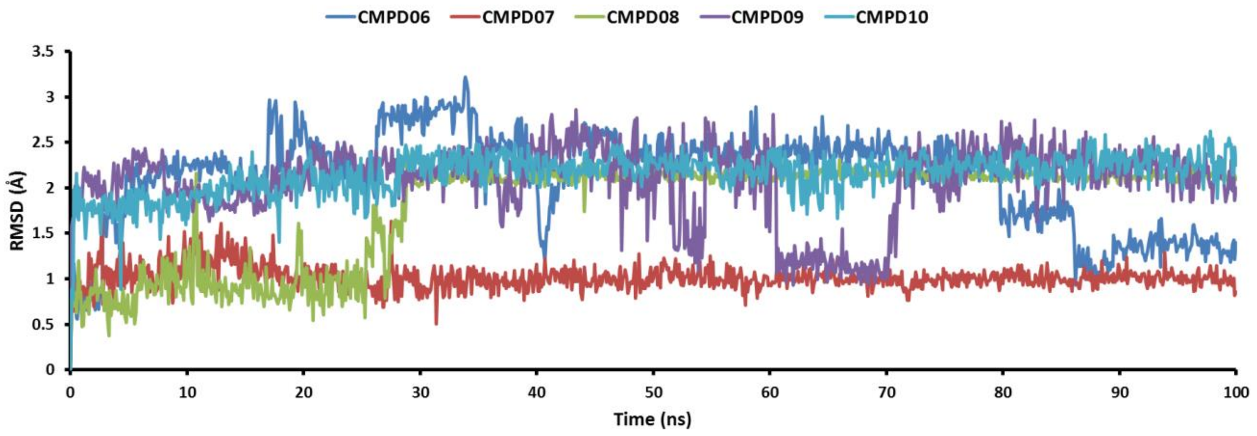

Figure S52. Time dependence of root mean squared deviation (RMSD) of ligand (CMPD11, CMPD12, CMPD13, CMPD14, CMPD15) in binding pocket of AQP-3 protein.

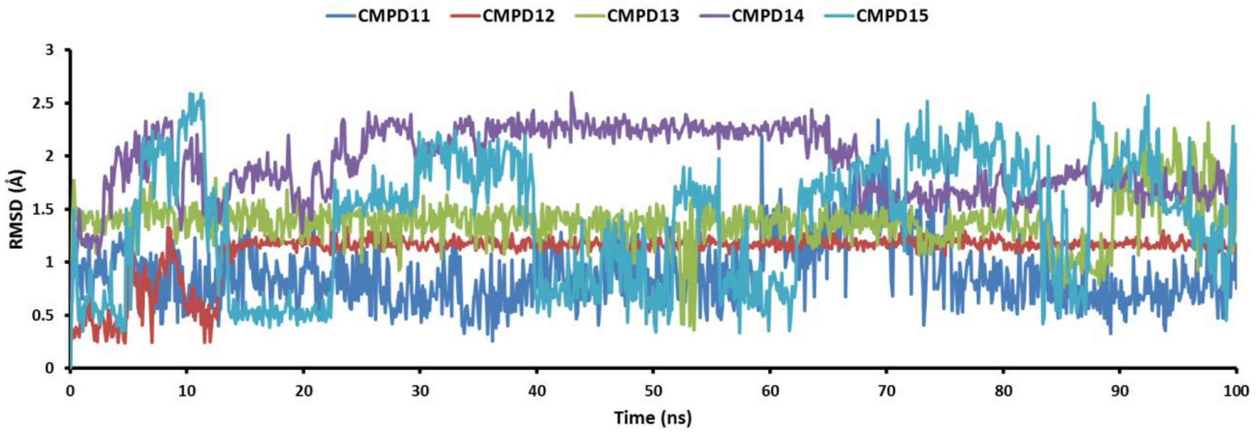

Figure S53. Time dependence of root mean squared deviation (RMSD) of ligand (CMPD16, CMPD17, CMPD18, CMPD19, CMPD20) in binding pocket of AQP-3 protein.

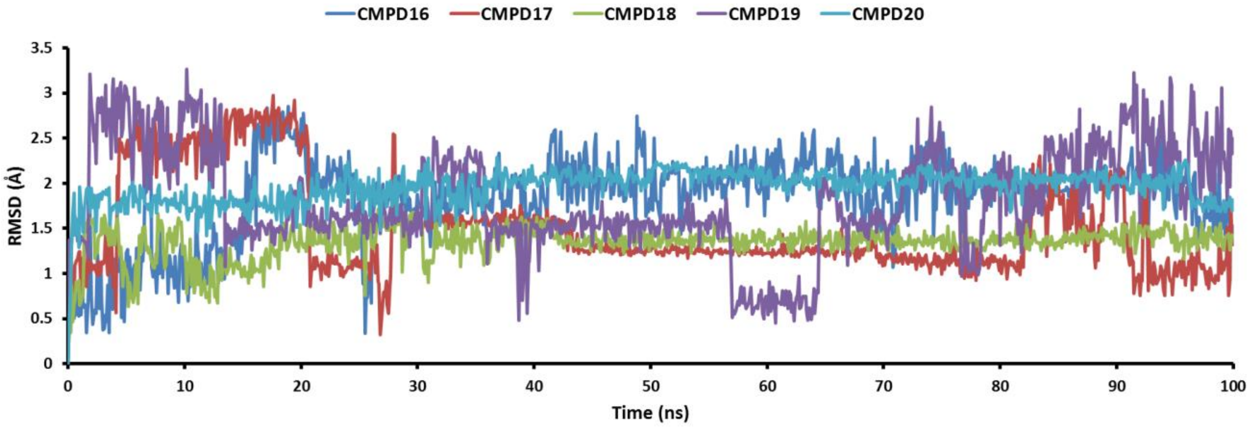

Abbreviations:
CMPD01: C8C
CMPD02: STOCKIN-03432
CMPD03: 25284644
CMPD04: 32927247
CMPD05: 27371521
CMPD06: 2801237
CMPD07: 5633879
CMPD08: 36994203
CMPD09: 16694164
CMPD10: 13477729
CMPD11: 36657947
CMPD12: 36716128
CMPD13: 3325122
CMPD14: 42888719
CMPD15: IP6
CMPD16: 31879059
CMPD17: 31966421
CMPD18: 7658775
CMPD19: 25665268
CMPD20: 37101119

Figure S54: The water-bridge network displayed for selected compound in the binding pocket from the dynamic simulations. The selected compounds their ID and binding energy (MM-GBSA) are as follows; Compound ID 5633879: -74.01±3.21; Compound ID IP6: -68.48±6.00; Compound ID 36657947: -46.58±2.81; Compound ID C8C: -27.28±4.11

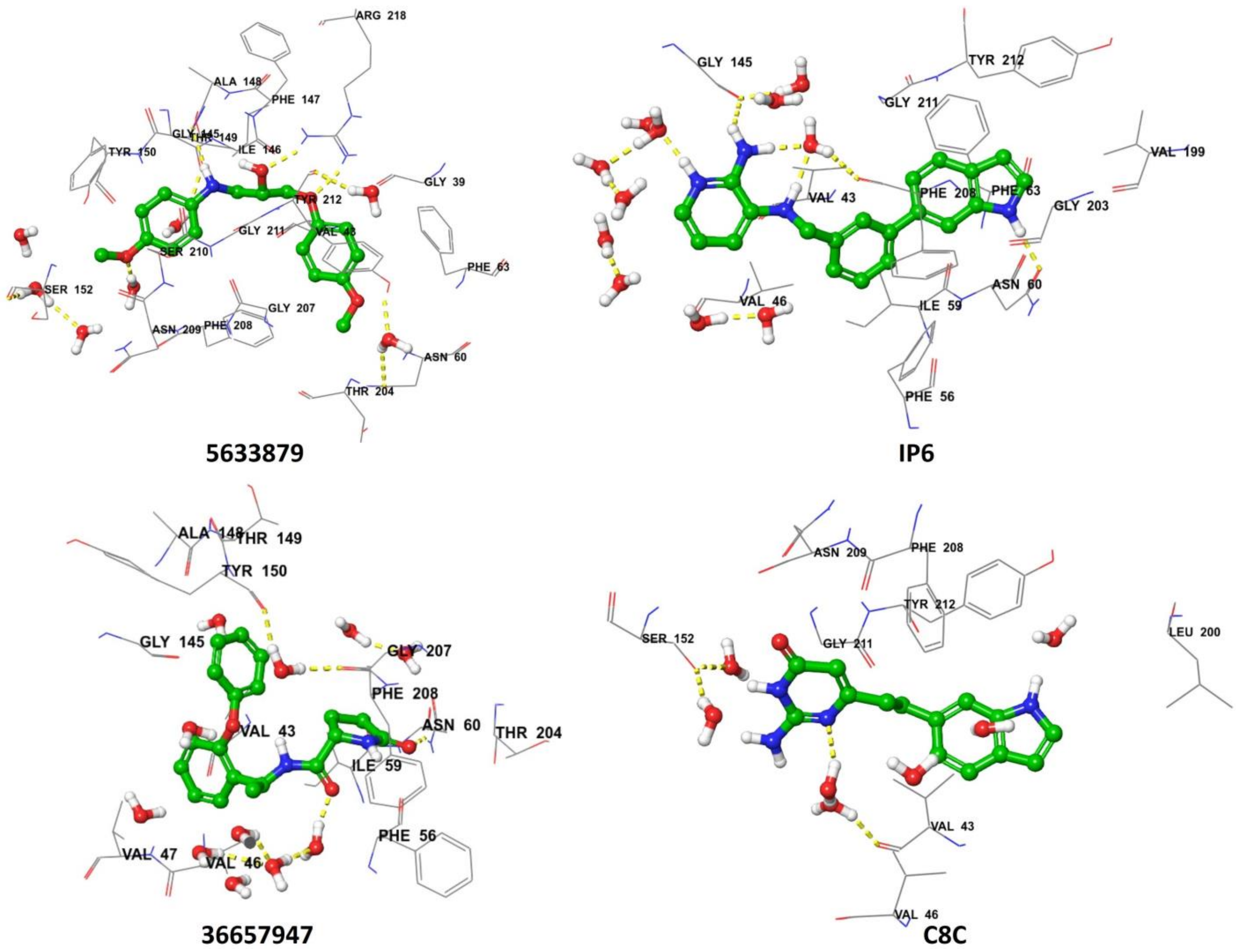

Supplementary Movie-01: Movie depicting the ligand unbinding path from the AQP3 binding pocket.

Movie-01.mp4
